# Effects of plyometric training on strength, explosive performance, and agility in female team-sport athletes: a systematic review and three-level meta-analysis

**DOI:** 10.3389/fphys.2026.1837615

**Published:** 2026-05-05

**Authors:** Zhuo Zeng, Peng Zhang, Chengyu Zhou, Zixin Wang, Junhao Li, Qi Xie, Haoran Li, Dongxu Huang, Yongmin Xie, Aiguo Zhou

**Affiliations:** 1 Strength and Conditioning Training College, Beijing Sport University, Beijing, China; 2 Department of Biomedical Engineering, The Hong Kong Polytechnic University, Hong Kong, Hong Kong SAR, China

**Keywords:** agility, explosive performance, female athlete, plyometric training, strength, team sport

## Abstract

**Introduction:**

Plyometric training (PT) is widely used in team sports, but its outcome-specific effects in female team sport athletes and the influence of key programming variables remain unclear.

**Methods:**

This systematic review and three-level meta-analysis examined the effects of PT on strength, jumping, sprinting, throwing, and agility in female team sport athletes and explored whether training frequency, intervention duration, and ground contacts per session moderated these effects. Randomized and non-randomized controlled trials were identified through database searches and manual screening. A three-level random-effects meta-analysis with cluster-robust variance estimation was performed. Subgroup analyses and meta-regressions examined potential moderators, and certainty of evidence was assessed using GRADE. Thirty-six studies involving 921 participants were included.

**Results:**

PT significantly improved vertical jump (SMD = 0.67), horizontal jump (SMD = 0.70), sprint (SMD = -0.85), throwing (SMD = 0.91), and agility (SMD = -1.09). Effects on strength were inconsistent (*p* = 0.0025), with improvements in upper-body (SMD = 1.09) but not lower-body strength (SMD = 0.08). Competitive level moderated agility outcomes, whereas age was not a consistent moderator. Meta-regression showed no clear associations of training frequency, intervention duration, or ground contacts per session with performance changes. Certainty of evidence ranged from very low to moderate.

**Discussion/conclusion:**

PT can be an effective training strategy for improving multiple outcomes in female team sport athletes, particularly jumping, sprinting, throwing, and agility. However, adaptations appear outcome-specific, and evidence is insufficient to identify programming variables that consistently influence outcomes. Further studies with larger and more diverse samples are needed.

**Systematic Review Registration:**

https://www.crd.york.ac.uk/PROSPERO/view/CRD420261328856, identifier CRD420261328856.

## 1 Introduction

Female team sports, such as soccer, basketball, volleyball, and handball, have seen a global surge in professionalization (Marcelli et al., 2024) and competitive intensity (Pérez Armendáriz et al., 2024; Brassington et al., 2026). Success in these disciplines is heavily predicated on an athlete’s ability to execute explosive movements, including rapid accelerations, high-velocity changes of direction, and maximal vertical or horizontal jumps (Stankovic et al., 2023). These actions require a high capacity for rapid force production and efficient neuromuscular coordination, qualities commonly described as explosive power (Petrušič, 2024). Given the congested competition schedules and the high prevalence of lower-limb injuries (e.g., ACL tears) in female cohorts, identifying time-efficient and robust training interventions to optimize explosive performance is of clear practical importance for practitioners and sports scientists alike (López-Valenciano et al., 2021).

Plyometric training (PT), which is characterized by the use of the stretch-shortening cycle (SSC), is widely regarded as a cornerstone of physical preparation for enhancing power output (Kons et al., 2023). Its benefits are commonly attributed to improved SSC function, including more effective use of elastic energy and neural mechanisms that facilitate rapid transitions from eccentric to concentric muscle action (Fukutani et al., 2020). While the efficacy of PT is well-established in general athletic populations (Slimani et al., 2016; Kons et al., 2023), a more population-specific understanding of its effects in female team sport athletes is still needed, particularly with respect to outcome-specific adaptations and the influence of sport-relevant programming variables (Pardos-Mainer et al., 2021; Lin et al., 2025; Zhao et al., 2025).

Although recent evidence has summarized the effects of PT in female team sport athletes, important limitations remain (Kons et al., 2023; Cao et al., 2024). Existing syntheses have largely focused on broad physical fitness outcomes, particularly jumping, sprinting, and change-of-direction performance, whereas less attention has been given to a more differentiated outcome framework that also includes muscle strength and throwing performance (Lin et al., 2025; Zhao et al., 2025). Moreover, jump-related outcomes have not always been examined with sufficient specificity, although different jump tasks may reflect distinct neuromuscular characteristics and training adaptations (Kons et al., 2023). Furthermore, existing reviews do not always adequately address the statistical dependency introduced when individual studies contribute multiple correlated effect sizes, which may affect the precision and interpretability of pooled estimates (Gucciardi et al., 2022; Pustejovsky and Tipton, 2022). Finally, although some recent reviews have incorporated formal evidence appraisal, the certainty and practical robustness of PT-related findings in female team sport athletes remain incompletely defined (Cao et al., 2024; Lin et al., 2025).

Therefore, the primary objective of this systematic review and three-level meta-analysis was to synthesize and quantify the effects of PT on muscle strength, explosive performance (i.e., vertical jump, horizontal jump, sprint, and throwing), and agility in healthy female team sport athletes. To address methodological limitations in the existing literature, this study applied a three-level meta-analytic model to account for effect-size dependency, examined whether training frequency, intervention duration, and ground contacts per session moderated the pooled effects, and used the GRADE framework to evaluate the certainty of the evidence. In doing so, this review aimed to provide a more precise and practically relevant synthesis to inform PT prescription in female team sport athletes across different developmental stages and competitive levels.

## 2 Methods

This systematic review was conducted in accordance with the Preferred Reporting Items for Systematic Reviews and Meta-Analyses (PRISMA 2020) guidelines (Page et al., 2021). The completed PRISMA 2020 checklist is available in **Appendix A**. The review protocol was registered in the International Prospective Register of Systematic Reviews (PROSPERO; Registration ID: CRD420261328856; date of access: 28 February 2026).

### 2.1 Information sources and search strategy

A systematic literature search was conducted in six English-language (PubMed, Web of Science, Cochrane Library, Ebsco-Medline, Ebsco-SPORTdiscus, Embase) and three Chinese-language (CNKI, VIP, WanFang) databases. The initial search was finalized on 30 April 2025 and an updated search was run on 10 March 2026 to capture the most recent publications.

The search strategy was based on the PICOS framework, combining terms for the target population (e.g., “female”, “women”) and the intervention (e.g., “plyometric”, “stretch shorten cycle exercise”). No language restrictions were applied to the English-language databases, while Chinese-language databases were searched using Chinese terms. To maximize literature retrieval, the primary database search was supplemented by secondary search techniques, including screening the reference lists of included studies and citation tracking. The complete, detailed search strategy is available in **Appendix B**.

### 2.2 Selection process

Following the removal of duplicates using EndNote X9 (Clarivate, Philadelphia, PA, USA), the titles and abstracts of all unique records were screened independently by two reviewers (Z.Z., Z.P.). This screening was based on the predefined eligibility criteria (see Section 2.3). The same two reviewers then independently assessed the full texts of potentially eligible studies for final inclusion. At both screening stages, any disagreements were resolved through discussion or, if necessary, adjudicated by a third reviewer (Z.C.Y.).

### 2.3 Eligibility criteria

To more clearly evaluate the independent benefits of PT on physical performance in female team sport athletes, studies employing complex concurrent experimental interventions as control conditions were excluded from this meta-analysis. Only studies using regular sport-specific training or passive control conditions as the comparison condition were eligible for inclusion. This methodological choice aimed to prevent the introduction of confounding effects and increased heterogeneity that might arise from alternative active intervention controls, thereby allowing us to better focus on the potential adaptations driven specifically by the stretch shortening cycle during plyometric exercises. The detailed inclusion and exclusion criteria were meticulously defined using the PICOS framework, which includes population, intervention, comparison, outcome, and study design, and are presented in **Table 1**.

**Table 1:** Studies eligibility criteria.

Dimension	Inclusion	Exclusion
P	Healthy female athletes participating in competitive team sports such as basketball, soccer, volleyball, or handball	Male athletes, individual sport athletes, sedentary individuals, and those with musculoskeletal injuries or cardiovascular diseases.
I	PT programs involving jump, sprint, or ballistic exercises conducted over a continuous period	Acute single session interventions and complex concurrent training where the isolated effects of plyometrics cannot be determined (e.g., combined strength and jump interventions)
C	Active control groups performing regular sport specific training or passive control groups maintaining daily activities	Control groups performing alternative experimental training modalities such as heavy resistance lifting or electrostimulation
O	Direct measures of muscle strength, explosive performance including jumping, sprinting, throwing, and agility tests	Studies reporting only physiological markers, psychological scales, or proxy measures without direct physical performance data
S	Randomized controlled trials and non randomized controlled trials published in peer reviewed journals	Case reports, reviews, meta analyses, conference abstracts, theses, and non peer reviewed grey literature

P, participants; I, intervention; C, control; O, outcome; S, study design.

### 2.4 Data extraction

Data extraction was conducted by the same two reviewers (Z.Z., Z.P.) who performed the screening, using a customized Microsoft Excel worksheet prepared before the full-text review. The following information was extracted independently: author and publication details, study design and characteristics, participant demographics (including sport type and competitive level), intervention protocols (including training frequency and duration), and outcome assessments related to strength, explosive performance, and agility. Notably, multiple effect sizes were extracted from single studies whenever a study reported data on more than one eligible outcome or utilized multiple intervention groups, which provided the necessary data structure for the three-level meta-analysis. Accuracy was verified by a third reviewer (Z.C.Y.), and disagreements were resolved through consultation with a fourth independent researcher (L.H.R.).

For studies with missing numerical data or outcomes presented only in graphical format, corresponding authors were contacted for clarification. If no response was received, quantitative data were extracted from graphs using WebPlotDigitizer (version 4.1, Ankit Rohatgi, Belmont, CA, USA). Regarding participant data, an intention-to-treat approach was prioritized when available; otherwise, data for participants who completed the protocol were utilized. Studies for which data remained unavailable after these steps were excluded from the meta-analysis.

### 2.5 Data processing

For the quantitative analysis, the mean, standard deviation (SD), and sample size were extracted for each group at both pre- and post-intervention timepoints. These values were used to calculate the mean change from baseline and its corresponding standard deviation for subsequent pooling of effect sizes.

The ${\text{MD}}_{\text{diff}}\;$, defined as the difference between the post-intervention mean and the pre-intervention mean, was calculated as Equation 1

(1) $${\text{MD}}_{diff}={\text{M}}_{post}-{\text{M}}_{pre}$$

where ${\text{MD}}_{\text{diff}}\;$ is the raw mean difference, ${\text{M}}_{\text{post}}$ is the reported mean post-intervention, and ${\text{M}}_{\text{pre}}$ is the reported mean pre-intervention.

When only confidence intervals were reported, standard deviations were calculated using the Equation 2:

(2) $$\text{SD}=\sqrt{\text{N}}\;\frac{{\text{CI}}_{\text{high}}-{\text{CI}}_{\text{low}}}{2\text{t}}$$

where SD is the standard deviation, N is the group sample size, ${\text{CI}}_{\text{high}}\;$ is the upper limit of the confidence interval, ${\text{CI}}_{\text{low}}$ is the lower limit of the confidence interval, and t is the t distribution with N − 1 degrees of freedom the respective confidence level.

The ${\text{SD}}_{\text{diff}}$ was calculated using the Equation 3 provided in the Cochrane Handbook (Guyatt et al., 2008):

(3) $${\text{SD}}_{diff}=\sqrt{{\text{SD}}_{pre}2^{+}{\text{SD}}_{post}2^{-}2\text{r}\times {\text{SD}}_{pre}\times {\text{SD}}_{post}}$$

where ${\text{SD}}_{\text{diff}}\;$ is the standard deviation of the difference in means, ${\text{SD}}_{\text{pre}}$ is the standard deviation from pre-intervention, and ${\text{SD}}_{\text{post}}\;$ is the standard deviation from post-intervention.

As the original studies did not report Pearson’s correlation coefficients (r) for pre- and post-intervention outcomes, we assumed r = 0.5 based on recommendations from the Cochrane Handbook (Cumpston et al., 2019). Sensitivity analyses where r was varied between 0.3 and 0.7 confirmed that the primary pooled estimates were robust. The Equation 4 was provided as follows:

(4) $$\text{r}=\frac{{\text{SD}}_{\text{pre}}2^{+}{\text{SD}}_{\text{post}}2^{-}{\text{SD}}_{\text{change}}2^{2}}{\times {\text{SD}}_{\text{pre}}\times {\text{SD}}_{\text{post}}}$$

### 2.6 Risk of bias and quality of methods assessment

Risk of bias was independently assessed by two reviewers (Z.C.Y. and Z.P.) using the Cochrane Risk of Bias 2 (RoB 2) tool, which evaluates bias across five domains: bias arising from the randomization process, bias due to deviations from intended interventions, bias due to missing outcome data, bias in measurement of the outcome, and bias in selection of the reported result. Discrepancies were resolved through discussion, with unresolved cases adjudicated by a third reviewer (Z.Z.). For non-randomized studies, bias was assessed using the Risk of Bias in Non-Randomized Studies of Interventions (ROBINS-I) tool, covering seven domains: confounding, participant selection, intervention classification, deviations from intended interventions, missing data, outcome measurement, and selection of reported results.

### 2.7 Statistical analysis

All statistical analyses were conducted in R (version 4.5.0, R Foundation for Statistical Computing, Vienna, Austria) (Viechtbauer, 2010).

#### 2.7.1 Overall analytical model

To account for the fact that several included studies reported multiple experimental groups or outcomes, a conventional two-level meta-analysis could violate independence assumptions and overestimate precision (Van den Noortgate et al., 2013). Therefore, a three-level random-effects model was employed (Assink and Wibbelink, 2016), which partitions variance into sampling error (Level 1), within-study variance (Level 2), and between-study variance (Level 3), thereby accounting for effect-size dependency and hierarchical data structures (Cheung, 2019). A correlation coefficient of ρ = 0.6 was assumed for the variance-covariance matrix of dependent effect sizes.

The model’s robustness was further enhanced using cluster-robust variance estimation (CRVE) with small-sample corrections (specifically, the CR2 adjustment proposed by Bell and McCaffrey (McCaffrey and Bell, 2003)) to handle correlated outcomes (Pustejovsky and Tipton, 2022). This adjustment provides more robust standard errors and wider, more conservative confidence intervals, which is crucial when the number of included studies is small (Tipton, 2015). All available effect sizes were retained rather than averaged or discarded, improving statistical power and precision (Assink and Wibbelink, 2016). Model parameters were estimated by restricted maximum likelihood (REML). To verify the stability of parameter estimates, results were additionally cross-checked against maximum likelihood (ML) estimation (Borenstein et al., 2010).

#### 2.7.2 Effect size and heterogeneity assessment

The effect size was the standardized mean difference (SMD), calculated as Hedges’ g to correct for potential bias in small samples. Effect sizes were interpreted as trivial (< 0.2), small (0.2-0.5), medium (0.5-0.8), or large (> 0.8) (Nagashima et al., 2019). Between-study heterogeneity was assessed using Cochrane’s Q, I^2^, τ^2^, and τ, with 95% confidence intervals (CI) and prediction intervals (PI) to quantify dispersion (Nakagawa et al., 2017). I^2^ served as the primary heterogeneity index and was interpreted as low (0-25%), moderate (25-50%), substantial (50-75%), or considerable (75-100%) (Cumpston et al., 2019). Lastly, the statistical power of pooled estimates was assessed and visualized using sunset plots via the “metaviz” package (Quintana, 2023).

#### 2.7.3 Moderator and sensitivity analyses

To explore potential sources of heterogeneity when I^2^ > 25%, subgroup analyses and meta-regressions were performed based on participant characteristics, intervention characteristics, and training protocols. Meta-regressions were conducted using continuous variables to examine the influence of (1) the ground contacts per session, (2) weekly training frequency, and (3) total intervention duration in weeks. Subgroup analyses were implemented for categorical moderators including (1) age and (2) level. In addition, subgroup analyses based on test type were performed for specific outcomes: for strength, tests were categorized as upper-limb or lower-limb performance, whereas for vertical jump performance, tests were categorized as countermovement jump (CMJ), squat jump (SJ), and drop jump (DJ).

Sensitivity analyses were conducted using leave-one-out methods to detect influential studies. In addition, because the original studies did not report pre-post Pearson’s correlation coefficients, sensitivity analyses were performed by varying the assumed correlation coefficient (r) from 0.3 to 0.7 to examine the robustness of the pooled estimates. Publication bias was examined using contour-enhanced funnel plots and Egger’s regression test, with *p* > 0.05 indicating no statistically significant funnel plot asymmetry. For outcomes with significant Egger’s test results (*p* < 0.05), trim-and-fill analyses were additionally conducted as a supplementary assessment of the potential influence of small-study effects or publication bias on the pooled estimates (Egger et al., 1997).

### 2.8 Certainty of the evidence

The Grading of Recommendations Assessment, Development, and Evaluation (GRADE) methodology was used to assess the certainty of evidence, categorizing it as “high,” “moderate,” “low,” or “very low” (Schünemann et al., 2019). Two researchers independently conducted the GRADE assessment, resolving discrepancies through consensus (Z.P., Z.Z.). The assessment considered five domains: (1) For risk of bias, downgrading was considered according to the RoB 2 and ROBINS-I judgments of the contributing studies, with greater concern assigned when limitations were judged to be serious across the evidence base (Guyatt et al., 2011d); (2) For inconsistency, downgrading was considered based on the magnitude and pattern of between-study heterogeneity, with I2 values used as supportive indicators; heterogeneity above 25% was interpreted as suggesting potential inconsistency, and values above 75% as indicating substantial inconsistency (Guyatt et al., 2011b); (3) For indirectness, downgrading was considered when the available evidence did not directly correspond to the population, intervention, comparator, or outcomes of interest; (4) For imprecision, downgrading was considered when statistical power was limited (< 80%), when pooled estimates were unstable, or when the direction or magnitude of the effect remained uncertain (Guyatt et al., 2011a); (5) For publication bias, downgrading was considered when there was evidence of small-study effects or publication bias, including a significant Egger’s test (*p* < 0.05) (Guyatt et al., 2011c).

## 3 Results

### 3.1 Study selection

A literature search was conducted in six English-language databases (PubMed, Web of Science, Cochrane Library, EBSCO-MEDLINE, EBSCO-SPORTDiscus, and Embase) and three Chinese-language databases (CNKI, VIP, and WanFang). The initial search, conducted on 30 April 2025, identified 3898 records. After removal of 1132 duplicates, 2766 records were screened by title and abstract, and 29 additional studies were identified through citation tracking and reference list screening. An updated search was conducted on 10 March 2026, but no further eligible studies were identified.

**Figure 1:**
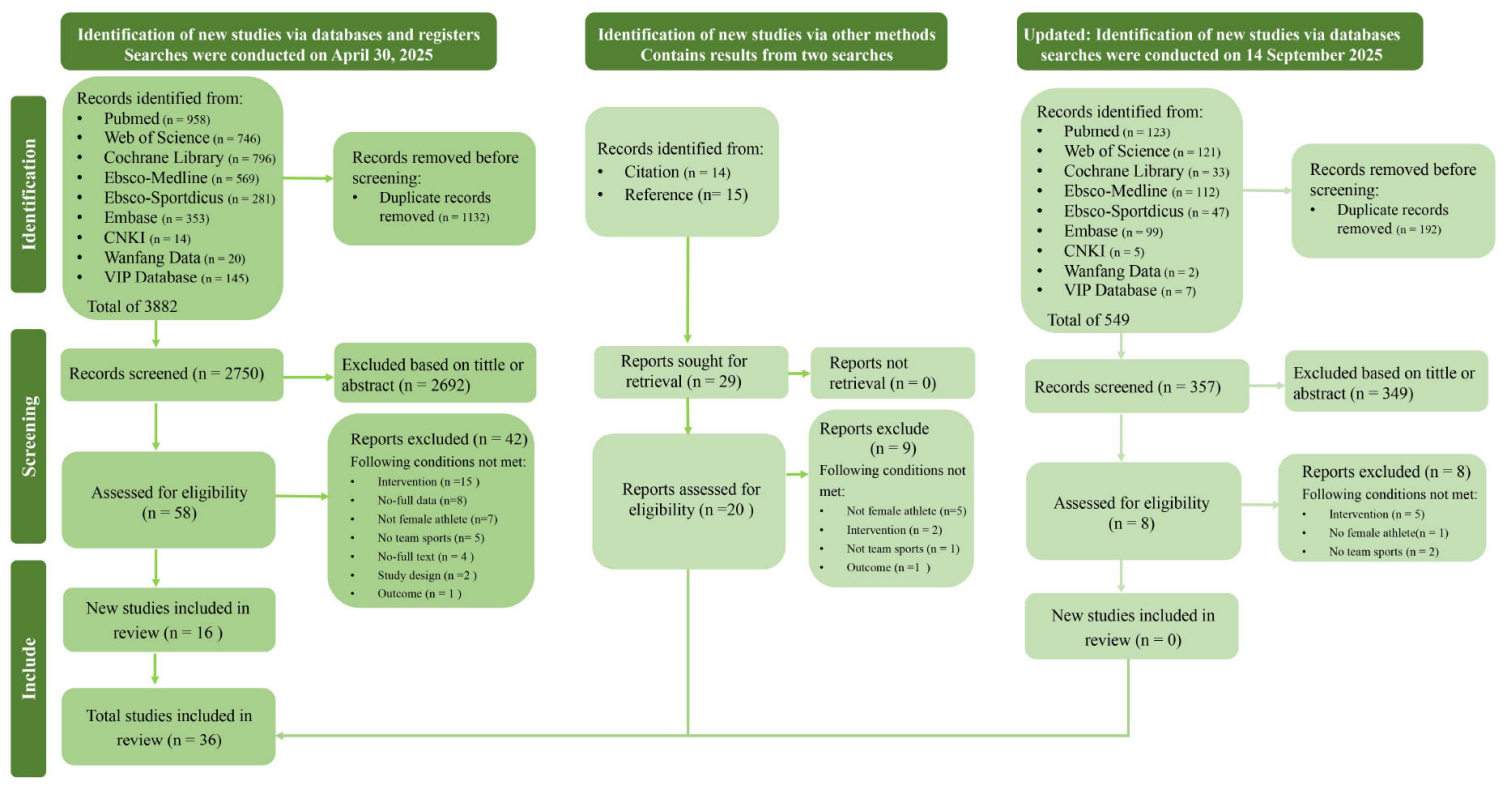
PRISMA flow diagram of study selection process.

Following the full-text assessment, a total of 36 studies met the inclusion criteria and were included in the final meta-analysis (Siegler et al., 2003; Chimera et al., 2004; Wilkerson et al., 2004; Sedano Campo et al., 2009; Rubley et al., 2011; Ozbar et al., 2014; Attene et al., 2015; Ozbar, 2015; Harput, 2016; Kale, 2016; Krističević et al., 2016; Ramírez-Campillo et al., 2016; Trajković et al., 2016; Neves da Silva et al., 2017; Rosas et al., 2017; Ramirez-Campillo et al., 2018; Valadés Cerrato et al., 2018; Cherni et al., 2019; Fischetti et al., 2019; H, 2019; Meszler and Váczi, 2019; Turgut et al., 2019; Cherni et al., 2020; Hammami et al., 2020; Chaabene et al., 2021; Maciejczyk et al., 2021; Q, 2021; Sánchez-Sixto et al., 2021; Vilela et al., 2021; Falch et al., 2022; N, 2022; Nonnato et al., 2022; Gaamouri et al., 2023; Guimarães et al., 2023; Talukdar et al., 2024; ZQ, 2024). The complete study selection process is summarized in the PRISMA flow diagram (**Figure 1**).

### 3.2 Characteristics of included studies

Among the 36 included studies, 26 were randomized controlled trials (RCTs) and 10 were non-randomized controlled trials. The total sample size was 921, with individual study group sizes ranging from 5 to 27 participants, aged 10.5 to 27.25 years. Among them, 483 were adolescents (52.44%) and 438 were adults (47.56%). Most studies provided detailed information on participant characteristics, study design, interventions, and follow-up procedures. A summary of baseline characteristics is provided in **Appendix C** Statistical power diagrams and publication bias funnel plots for the combined outcomes are shown in **Appendixes D**, **E**.

### 3.3 Risk of bias assessment

Risk of bias in RCTs was assessed using the Risk of Bias (RoB) 2.0 tool, with several methodological concerns identified across 26 studies. “Some concerns” were noted for randomization (Domain 1) in 88% of studies, and for selection of the reported result (Domain 5) in 96%, largely due to unclear allocation concealment and lack of pre-registration. Deviations from intended interventions (Domain 2) raised concerns in 38%, while missing outcome data (Domain 3) was a concern in 19%. Additionally, 35% of studies raised concerns regarding outcome measurement (Domain 4).

For 10 included non-randomized studies, bias was assessed using the Risk of Bias In Non-randomized Studies of Interventions (ROBINS-I) tool. No studies were rated as low risk; six (60%) were moderate, and four (40%) were serious risk. Confounding bias was the most prominent, with 30% of studies rated as serious. Bias due to participant selection was serious in 20%, while classification of interventions was rated as low in all studies. The majority of studies were rated low to moderate for bias due to deviations from intended interventions and missing data, with over 80% rated low to moderate for these domains. Outcome measurement bias was largely low, reflecting the objective nature of physical performance tests, and selection of the reported result was mostly rated as moderate.

**Figure 2:**
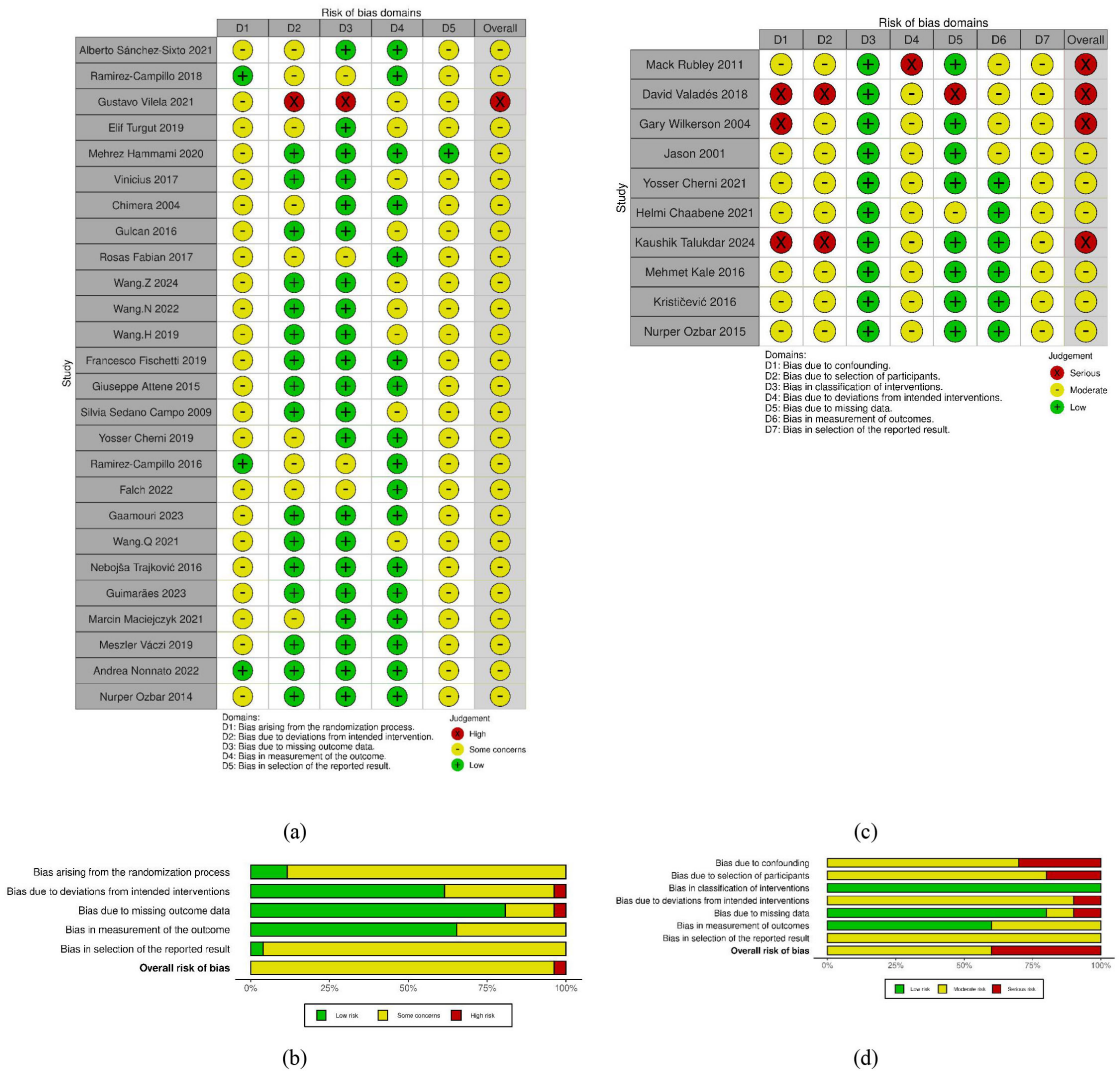
**(a–d)** Risk of bias assessment diagram. **(a)** RoB 2 traffic light plot; **(b)** RoB 2 summary plot; **(c)** ROBINS-I traffic light plot; **(d)** ROBINS-I summary plot.

These limitations could potentially affect the accuracy of effect estimates. Detailed results are provided in **Figures 2a–d**.

### 3.4 Primary meta-analysis results

#### 3.4.1 Strength

A three-level random-effects meta-analysis (REML) was performed to examine the effect of PT on strength in female athletes. Across 11 studies (53 effect sizes), the pooled effect was not statistically significant compared with the CON group (SMD = 0.26, 95% CI [-0.12, 0.64], I^2level-2^ = 27.02%, I^2level-3^ = 36.65%, PI [-0.96, 1.48], *p* = 0.18). Given the limited number of independent studies (n = 11) and the presence of dependent effect sizes, CRVE with the CR2 small-sample correction was applied, and the adjusted pooled estimate remained statistically non-significant (SMD = 0.26, 95% CI [-0.19, 0.71], *p* = 0.23). Overall, PT showed a small positive trend but did not significantly improve strength in this population. The forest plot is provided in **Appendix F**.

#### 3.4.2 Vertical jump performance

The effect of PT on vertical jump performance was evaluated using a three-level random-effects model (REML). Across 24 studies (44 effect sizes), PT significantly improved vertical jump performance compared with the CON group (SMD = 0.67, 95% CI [0.31, 1.03], I^2level-2^ = 10.28%, I^2level-3^ = 60.40%, PI [-0.88, 2.22], *p* < 0.001). Using CRVE with the CR2 small-sample correction, the effect remained statistically significant (SMD = 0.67, 95% CI [0.31, 1.04], *p* < 0.001), indicating that the estimated benefit of PT was robust to dependency-adjusted inference.

#### 3.4.3 Horizontal jump performance

The effect of PT on horizontal jump performance was evaluated using a three-level random-effects model (REML). Across 11 studies (16 effect sizes), PT significantly improved horizontal jump performance compared with the CON group (SMD = 0.70, 95% CI [0.41, 0.99], I^2level-2^ = 0.00%, I^2level-3^ = 11.91%, PI [0.24, 1.16], *p* < 0.001). Using CRVE with the CR2 small-sample correction, the effect remained statistically significant (SMD = 0.70, 95% CI [0.39, 1.01], *p* < 0.001), indicating that the estimated benefit of PT was robust to dependency-adjusted inference.

#### 3.4.4 Sprint performance

The effect of PT on sprint performance in female athletes was evaluated using a three-level random-effects model (REML). Across 15 studies (30 effect sizes), PT significantly improved sprint performance compared with the CON group (SMD = -0.85, 95% CI [-1.41, -0.29], I^2level-2^ = 0.37%, I^2level-3^ = 80.54%, PI [-2.90, 1.21], *p* = 0.004). Using CRVE with the CR2 small-sample correction, the effect remained statistically significant (SMD = -0.85, 95% CI [-1.43, -0.26], *p* = 0.008), indicating an improvement in sprint performance (i.e., reduced sprint time) that was robust to dependency-adjusted inference.

#### 3.4.5 Throwing performance

The effect of PT on throwing performance in female athletes was evaluated using a three-level random-effects model (REML). Across 5 studies (9 effect sizes), PT significantly improved throwing performance compared with the CON group (SMD = 0.91, 95% CI [0.29, 1.53], I^2level-2^ = 0.00%, I^2level-3^ = 32.83%, PI [-0.19, 2.01], *p* = 0.01). Using CRVE with the CR2 small-sample correction, the effect remained statistically significant (SMD = 0.91, 95% CI [0.16, 1.66], *p* = 0.03), indicating that the estimated benefit of PT was robust to dependency-adjusted inference.

#### 3.4.6 Agility performance

The effect of PT on agility performance in female athletes was evaluated using a three-level random-effects model (REML). Across 13 studies (22 effect sizes), PT significantly improved agility performance compared with the CON group (SMD = -1.09, 95% CI [-1.70, -0.48], I^2level-2^ = 4.58%, I^2level-3^ = 72.29%, PI [-3.16, 0.98], *p* = 0.001). Using CRVE with the CR2 small-sample correction, the effect remained statistically significant (SMD = -1.09, 95% CI [-1.73, -0.45], *p* = 0.003), indicating an improvement in agility performance (i.e., reduced completion time) that was robust to dependency-adjusted inference.

### 3.5 Secondary meta-analysis results

#### 3.5.1 Strength performance

Subgroup analysis revealed that the effects of PT on strength differed significantly across body parts (ΔSMD = 1.01, *p_b_* = 0.0025). Specifically, PT interventions targeting the upper-body yielded a substantial and significant improvement in upper-body strength (SMD = 1.09, 95% CI: 0.50 to 1.68, *p* < 0.001), with a high statistical power (96%). The heterogeneity for this subgroup was distributed as I^2level-2^ = 17.56% and I^2level-3^ = 58.64%. In contrast, PT protocols targeting the lower-body produced small and non-significant gains in lower-body strength (SMD = 0.08, 95% CI: -0.23 to 0.40, *p* = 0.601), with low statistical power (8%) and minimal within-study heterogeneity (I^2level-2^ = 0.00%).

Furthermore, neither age (*p_b_* = 0.710) nor training level (*p_b_* = 0.835) exhibited significant moderation effects. Regarding age, similar improvements were observed in both adolescent (SMD = 0.21, *p* = 0.446) and adult athletes (SMD = 0.37, *p* = 0.292). For training level, although a descriptive trend of increasing effect sizes was noted from Tier 2 (SMD = 0.13) to Tier 4 (SMD = 0.68), no significant differences were found between tiers. Notably, the statistical power for these non-significant subgroups was generally low (ranging from 6% to 19%). Regarding heterogeneity, except for Tier 3 (I^2level-2^ = 1.55%), the within-study variance (Level 2) for most non-significant subgroups was approximately 0.00%. The detailed subgroup analysis results are shown in **Table 2**.

**Table 2:** Subgroup analyses based on meta-analyses results of strength.

Subgroup	K(N)	Hedges’g	95% CI	T-value	P_d_	I^2level-2^	I^2level-3^	Power	P_b_
Age									0.7102
Adolescent	21 (171)	0.2067	[-0.3279, 1.0702]	0.7689	0.4455	0.00%	78.46%	12%	
Adults	32 (82)	0.3711	[-0.3331, 0.7466]	1.0658	0.2915	0.00%	29.09%	19%	
Level									0.8351
Tier 2	13 (88)	0.1309	[-0.6994, 0.9612]	0.3167	0.7528	0%	0%	6%	
Tier 3	38 (154)	0.2815	[-0.2683, 0.8313]	1.0283	0.3088	1.55%	76.26%	18%	
Tier 4	2 (11)	0.6755	[-0.9638, 2.3147]	0.8276	0.4118	0%	0%	13%	
Body part									0.0025
lower-body strength	47 (208)	0.0823	[-0.2314, 0.3960]	0.5269	0.6005	0.00%	26.74%	8%	
upper-body strength	6 (73)	1.0887	[0.5001, 1.6772]	3.7137	0.0005	17.56%	58.64%	96%	

Subgroup analyses based on meta-analyses results of Strength. K(N), number of effect size (sample size); Hedge’g, standardized mean difference; CI, confidence interval; P_d_, *p* value for subgroup differences; P_b_, *p* value between subgroups; I^2level-2^, heterogeneity within studies; I^2level-3^, heterogeneity between studies; Power, statistical power.

In parallel with subgroup analyses of categorical moderators, meta-regression was conducted to test whether continuous program characteristics were related to the magnitude of strength adaptations, considering both linear and non-linear functional forms. No significant linear or non-linear associations were identified for ground contacts per session (CONTACTS: (β = 0.004, linear *p* = 0.505; non-linear *p* = 0.481), weekly frequency (FRE: (β = 0.362, linear *p* = 0.406; non-linear *p* = 0.112), or intervention duration (WEEK: (β = 0.148, linear *p* = 0.343; non-linear *p* = 0.154). Collectively, anatomical site remains the primary moderator, while the examined continuous training variables did not significantly predict strength adaptations within the reported ranges.

#### 3.5.2 Agility performance

Subgroup analyses were conducted to explore potential moderators of the PT effect on agility performance. Training level significantly moderated the effects of PT on agility performance (*p_b_* = 0.0020). Athletes at Tier 3 demonstrated a large and statistically significant improvement (SMD = -2.37, 95% CI [-3.13, -1.60], *p* < 0.001), whereas the effects observed in Tier 2 (SMD = -0.53, *p* = 0.0736) and Tier 4 (SMD = -0.63, *p* = 0.1585) were not statistically significant. Substantial between-study heterogeneity was observed in the Tier 3 subgroup (I^2level-3^ = 82.36%).

In contrast, age did not significantly moderate the effect of PT (*p_b_* = 0.1868). PT significantly improved agility in adolescent athletes (SMD = -1.52, 95% CI [-2.40, -0.63], *p* = 0.0018), whereas the improvement observed in adult athletes did not reach statistical significance (SMD = -0.72, 95% CI [-1.56, 0.12], *p* = 0.0889). The heterogeneity among adolescent studies was substantial at the between-study level (I^2level-3^ = 87.92%), while no heterogeneity was observed among adult studies. The detailed subgroup analysis results are shown in **Table 3**.

**Table 3:** Subgroup analyses based on meta-analyses results of agility performance.

Subgroup	K(N)	Hedges’g	95%CI	T-value	P_d_	I^2^-2	I^2^-3	Power	P_b_
Age									0.1868
Adolescent	14 (136)	-1.5164	[-2.3983, -0.6346]	-3.5870	0.0018	0.00%	87.92%	95%	
Adults	8 (129)	-0.7189	[-1.5577, 0.1198]	-1.7880	0.0889	0.00%	0.00%	43%	
Level									0.0020
Tier 2	12 (103)	-0.5306	[-1.1169, 0.0558]	-1.8938	0.0736	0.00%	0.00%	47%	
Tier 3	7 (103)	-2.3654	[-3.1266, -1.6041]	-6.5036	<0.0001	0.00%	82.36%	99%	
Tier 4	3 (59)	-0.6268	[-1.5205, 0.2670]	-1.4678	0.1585	0.00%	6.17%	31%	

Subgroup analyses based on meta-analyses results of Agility. K(N), number of effect size (sample size); Hedge’g, standardized mean difference; CI, confidence interval; P_d_, *p* value for subgroup differences; P_b_, *p* value between subgroups; I^2level-2^, heterogeneity within studies; I^2level-3^, heterogeneity between studies; Power, statistical power.

Complementary to the subgroup analyses of categorical moderators, meta-regression analyses were performed to evaluate whether continuous programmatic variables were associated with the magnitude of PT-induced agility adaptations, using both linear and non-linear models. No significant linear or non-linear associations were identified for ground contacts per session (CONTACTS: (β = 0.0021, linear *p* = 0.398; non-linear *p* = 0.304), weekly frequency (FRE: (β = 0.288, linear *p* = 0.616; non-linear *p* = 0.374), or intervention duration (WEEK: (β = -0.0429, linear *p* = 0.746; non-linear *p* = 0.114). These results indicate that the examined continuous training variables were not significant predictors of agility adaptations within the reported ranges. Overall, training level was the only significant moderator identified for agility outcomes.

#### 3.5.3 Other performance outcomes

Subgroup analyses for vertical jump performance (VJP), horizontal jump performance (HJP), sprint performance (SP), and throwing performance (TP) did not reveal any significant moderating effects (all *p_b_* > 0.05). No statistically significant differences were observed between subgroup categories such as age or competitive level across these outcomes. Detailed subgroup statistics are presented in **Table 4**.

**Table 4:** Subgroup analyses based on meta-analyses results of other performance outcomes.

Variables	Subgroup	K(N)	Hedges’g	95%CI	T-value	P_d_	I^2^-2	I^2^-3	Power	P_b_
Vertical jump performance	Age									0.7143
	Adolescent	24 (233)	0.6163	[-0.0864, 1.3189]	1.7699	0.0840	2.19%	75.61%	42%	
	Adults	20 (317)	0.7771	[0.2466, 1.3075]	2.9564	0.0051	0.00%	79.50%	84%	
	Level									0.2451
	Tier 2	19 (148)	0.1656	[-0.6052, 0.9365]	0.4339	0.6666	0.00%	21.62%	7%	
	Tier 3	17 (305)	0.8860	[0.3235, 1.4486]	3.1809	0.0028	0.00%	69.03%	89%	
	Tier 4	8 (97)	1.0145	[0.1255, 1.9034]	2.3047	0.0263	0.00%	89.94%	63%	
	Jump type									0.5135
	CMJ	24 (506)	0.7367	[0.3087, 1.1648]	3.4760	0.0012	0.00%	73.79%	94%	
	DJ	8 (100)	0.8428	[0.2596, 1.4260]	2.9186	0.0057	3.50%	90.86%	83%	
	SJ	12 (267)	0.5487	[0.1048, 1.0646]	2.4608	0.0182	22.62%	41.55%	69%	
Horizontal jump performance	Age									0.3170
	Adolescent	6 (108)	0.8691	[0.4001, 1.3382]	3.9745	0.0014	0.00%	48.77%	98%	
	Adults	10 (138)	0.5731	[0.1802, 0.9661]	3.1281	0.0074	0.00%	0.00%	88%	
	Level									0.3955
	Tier 2	7 (118)	0.7672	[0.3230, 1.2115]	3.7313	0.0025	0.00%	40.59%	96%	
	Tier 3	7 (112)	0.7547	[0.2975, 1.2119]	3.5660	0.0034	0.00%	0.00%	95%	
	Tier 4	2 (16)	0.1036	[-0.8509, 1.0582]	0.2346	0.8182	0.00%	0.00%	6%	
Sprint performance	Age									0.3919
	Adolescent	12 (146)	-1.1899	[-2.1936, -0.1862]	-2.4284	0.0218	0.00%	94.32%	68%	
	Adults	18 (208)	-0.6663	[-1.3830, 0.0503]	-1.9046	0.0672	0.00%	62.59%	48%	
	Level									0.9734
	Tier 2	8 (120)	-0.7506	[-1.8407, 0.3395]	-1.4128	0.1691	0.00%	54.69%	29%	
	Tier 3	20 (218)	-0.9038	[-1.7105, -0.0970]	-2.2986	0.0295	0.00%	89.58%	63%	
	Tier 4	2 (16)	-0.8657	[-3.2828, 1.5515]	-0.7348	0.4688	0.00%	0.00%	11%	
Throwing performance	Age									0.2189
	Adolescent	3 (69)	1.1632	[0.3257, 2.0006]	3.2844	0.0134	24.48%	24.48%	91%	
	Adults	6 (49)	0.4915	[-0.3341, 1.3171]	1.4078	0.2020	0.00%	22.27%	29%	
	Level									0.5144
	Tier 2	2 (35)	1.0715	[-0.2468, 2.3898]	1.9888	0.0939	37.79%	37.79%	51%	
	Tier 3	2 (72)	1.0260	[-0.0794, 2.1314]	2.2712	0.0636	0.00%	0.00%	62%	
	Tier 4	5 (11)	0.2005	[-1.2685, 1.6696]	0.3340	0.7498	0.00%	0.00%	6%	

Subgroup analyses based on meta-analyses results of other performance outcomes. K(N), number of effect size (sample size); Hedge’g, standardized mean difference; CI, confidence interval; P_d_, *p* value for subgroup differences; P_b_, *p* value between subgroups; I^2level-2^, heterogeneity within studies; I^2level-3^, heterogeneity between studies; Power, statistical power.

To extend the moderator assessment beyond categorical subgrouping, meta-regression analyses examined whether continuous training variables exhibited linear or non-linear dose-response relationships with VJP, HJP, and SP. For VJP, no significant linear or non-linear associations were identified for ground contacts per session (CONTACTS: β = -0.0002, linear *p* = 0.953; non-linear *p* = 0.281), weekly frequency (FRE: β = 0.332, linear *p* = 0.386; non-linear *p* = 0.990), or intervention duration (WEEK: β = 0.124, linear *p* = 0.090; non-linear *p* = 0.177). For HJP, residual heterogeneity was non-significant (*p* > 0.60), and ground contacts per session (CONTACTS: β = 0.0001, linear *p* = 0.956; non-linear *p* = 0.317), FRE (β = 0.207, linear *p* = 0.281; non-linear *p* = 0.762), and WEEK (β = -0.073, linear *p* = 0.079; non-linear *p* = 0.848) showed no significant moderating effects. For SP, no significant linear or non-linear associations were observed for ground contacts per session (CONTACTS: β = 0.0026, linear *p* = 0.370; non-linear *p* = 0.924), FRE (β = -0.1133, linear *p* = 0.854; non-linear *p* = 0.443) and WEEK (β = -0.1484, linear *p* = 0.352; non-linear *p* = 0.262). Meta-regression was not conducted for throwing performance (TP) due to the limited number of available studies.

Overall, no meaningful subgroup differences or dose–response relationships were detected for VJP, HJP, and SP within the ranges of moderators available in the current evidence base. The detailed regression statistic are presented in **Supplementary Material**.

### 3.6 Sensitivity analysis

To evaluate the robustness of the pooled effect estimates, sensitivity analyses were performed using a leave-one-out approach, where each study was sequentially removed to assess its influence on the overall effect size and statistical significance. For the Strength, Vertical Jump Performance, Horizontal Jump Performance, Sprint Performance, Throwing Performance and Agility Performance outcomes, the leave-one-out results consistently demonstrated that the pooled estimates were highly robust. Excluding any single study did not materially change the direction of effects or the overall inference (significant vs non-significant) for each outcome. Specifically, the overall effect sizes for VJP, HJP, SP, TP and AP were significant, while Strength showed non-significant effects. To quantify this stability, the maximum observed change in the pooled SMD was 0.17 for Strength, 0.11 for VJP, 0.09 for HJP, 0.22 for SP, 0.19 for TP and 0.18 for AP. Thus, no single study significantly influenced the overall results. Detailed leave-one-out forest plots and sensitivity statistics for each outcome are provided in **Appendix G**.

Because the original studies did not report pre-post Pearson’s correlation coefficients, additional sensitivity analyses were conducted by varying the assumed correlation coefficient from r = 0.3 to r = 0.7, with r = 0.5 used in the primary analyses. For the majority of outcomes, changing the assumed correlation coefficient did not alter the direction or statistical significance of the pooled effects, and changes in effect size magnitude were small, indicating that the primary findings were generally robust to variation in the assumed correlation coefficient (**Table 5**). For horizontal jump performance, however, the model did not converge when r = 0.3 was specified; therefore, robustness under this specific assumption could not be fully evaluated.

**Table 5:** Sensitivity analysis for assumed pre-post Pearson’s correlation coefficients.

Variables	r	SMD	95% CI.lb	95% CI.ub	T-value	P
Strength	0.5	0.2591	-0.1204	0.6387	1.3701	0.1765
	0.3	0.2471	-0.1109	0.605	1.3851	0.1719
	0.7	0.3155	-0.1557	0.7867	1.3435	0.185
Vertical jump performance	0.5	0.6702	0.3146	1.0258	3.8009	0.0004
	0.3	0.5696	0.2681	0.8711	3.8102	0.0004
	0.7	0.8555	0.4002	1.3108	3.7893	0.0005
Horizontal jump performance	0.5	0.7002	0.4083	0.9921	5.1134	0.0001
	0.3	n/a	n/a	n/a	n/a	n/a
	0.7	0.8803	0.5283	1.2323	5.3309	< 0.0001
Sprint performance	0.5	-0.8482	-1.4081	-0.2883	-3.0981	0.0043
	0.3	-0.7263	-1.2121	-0.2405	-3.0576	0.0048
	0.7	-1.0648	-1.76	-0.3695	-3.1323	0.0039
Throwing performance	0.5	0.9088	0.2901	1.5274	3.3872	0.0095
	0.3	0.8009	0.3162	1.2857	3.81	0.0052
	0.7	1.1144	0.2713	1.9575	3.0482	0.0159
Agility performance	0.5	-1.0901	-1.7021	-0.4782	-3.7048	0.0013
	0.3	-0.9463	-1.474	-0.4185	-3.7286	0.0012
	0.7	-1.3431	-2.107	-0.5792	-3.6566	0.0015

Sensitivity Analysis for Assumed Pre-Post Pearson’s Correlation Coefficients. r, assumed pre-post Pearson’s correlation coefficient; SMD, standardized mean difference; 95% CI.lb, lower bound of the 95% confidence interval; 95% CI.ub, upper bound of the 95% confidence interval.

### 3.7 Results of the certainty of the evidence

Overall, the certainty of the evidence ranged from very low to moderate across outcomes (**Table 6**). Moderate-certainty evidence was found for horizontal jump and throwing performance, whereas the certainty of evidence for strength, vertical jump, sprint, and agility outcomes ranged from low to very low. Downgrading was primarily driven by risk of bias, with inconsistency and imprecision contributing to lower ratings for several outcomes.

**Table 6:** GRADE assessment results.

Outcome	No. of participants (Studies)	Certainty assessment						Effect size (SMD [95% CI])	Certainty (grade)
		Risk of Bias	Inconsistency	Indirectness	Imprecision	Publication bias	Other		
Strength	281 (11)	Serious	Serious	Not serious	Serious	Not serious	None	0.26 [-0.12, 0.64]	Very low
Vertical Jump Performance	558 (24)	Serious	Serious	Not serious	Not serious	Serious	None	0.67 [0.31, 1.03]	Very low
Horizontal Jump Performance	246 (11)	Serious	Not serious	Not serious	Not serious	Not serious	None	0.70 [0.41, 0.99]	Moderate
Sprint Performance	354 (15)	Serious	Serious	Not serious	Not serious	Serious	None	-0.85 [-1.41, -0.29]	Very low
Throwing Performance	118 (5)	Serious	Not serious	Not serious	Not serious	Not serious	None	0.91 [0.29, 1.53]	Moderate
Agility Performance	265 (13)	Serious	Serious	Not serious	Not serious	Not serious	None	-1.09 [-1.70, -0.48]	Low

The certainty of evidence according to the Grading of Recommendations, Assessment, Development, and Evaluations (GRADE) system is categorized into four levels: high, moderate, low, and very low. High certainty indicates a high level of confidence in the effect estimate; moderate certainty indicates a moderate level of confidence; low certainty indicates limited confidence; and very low certainty indicates very little confidence in the effect estimate. This classification helps assess the reliability of research findings and informs their interpretation and application.

## 4 Discussion

The present three-level meta-analysis indicates that PT is an effective strategy for improving several key performance outcomes in female team sport athletes, with clear benefits for jumping, sprinting, throwing, and agility. By contrast, strength adaptations appeared to be region-specific, with meaningful improvements observed mainly in the upper body. Agility responses also varied according to competitive level, whereas no consistent dose–response relationships were identified for training frequency, intervention duration, or ground contacts per session. Taken together, these findings suggest that PT-related adaptations in female team sport athletes are outcome-specific rather than uniform across performance domains. By applying a differentiated outcome framework and a three-level meta-analytic model that accounts for dependent effect sizes, this review extends recent evidence beyond broad physical fitness constructs. It also provides a more precise understanding of how PT influences distinct components of athletic performance in this population.

### 4.1 Strength

The present meta-analysis revealed a non-uniform pattern of strength adaptation across body regions. Upper-body strength showed a large and statistically significant improvement (SMD = 1.09), whereas lower-body strength demonstrated only a small and non-significant effect (SMD = 0.08). Taken together, these findings suggest that the effects of PT on strength in female team sport athletes may be region-specific rather than generalized.

One possible explanation for this pattern is that upper-body plyometric exercises may provide a relatively novel stimulus in female team sport athletes, particularly in sports where routine training and competition place greater mechanical emphasis on the lower limbs (Datson et al., 2014; Stojanović et al., 2018). In this context, the larger upper-body effect may reflect lower prior exposure to explosive upper-body loading and therefore greater scope for adaptation (Singla et al., 2018). This interpretation is broadly consistent with a recent systematic review and meta-analysis showing that upper-body PT can improve maximal strength in healthy youth and young adult participants (ES = 0.39). However, that review was not female-specific and explicitly identified the effects of upper-body PT in female participants as an important target for future research. Thus, the present findings may complement that evidence by suggesting that upper-body strength adaptations are also observable in female team sport athletes (Garcia-Carrillo et al., 2023). By contrast, the absence of a significant lower-body strength effect may be related to the already high exposure of the lower extremities to sport-specific stretch-shortening cycle actions, such as jumping, sprinting, and repeated accelerations, during regular training and competition (Rhea et al., 2003; Xu et al., 2025). As a result, additional lower-body PT may not consistently translate into measurable gains in maximal strength (Sáez-Sáez de Villarreal et al., 2010). Moreover, lower-body adaptations may be more readily reflected in explosive performance outcomes, such as jumping and sprinting, rather than in maximal strength measures. This may partly explain why lower-body maximal strength did not show a significant pooled effect despite the observed improvements in other lower-body-related performance outcomes. However, this interpretation should be made with caution, as the current evidence does not directly assess baseline strength levels, prior training exposure, the responsiveness of different outcome measure, or the mechanisms underlying regional differences in adaptation.

Subgroup and meta-regression analyses did not identify clear and consistent moderation by age, competitive level, training frequency, intervention duration, or ground contacts per session. These findings should not be interpreted as evidence that such factors are unimportant. Rather, they indicate that the currently available evidence did not detect clear and stable patterns of moderation for strength outcomes (Hedges and Pigott, 2004). Importantly, the statistical power for several non-significant subgroup comparisons was low (6-19%), suggesting that the available literature may be underpowered to detect modest moderator effects. Accordingly, strong conclusions regarding developmental stage, competitive level, or programming-related influences on strength adaptation are not yet warranted, and further well-powered studies are needed to clarify these relationships in female team sport athletes.

### 4.2 Explosive performance

#### 4.2.1 Jump performance

The present findings support the effectiveness of PT for improving both vertical (SMD = 0.67) and horizontal jump performance (SMD = 0.70) in female team sport athletes. Among the outcomes examined in this review, jump performance may be the most directly related to the characteristics of plyometric exercise, as both rely heavily on rapid force production and effective use of the stretch-shortening cycle. This close correspondence between training stimulus and performance outcome may partly explain why positive effects on jump performance were observed across multiple studies (Zhao et al., 2025).

A notable finding was that improvements in vertical jump performance were evident across several test modalities, including the CMJ, SJ, and DJ. Although no significant between-test subgroup differences were observed, the point estimates were numerically largest for the DJ, followed by the CMJ and SJ. This descriptive pattern may be consistent with the idea that PT transfers well to tasks involving reactive strength and rapid stretch-shortening cycle function. However, because the subgroup comparison was not statistically significant, this numerical ordering should not be interpreted as evidence of clear test-specific superiority. Rather, the current findings indicate that PT may enhance multiple components of vertical jump performance, including both concentric explosive output and reactive jump ability (Markovic, 2007).

This finding is also noteworthy because the present review observed beneficial effects across these vertical jump tasks, whereas a recent meta-analysis in a broader athletic population reported significant improvements in CMJ (SMD = 1.99, 95% CI [1.50, 2.48], *p* < 0.001) but not in SJ (SMD = 0.96, 95% CI [-0.10, 2.02], *p* = 0.07), indicating a relatively large point estimate accompanied by considerable uncertainty (Ma et al., 2025). This discrepancy may suggest that PT-related adaptations in specific jump tasks are influenced by differences in participant characteristics, sport background, outcome classification, and analytical approach. In the present review, the narrower focus on female team sport athletes may have provided a more population-specific estimate of jump-related adaptations.

Subgroup analyses did not identify age as a significant moderator for either vertical or horizontal jump performance, suggesting that the adaptive response of jump-related neuromuscular qualities may be relatively consistent across developmental stages. Likewise, the non-significant findings observed in some competitive-level subgroups should be interpreted cautiously. In Tier 2 athletes, these findings may reflect differences in physical preparedness or limited statistical power. In Tier 4 horizontal jump performance, the subgroup was based on only two primary studies despite including multiple effect sizes, which likely limited inferential precision. Accordingly, the current evidence does not support strong conclusions regarding competitive-level differences in jump adaptations.

Meta-regression analyses further indicated no significant dose-response relationships for training frequency, intervention duration, or ground contacts per session. Rather than suggesting that these programming variables are unimportant, this pattern suggests that jump adaptations can be achieved across a range of sessional loading structures represented in the current evidence base, provided that the plyometric stimulus is performed with sufficient intensity and movement quality (Chen et al., 2023). Nevertheless, these findings remain specific to the range of protocols included in the available studies.

#### 4.2.2 Sprint performance

The present meta-analysis also demonstrated that PT significantly improved sprint performance in female team sport athletes (SMD = -0.85), indicating faster sprint times following intervention. Compared with jump performance, the transfer of PT to sprinting may be explained more specifically by its contribution to rapid force production during high-speed locomotion (King et al., 2022). Sprint performance relies on the ability to generate large forces within very short ground-contact times, particularly during acceleration and the early stages of maximal-speed running (Weyand et al., 2000; Clark, 2022).

PT may improve sprint performance by enhancing reactive strength, rate of force development, and the efficiency of force application during the stance phase (Xu et al., 2025). These adaptations are likely relevant because sprinting requires efficient force expression under severe temporal constraints (Flanagan et al., 2025). Thus, although jumping and sprinting are both explosive tasks, the transfer of PT to sprint performance likely operates through improved force expression during brief contact periods rather than through jump capacity alone (Ramirez-Campillo et al., 2022).

Subgroup analyses did not identify age as a significant moderator, as both adolescent and adult athletes showed beneficial effects. Although Tier 3 athletes appeared to demonstrate larger improvements, subgroup-specific findings across competitive levels warrant cautious interpretation, particularly because statistical power was limited in some categories (Cuijpers et al., 2021). At present, the available evidence suggests that sprint-related benefits of PT may be observed across a broad range of female team sport athletes, but the extent to which these adaptations differ by competitive level remains uncertain (Lin et al., 2025; Zhao et al., 2025).

Meta-regression analyses did not detect significant associations between sprint performance gains and training frequency, intervention duration, or ground contacts per session. This may indicate that sprint-related adaptations are not determined solely by the accumulation of training volume, but also by the quality of explosive execution and the maintenance of high neuromuscular output during training (King et al., 2022). Given the time-constrained nature of sprinting, simply increasing training volume may not necessarily yield greater benefit and may, beyond a certain threshold, reduce movement quality through fatigue (Clark, 2022). Further studies using a wider range of sprint-specific plyometric protocols are needed to clarify potential dose-response relationships.

#### 4.2.3 Throwing performance

A large and statistically significant improvement was also observed in throwing performance following PT interventions (SMD = 0.91). This finding highlights the potential value of upper body plyometric exercises, such as medicine ball throws and explosive push-ups, for enhancing throwing-related power in female team sport athletes (Singla et al., 2018).

Unlike traditional resistance training, plyometric exercises emphasize rapid force production during the stretch-shortening cycle, which may enhance the rate of force development, intermuscular coordination, and the efficient transfer of force through the kinetic chain during throwing actions (Garcia-Carrillo et al., 2023; Job et al., 2024). In this context, improvements in throwing performance may reflect not only greater upper-limb power, but also better integration of trunk and upper-extremity movements during explosive sport-specific actions (Jones et al., 2024).

The substantial improvement observed in the present analysis is also broadly consistent with the gains in upper-body strength identified in the strength subgroup analysis, suggesting that female team sport athletes may respond favorably to explosive upper-body training stimuli (Garcia-Carrillo et al., 2023). This pattern may be particularly relevant in this population, in whom upper-body loading during routine sport participation is often less pronounced than lower-body loading, thereby leaving greater scope for adaptation (Trasolini et al., 2022).

Subgroup analyses indicated that improvements in throwing performance were generally consistent across age groups and competitive levels. However, these analyses were based on a limited number of studies (k = 5), which restrict statistical power and reduce confidence in subgroup-specific inferences (Hedges and Pigott, 2004). Therefore, although the overall effect appears promising, further well-controlled studies are needed to confirm the generalizability of these findings across different female team sport populations.

### 4.3 Agility performance

The present meta-analysis demonstrated that PT significantly improved agility performance in female team sport athletes (SMD = -1.09). Compared with jumping and sprinting, agility is a more complex performance domain, requiring not only explosive force production but also rapid deceleration, body repositioning, and re-acceleration (Chaabene et al., 2018). Accordingly, the effects of PT on agility may reflect a broader integration of physical and movement-control capacities (Carvajal-Espinoza et al., 2025).

PT may enhance agility by improving reactive strength, eccentric force control, and the ability to absorb and re-apply force during directional changes (Singh et al., 2025; Harper et al., 2026). These qualities are particularly relevant to change-of-direction tasks, in which successful performance depends on controlling momentum before rapidly producing force in a new direction (Nygaard Falch et al., 2019). Thus, improvements in agility may arise not only from greater explosiveness, but also from more efficient transitions between braking and propulsion phases (Singh et al., 2025).

Unlike the findings for jump and sprint outcomes, subgroup analyses indicated that competitive level may moderate agility adaptations, with Tier 3 athletes showing comparatively larger improvements. One possible explanation is that agility performance may depend more strongly on the interaction between physical capacity and movement proficiency than other explosive outcomes (Carvajal-Espinoza et al., 2025). Athletes at an intermediate-to-advanced competitive level may therefore possess sufficient physical and coordinative capacity to benefit from plyometric loading while still retaining room for further improvement. However, this interpretation remains tentative, and these subgroup findings should be viewed cautiously because some categories were based on relatively small samples.

Meta-regression analyses did not detect significant associations between agility improvements and training frequency, intervention duration, or ground contacts per session. This should not be interpreted as evidence that programming variables are unimportant; rather, it indicates that the currently available evidence did not reveal clear or consistent dose-response patterns for agility outcomes (Hedges and Pigott, 2004). Given the multidimensional nature of agility, future studies should examine whether combining PT with more task-specific change-of-direction drills produces greater or more consistent benefits in female team sport athletes.

### 4.4 Practical applications

The findings of this meta-analysis support the inclusion of PT in the physical preparation programs of female team sport athletes. As no clear dose-response relationships were identified, practitioners should not assume that simply increasing training volume will produce greater performance gains. Likewise, the current evidence does not support a single optimal prescription in terms of training frequency, intervention duration, or ground contacts per session. Instead, emphasis should be placed on movement quality, maximal intent, and explosive execution in each repetition. In practice, PT may be more appropriately performed under relatively low-fatigue conditions, with volume adjusted when jump quality, landing mechanics, or movement velocity begin to deteriorate. Low-to-moderate-volume plyometric sessions may be particularly useful during congested training and competition periods, as they may provide sufficient neuromuscular stimulus while minimizing unnecessary fatigue. Rather than emphasizing a high number of contacts, coaches may benefit from prescribing smaller volumes of well-executed efforts that can be integrated efficiently into the weekly training schedule.

In addition, the substantial improvements observed in upper-body strength and throwing performance highlight the value of incorporating upper-body plyometric exercises alongside lower-body drills. Exercises such as medicine ball throws and explosive push-ups may be especially relevant in sports that require rapid passing, throwing, or other explosive upper-limb actions. Accordingly, practitioners may benefit from designing plyometric programs that reflect both the sport-specific movement demands and the athlete’s current training status, while balancing upper- and lower-body explosive training within the overall program.

### 4.5 Limitations

Several limitations should be considered when interpreting the findings of the present study. First, although the overall meta-analytic results were generally robust, several subgroup analyses were characterized by limited statistical power because only a small number of studies were available within certain categories. Consequently, some non-significant subgroup findings may reflect insufficient evidence rather than a true absence of training effects. Second, the available literature on upper-body PT in female team sport athletes remains limited. Only a small number of studies examined throwing performance, which restricted the possibility of conducting more detailed meta-regression analyses for upper-body training variables. Therefore, conclusions regarding upper-body-specific adaptations should be interpreted with caution until confirmed by a larger body of evidence. Third, the meta-regression analyses were restricted to the range of training protocols reported in the included studies. It is therefore possible that different dose-response relationships may emerge under training conditions outside these observed ranges. Fourth, variation in participant characteristics, sport-specific demands, testing procedures, and plyometric program design across studies may also have contributed to heterogeneity in the pooled estimates. In addition, the inclusion of both randomized and non-randomized controlled studies may have increased methodological heterogeneity in the evidence base. Finally, the certainty of evidence ranged from very low to moderate across outcomes, indicating that confidence in some pooled estimates remains limited despite the overall robustness of the findings. Future randomized controlled trials with larger samples and more standardized reporting of PT variables are needed to clarify the optimal programming parameters for female team sport athletes, particularly for upper-body outcomes and competitive-level subgroups.

## 5 Conclusion

This systematic review and three-level meta-analysis suggests that PT is a useful strategy for improving several key performance outcomes in female team sport athletes. Beneficial effects were observed for jumping, sprinting, agility, and throwing performance, whereas strength-related adaptations appeared to be less consistent and more region-specific, with meaningful improvements observed primarily in the upper body. Overall, these findings indicate that PT-related adaptations in female team sport athletes are outcome-specific rather than uniform across performance domains. Subgroup and meta-regression analyses did not identify clear and consistent moderation across most outcomes, suggesting that PT-induced adaptations may be achievable across a range of programming structures represented in the current evidence base, although such interpretations should remain limited to the protocols and populations included in the available literature.

In conclusion, structured PT can be considered a practical and adaptable approach for enhancing explosive performance in female team sport athletes. Nevertheless, the certainty of evidence varied across outcomes, and further research with larger and more diverse samples is needed to confirm these findings and clarify the training parameters most strongly associated with specific performance adaptations.

## Data Availability

All data analyzed in this systematic review were obtained from publicly available databases. As the data were collected from multiple sources, no single accession number is applicable. The search strategies for all databases are provided in the **Appendix B**, allowing independent retrieval of the data. Additionally, the integrated dataset supporting the results of this study can be obtained from the corresponding author upon request.

## Supplementary material

Supplementary Table 1 Non-linear meta-regression results.

Supplementary Figure 1 Linear meta-regression plots. Column a represents the contacts; Column b represents the frequency; Column c represents the week. Row 1 represents the strength; row 2 represents the vertical jump performance; row 3 represents the horizontal jump performance; row 4 represents the sprint performance; row 5 represents the agility performance.
