# Supplementary material for: Effects of plyometric training on strength, explosive performance, and agility in female team-sport athletes: a systematic review and three-level meta-analysis
Source: Front Physiol. 2026 May 5;17:1837615. doi: 10.3389/fphys.2026.1837615 (PMC13183815; doi:10.3389/fphys.2026.1837615)
Supplement: Supplementary Table 1 — Non-linear meta-regression results. [file SupplementaryFile1.docx]

**Appendix A**

**Table A1.** PRISMA Checklist 2020

| **Section and Topic** | **Item** | **Checklist Item** | **Location Where Item Is Reported** |
| --- | --- | --- | --- |
| **Title** | | |  |
| Title | 1 | Identify the report as a systematic review. | Title page |
| **Abstract** | | |  |
| Abstract | 2 | See the PRISMA 2020 for Abstracts checklist. | Abstract |
| **Introduction** | | |  |
| Rationale | 3 | Describe the rationale for the review in the context of existing knowledge. | 1 Introduction |
| Objectives | 4 | Provide an explicit statement of the objective(s) or question(s) the review addresses. | 1 Introduction |
| **Methods** | | |  |
| Eligibility criteria | 5 | Specify the inclusion and exclusion criteria for the review and how studies were grouped for the syntheses. | 2.3 Eligibility criteria |
| Information sources | 6 | Specify all databases, registers, websites, organizations, reference lists and other sources searched or consulted to identify studies. Specify the date when each source was last searched or consulted. | 2.1 Information Sources and Search Strategy |
| Search strategy | 7 | Present the full search strategies for all databases, registers and websites, including any filters and limits used. | 2.1 Information Sources and Search Strategy  Appendix B |
| Selection process | 8 | Specify the methods used to decide whether a study met the inclusion criteria of the review, including how many reviewers screened each record and each report retrieved, whether they worked independently, and if applicable, details of automation tools used in the process. | 2.2 Selection Process |
| Data collection process | 9 | Specify the methods used to collect data from reports, including how many reviewers collected data from each report, whether they worked independently, any processes for obtaining or confirming data from study investigators, and if applicable, details of automation tools used in the process. | 2.1 Information Sources and Search Strategy |
| Data items | 10a | List and define all outcomes for which data were sought. Specify whether all results that were compatible with each outcome domain in each study were sought (e.g., for all measures, timepoints, analyses), and if not, the methods used to decide which results to collect. | 2.4 Data Extraction |
|  | 10b | List and define all other variables for which data were sought (e.g., participant and intervention characteristics, funding sources). Describe any assumptions made about any missing or unclear information. | 2.4 Data Extraction |
| Study risk of bias assessment | 11 | Specify the methods used to assess risk of bias in the included studies, including details of the tool(s) used, how many reviewers assessed each study and whether they worked independently, and if applicable, details of automation tools used in the process. | 2.6 Risk of Bias and Quality of Methods Assessment |
| Effect measures | 12 | Specify for each outcome the effect measure(s) (e.g., risk ratio, mean difference) used in the synthesis or presentation of results. | 2.7.2 Effect Size and Heterogeneity Assessment |
| Synthesis methods | 13a | Describe the processes used to decide which studies were eligible for each synthesis (e.g., tabulating the study intervention characteristics and comparing against the planned groups for each synthesis (item #5)). | 2.3 Eligibility Criteria |
|  | 13b | Describe any methods required to prepare the data for presentation or synthesis, such as handling of missing summary statistics, or data conversions. | 2.5 Data Processing |
|  | 13c | Describe any methods used to tabulate or visually display results of individual studies and syntheses. | 2.7 Statistical Analysis |
|  | 13d | Describe any methods used to synthesize results and provide a rationale for the choice(s). If meta-analysis was performed, describe the model(s), method(s) to identify the presence and extent of statistical heterogeneity, and software package(s) used. | 2.7.2 Effect Size and Heterogeneity Assessment |
|  | 13e | Describe any methods used to explore possible causes of heterogeneity among study results (e.g., subgroup analysis, meta-regression). | 2.7.3 Moderator and Sensitivity Analyses |
|  | 13f | Describe any sensitivity analyses conducted to assess robustness of the synthesized results. | 2.7.3 Moderator and Sensitivity Analyses |
| Reporting bias assessment | 14 | Describe any methods used to assess risk of bias due to missing results in a synthesis (arising from reporting biases). | 2.6 Risk of Bias and Quality of Methods Assessment |
| Certainty assessment | 15 | Describe any methods used to assess certainty (or confidence) in the body of evidence for an outcome. | 2.8 Certainty of the Evidence |
| **Results** | | |  |
| Study selection | 16a | Describe the results of the search and selection process, from the number of records identified in the search to the number of studies included in the review, ideally using a flow diagram. | 3.1 Study Selection |
|  | 16b | Cite studies that might appear to meet the inclusion criteria, but which were excluded, and explain why they were excluded. | n/a |
| Study characteristics | 17 | Cite each included study and present its characteristics. | 3.1 Study Selection |
| Risk of bias in studies | 18 | Present assessments of risk of bias for each included study. | 3.3 Risk of Bias Aessment, Figure 2a-2d |
| Results of individual studies | 19 | For all outcomes, present, for each study: (a) summary statistics for each group (where appropriate) and (b) an effect estimate and its precision (e.g., confidence/credible interval), ideally using structured tables or plots. | 3.4 Primary Meta-Analysis Results  3.5 Secondary Meta-Analysis Results |
| Results of syntheses | 20a | For each synthesis, briefly summarize the characteristics and risk of bias among contributing studies. | 3.4 Primary Meta-Analysis Results  3.5 Secondary Meta-Analysis Results |
|  | 20b | Present results of all statistical syntheses conducted. If meta-analysis was performed, present for each the summary estimate and its precision (e.g., confidence/credible interval) and measures of statistical heterogeneity. If comparing groups, describe the direction of the effect. | 3.4 Primary Meta-Analysis Results  3.5 Secondary Meta-Analysis Results |
|  | 20c | Present results of all investigations of possible causes of heterogeneity among study results. | 3.4 Primary Meta-Analysis Results  3.5 Secondary Meta-Analysis Results |
|  | 20d | Present results of all sensitivity analyses conducted to assess the robustness of the synthesized results. | 3.6 Sensitivity Analysis  Table 5  Appendix G |
| Reporting biases | 21 | Present assessments of risk of bias due to missing results (arising from reporting biases) for each synthesis assessed. | 3.3 Risk of Bias Aessment, Figure 2a-2d |
| Certainty of evidence | 22 | Present assessments of certainty (or confidence) in the body of evidence for each outcome assessed. | 3.7 Results of Certainty of the Evidence |
| **Discussion** | | |  |
| Discussion | 23a | Provide a general interpretation of the results in the context of other evidence. | 4 Discussion |
|  | 23b | Discuss any limitations of the evidence included in the review. | 4.5 Limitations |
|  | 23c | Discuss any limitations of the review processes used. | 4.5 Limitations |
|  | 23d | Discuss implications of the results for practice, policy, and future research. | 4.4 Practical Applications |
| **Other Information** | | |  |
| Registration and protocol | 24a | Provide registration information for the review, including register name and registration number, or state that the review was not registered. | 2 Methods |
|  | 24b | Indicate where the review protocol can be accessed, or state that a protocol was not prepared. | n/a |
|  | 24c | Describe and explain any amendments to information provided at registration or in the protocol. | n/a |
| Support | 25 | Describe sources of financial or non-financial support for the review, and the role of the funders or sponsors in the review. | Title page |
| Competing interests | 26 | Declare any competing interests of review authors. | Title page |
| Availability of data, code and other materials | 27 | Report which of the following are publicly available and where they can be found: template data collection forms; data extracted from included studies; data used for all analyses; analytic code; any other materials used in the review. | n/a |

**Note:** n/a, not available.

**Appendix B**

**Table B1.** The detailed search strategy

| **Data** | **Query** | **Results in 2025/04/30** | **Results in 2026/03/10** |
| --- | --- | --- | --- |
| **PubMed** | (((plyometrics) OR(plyometric exercise) OR(plyometric training) OR(plyometric drill) OR(stretch shorten cycle exercise) OR(stretch shorten cycle training) OR(stretch shorten cycle drill) OR(jump training) OR(jump exercise) OR(countermovement jump) OR(drop jump) OR(squat jump) OR(hurdle jump) OR(tack jump) OR(box jump training) OR(vertical jump) OR(standing long jump) OR(ballistic training) OR(explosive training)) AND ((women) OR(woman) OR(girl) OR(girls) OR(women's group) OR(female) OR(feminine))) AND(randomized controlled trial) | 958 | 1081 |
| **Web of science** | (((TS=plyometrics) OR(TS=plyometric exercise) OR(TS=plyometric training) OR(TS=plyometric drill) OR(TS=stretch shorten cycle exercise) OR(TS=stretch shorten cycle training) OR(TS=stretch shorten cycle drill) OR(TS=jump training) OR(TS=jump exercise) OR(TS=countermovement jump) OR(TS=drop jump) OR(TS=squat jump) OR(TS=hurdle jump) OR(TS=tack jump) OR(TS=box jump training) OR(TS=vertical jump) OR(TS=standing long jump) OR(TS=ballistic training) OR(TS=explosive training)) AND ((TS=women) OR(TS=woman) OR(TS=girl) OR(TS=girls) OR(TS=women's group) OR(TS=female) OR(TS=feminine))) AND(TS=randomized controlled trial) | 746 | 867 |
| **Cochrane library** | #1 (plyometric OR "plyometric exercise" OR "plyometric training" OR "plyometric drill"):ti,ab,kw  #2 ("stretch-shortening cycle exercise" OR "stretch-shortening cycle training" OR "stretch-shortening cycle drill" OR "jump training" OR "jump exercise"):ti,ab,kw  #3 ("countermovement jump" OR "drop jump" OR "squat jump" OR "hurdle jump" OR "tuck jump"):ti,ab,kw  #4 ("box jump training" OR "vertical jump" OR "standing long jump" OR "ballistic training" OR "explosive training"):ti,ab,kw  #5 (women OR woman OR girl OR female OR "women's group"):ti,ab,kw  #6 ("randomized controlled trial" OR randomized OR randomised):ti,ab,kw  #7 (#1 OR #2 OR #3 OR #4) AND #5 AND #6 | 796 | 829 |
| **Ebsco - Medline with full text** | SU (plyometrics or plyometric exercise or plyometric training or plyometric drill or stretch shorten cycle exercise or stretch shorten cycle training or stretch shorten cycle drill or jump training or jump exercise or countermovement jump or drop jump or squat jump or hurdle jump or tack jump or box jump training or vertical jump or standing long jump or ballistic training or explosive training) AND SU (women or woman or girl or girls or women's group or female) | 569 | 681 |
| **Ebsco - Sportdiscus with full text** | SU (plyometrics or plyometric exercise or plyometric training or plyometric drill or stretch shorten cycle exercise or stretch shorten cycle training or stretch shorten cycle drill or jump training or jump exercise or countermovement jump or drop jump or squat jump or hurdle jump or tack jump or box jump training or vertical jump or standing long jump or ballistic training or explosive training) AND SU (women or woman or girl or girls or women's group or female) | 281 | 328 |
| **Embase** | #1: plyometrics:ti,ab,kw OR 'plyometric exercise':ti,ab,kw OR 'plyometric training':ti,ab,kw OR 'plyometric drill':ti,ab,kw OR 'stretch shorten cycle exercise':ti,ab,kw OR 'stretch shorten cycle training':ti,ab,kw OR 'stretch shorten cycle drill':ti,ab,kw OR 'jump training':ti,ab,kw OR 'jump exercise':ti,ab,kw OR 'countermovement jump':ti,ab,kw OR 'drop jump':ti,ab,kw OR 'squat jump':ti,ab,kw OR 'hurdle jump':ti,ab,kw OR 'tack jump':ti,ab,kw OR 'box jump training':ti,ab,kw OR 'vertical jump':ti,ab,kw OR 'standing long jump':ti,ab,kw OR 'ballistic training':ti,ab,kw OR 'explosive training':ti,ab,kw  #2: women:ti,ab,kw OR woman:ti,ab,kw OR girl:ti,ab,kw OR 'womens group':ti,ab,kw OR female:ti,ab,kw  #3：'randomized controlled trial'  #4: #1 AND #2 AND #3 | 353 | 452 |
| **CNKI** | SU=('快速伸缩复合训练' +'增强式训练' +'超等长训练' +'拉长缩短周期' +'跳跃训练' +'爆发力训练' +'跳箱训练' +'末端释放训练' +'反向纵跳练习' +'下落跳练习' +'蹲跳练习' +'跨栏跳练习' +'团身跳练习' +'立定跳练习') and SU=('女性' +'女性运动员') | 14 | 19 |
| **Wan Fang** | 题名或关键词:((快速伸缩复合训练) OR(增强式训练) OR(超等长训练) OR(拉长缩短周期) OR(跳跃训练) OR(爆发力训练) OR(跳箱训练) OR(末端释放训练) OR(反向纵跳练习) OR(下落跳练习) OR(蹲跳练习) OR(跨栏跳练习) OR(团身跳练习) OR(立定跳练习)) AND 题名或关键词:((女性) OR(女性运动员)) | 20 | 22 |
| **VIP** | ((M=快速伸缩复合训练 OR M=增强式训练 OR M=超等长训练 OR M=拉长缩短周期 OR M=跳跃训练 OR M=爆发力训练 OR M=跳箱训练 OR M=末端释放训练 OR M=反向纵跳训练 OR M=下落跳练习 OR M=蹲跳练习 OR M=跨栏跳练习 OR M=团身跳练习 OR M=立定跳练习)) AND ((M=女性 OR M=女性运动员)) AND (M=随机对照实验) | 145 | 152 |

**Abbreviations:** ***CNKI*** = Chinese National Knowledge Infrastructure; ***VIP*** = Chinese Scientific Journals Database; ***ti*** = title; ***ab*** = abstract; ***kw*** = keywords; ***TS*** = topic; ***SU*** = subject terms; ***M*** = MeSH term

**Appendix C**

**Table C1.** Included studies characteristics

| **Study** | **Design** | **Participants** | **PT sessions** | **CG sessions** | **PT (fre × wk)** |
| --- | --- | --- | --- | --- | --- |
| Mack, 2011 | NRCT | PT - N: 10, CG - N: 6  PT - Age: 13.4, CG - Age: 13.4  Adolescent soccer players  Train status: Tier 2 Trained/Developmental  Training experience: ≥ 4 years | PT exercises (intensity): single - leg cone hops (6 inches), forward hurdle hops (10 inches), lateral hurdle hops (10 inches), box shuffles over a single box (12 inches), box jump ups (10 inches), box depth jumps (10 inches), cutting drills around cones (5 yards apart with 5 yards stagger) | Soccer training 3 times a week and soccer match 2 - 3 times a week | Lower limb (1 × 14) |
| Alberto, 2021 | RCT | PT - N: 11, CG - N: 12  PT - Age: 23.0, CG - Age: 22.58  Basketball players  Train status: Tier 2 Trained/Developmental  Training experience: at least 5 years | PT exercises (intensity): drop jump (20, 30 and 40cm), repeat jumps (body weight-as high as possible)  Recovery: 1 minute between each exercise | Basketball training three times a week, each session lasting 90 minutes, and one match per week. | Lower limb (2 × 6) |
| David, 2018 | NRCT | PT - N: 7, CG - N: 4  PT - Age: 21.86, CG - Age: 27.25  Volleyball players of professional team in Spanish League A  Training status: Tier 4 Elite/International Level  Training experience: at least 5 years | PT exercises (intensity): prone medicine ball overhead Throw (3 kg), prone overhead throw with medicine ball (3 kg)  Recovery: 3 minutes between each exercise | During the intervention period, the control group performed technical-tactical drills including spiking and blocking. | Upper limb (2 × 8) |
| Gary, 2004 | NRCT | PT - N: 11, CG - N: 8  PT - Age: 19.0, CG - Age: 19.0  PT: NCAA Division 2 basketball players  CG: NCAA Division 1 basketball players  Training status: Tier 4 Elite/International Level (CG); Tier 3 Highly Trained/National Level (PT) | PT exercises (intensity): wall jumps, tuck jumps, broad jumps stick hand, squat jumps, double leg cone jumps, 180° jumps, bounding in place, jump-jump-jump-vert jump, bounding for distance, scissor jump, hop-hop-stick, step-jump up-jump down-vertical, mattress jump, single-legged jumps distance, jump into bounding, single-legged hop-hop-stick  Recovery: 0.5 minutes between each exercise | Routine training | Lower limb (3 × 6) |
| Rodrigo, 2018 | RCT | PT1 - N: 8, PT2 - N: 8, CG - N: 7  PT1 - Age: 22.8, PT2 - Age: 21.4,  CG - Age: 20.1  Train status: Tier 2 Trained/Developmental  Training experience: 5 years | PT exercises: drop jump, standing long jump, unilateral counter movement jump, 180° jump, repeated counter movement jump.  Recovery: 0.5 - 1 minutes between exercises. | Regular soccer training | Lower limb (PT1: 1 × 8; PT2: 2 × 8) |
| Gustavo, 2020 | RCT | PT (Mat2) - N: 10, PT (Mat3) - N: 10  PT (Mat4) - N: 10, CG (Mat2) - N: 11  CG (Mat3) - N: 19, CG (Mat4) - N: 19  PT (Mat2) - Age: 10.5, PT (Mat3) - Age: 11.8,  PT (Mat4) - Age: 13.2, CG (Mat2) - Age: 11.7,  CG (Mat3) - Age: 12.3, CG (Mat4) - Age: 13.0  Children volleyball players  Train status: Tier 2 Trained/Developmental  Training experience: at least 6 months | PT exercises (intensity): broad jumps, alternate leg bounds (W1: 60 cm; W2: 80 cm; W3 - W6:100 cm; W7:120 cm; W8:100 cm), zigzag hops (W1, W4: 50 cm; W2: 70 cm; W3: 90 cm; W5 - W6: 100 cm; W7 - W8: 120 cm), hurdle jumps (W4: 20 cm; W5: 30 cm; W6 - W7: 40 cm; W8: 60 cm), box jumps (W4 - W5: 20 cm; W6 - W7: 40 cm; W8: 60 cm)  Recovery: 1 - 2 minutes between each exercise | Regular volleyball training 3 times a week | Lower limb (3 × 8) |
| Jason, 2001 | NRCT | PT - N: 17, CG - N: 17  PT - Age: 16.49, CG - Age: 16.27  High school soccer players  Train status: Tier 2 Trained/Developmental | PT exercises (intensity): box jumps, timed jumps, repeated jumps | Endurance training 2 - 3 times a week | Lower limb (3 × 10) |
| Elif, 2019 | RCT | PT - N: 14, CG - N: 14  PT - Age: 11.0, CG - Age: 11.0  Volleyball players  Training status: Tier 2 Trained /Developmental  Training experience: 2-4 years | PT exercises (intensity): shoulder external rotation with resistance band at 0° and 90° abduction, overhead medicine ball throw (2 kg), Lateral medicine ball throw at 90° abduction, Decelerated throwing, Volleyball serve  Recovery: 0.5 - 0.67 minutes between each exercise | Regular volleyball training | Upper limb (3 × 12) |
| Hammami, 2020 | RCT | PT - N: 17, CG - N: 17  PT - Age: 17.0, CG - Age: 17.0  Handball players  Training status:Tier 3 Highly Trained / National Level  Training experience: 5 years | PT exercises (intensity): push-up (body weight); W1 - W4: hurdle jump (30 cm height), lateral hurdle jump (30 cm height), stiff-leg jump (25 cm height), rotational hurdle jump (25 cm height)  W5 - W8: first four exercises ↑ 5 cm (to 35 cm height) standing long jump ↑ 10 cm (to 120 cm distance), standing long jump (110 cm distance)  W9 - W10: first four exercises ↑5 cm (to 40 cm height), standing long jump ↑ 10 cm (to 130 cm distance)  Recovery: 0.5 minutes between each exercise | Regular handball training | Upper and lower limb (2 × 10) |
| Neves, 2017 | RCT | PT - N: 10, CG - N: 10  PT - Age: 19.4, CG - Age: 19.5  Futsal players  Train status: Tier 2 Trained/Developmental  Training experience: at least 3 years | PT exercises (intensity): alternate-leg jump, single-leg squat jump, explosive repeated jump, counter movement jump (CMJ), repeated horizontal jump, tuck jump, progressive explosive jump series, rhythm jump, 30 seconds jump rope, diagonal obstacle jump, repeated tuck jump  Recovery: 1.5 minutes between each exercise | Regular technical and tactical training | Lower limb (2 × 4) |
| Nicole, 2004 | RCT | PT - N: 9, CG - N: 9  PT - Age: 20.0, CG - Age: 20.0  NCAA Division 1 soccer and hockey players  Training status: Tier 4 Elite/International Level | PT exercises (intensity): wall touch jump, split squat jump, lateral cone hop, 180° cone rotation jump, drop jump  Recovery: 0.5 minutes between sets, 2 minutes between each exercise | All subjects participated in regularly scheduled off-season strength training, practices, and games and tournaments | Lower limb (2 × 6) |
| Gulcan, 2016 | RCT | PT - N: 17, CG - N: 17  PT - Age: 15.5, CG - Age: 15.6  Volleyball players  Train status: Tier 2 Trained/Developmental  Training experience: at least 4 years | PT exercises (intensity): wall jumps, tuck jumps, broad jumps stick hand, squat jumps, double leg cone jumps, 180° jumps, bounding in place, jump-jump-jump-vert jump, bounding for distance, scissor jump, hop-hop-stick, step-jump up-jump down-vertical, mattress jumps, single-legged jumps distance, jump into bounding, single-legged hop-hop-stick  Recovery: 0.5 minutes between each exercise | Con group including resistive upper and lower body exercises, and core stability (2 days in a week), aerobic exercises (4 days in a week) and volleyball technical training (3 days in a week) | Lower limb (3 × 6) |
| Fabian, 2017 | RCT | PT - N: 8, CG - N: 9  PT - Age: 22.8, CG - Age: 24.0  Soccer players  PT training experience: average 7.5 years  CG training experience: average 9.1 years  Train status : Tier 2 Trained/Developmental | PT exercises (intensity): cmj with arm swing: cyclic horizontal left/right leg, acyclic horizontal left/right leg, cyclic vertical right/left leg, acyclic vertical left/right leg, cyclic bilateral vertical/horizontal, acyclic bilateral vertical/horizontal, bounce drop jumps 40 cm  Recovery: 2 minutes between each exercise | Con group participated in the same soccer training program, yielding similar training loads measured via the session rating of perceived exertion | Lower limb (2 × 6) |
| Wang, 2024 | RCT | PT - N: 13, CG - N: 13  PT - Age: 20.92, CG - Age: 20.31  Soccer players  Training status:Tier 3 Highly Trained / National Level  Training experience: at least 7 years | PT exercises (intensity): half squat jump, squat jump, scissor jump, marching high knees, straight leg run with single leg high knee, knee tuck jump  Recovery: 1-5 min between exercises | Weighted squat and half squat, bent leg dead lift, straight leg dead lift, weighted lunges, weighted calf raises, frog jumps, quick squat jumps (body weight/65-80% 1rm)  Recovery: 1 - 1.5 min between exercises | Lower limb (3 × 16) |
| Wang, 2022 | RCT | PT - N: 9, CG - N: 9  PT - Age: 19.89, CG - Age: 20.0  Basketball players  Train status : Tier 2 Trained/Developmental  Training experience: at least 3 years | PT exercises (intensity): rocket jumps, split jumps, skip with high knees, quick skip jumps, single leg step ups, lateral hopscotch, alternating leg step ups, single leg high knees, progressive double leg bounds, lateral bounds with sprint, progressive box jumps  Recovery: 0.5 - 3 minutes between each exercise | Weighted back squat, weighted walking lunges, weighted back squat, weighted calf raises, bent leg dead lift, weighted bulgarian split squat, resistance band sprints (body weight/10%-75% 1 RM)  Recovery: 1.5 - 3 min between exercises | Lower limb (2 × 12) |
| Wang, 2019 | RCT | PT - N: 10, CG - N: 10  PT - Age: 19.7, CG - Age: 19.4  Volleyball players  Training status:Tier 3 Highly Trained / National Level  Training experience: at least 6 years | PT exercises (intensity): lunge jumps, depth jumps, sit-ups, medicine ball sit-up pass, continuous block jumps, lateral box jumps, leg raises, front shoulder press  Recovery: 2 - 5 minutes between each exercise | Regular strength training (body weight/65% - 70% 1rm)  Recovery: 1 - 4 minutes between each exercise | Lower limb (2 × 8) |
| Fischetti, 2019 | RCT | PT - N: 14, CG - N: 14  PT - Age: 26.5, CG - Age: 26.7  Soccer players  Training status: Tier 4 Elite | PT exercises (intensity): jumps over hurdle, drop jumps in stands，horizontal jumps  Recovery: 0.5 - 4 minutes between each exercise | Fartlek and stretching, speed endurance or reaction and general strength training, core stability training, reaction speed and stretching | Lower limb (3 × 12) |
| Attene, 2015 | RCT | PT - N: 18, CG - N: 18  PT - Age: 14.83, CG - Age: 15.2  Basketball players  Training status:Tier 3 Highly Trained / National Level | PT exercises (intensity): front obstacle jumps with knees bending, front obstacle jumps without knees bending, counter movement and jump onto 50 cm box, drop jump from 40 cm box, lunge jump  Recovery: 1 minutes between each exercise | The basketball technique training consisted of 5 min of stationary and non-stationary ball handling with one or two balls, 5 min of jump shooting with single dribble (players performed 1 dribble-quick stop-jump shot) alternating right-side and left-side shots. | Lower limb (2 × 6) |
| Silvia Sedano Campo, 2009 | RCT | PT - N: 10, CG - N: 10  PT - Age: 22.8, CG - Age: 23.0  Soccer players  Spanish National Women’s First Division  Training status: Tier 4 Elite/International Level  Training experience: average 5 years | PT exercises (intensity): jumps over hurdles, drop jumps in stands, horizontal jumps  Recovery: 0.5 - 4 minutes between each exercise | Core stability training, reaction speed and stretching, speed endurance or general strength training, fartlek and stretching | Lower limb (3 × 12) |
| Yosser, 2019 | CT | PT - N: 13, CG - N: 12  PT - Age: 20.9, CG - Age: 21.0  National level female basketball players  Training status: Tier 3 Highly Trained / National Level  Training experience: average 10.8 ± 3.2 years | PT exercises (intensity):  W1 - W4: bounding jumps (0.4 m); hurdle jumps (0.4 m);  W5 - W8: bounding jumps (0.5 m); hurdle jumps (0.5 m)  Recovery: 2 minute between each exercise | Regular basketball training | Lower limb (2 × 8) |
| Yosser, 2021 | NRCT | PT - N: 15, CG - N: 12  PT - Age: 20.9, CG - Age: 21.0  Elite female basketball players  Training status:Tier 3 Highly Trained / National Level  Training experience:Average 10.8 ± 3.2 years | PT exercises (intensity):  W1 - W4:bounding jumps (0.4 m), hurdle jumps (0.4 m), drop jumps  W5 - W8: bounding jumps (0.5 m), hurdle jumps (0.5 m) drop jumps  Recovery: 2 minute between each exercise | Standard basketball training | Lower limb (2 × 8) |
| Helmi, 2021 | NRCT | PT - N: 12, CG - N: 9  PT - Age: 15.9, CG - Age: 15.9  Female adolescent handball players  Training status:Tier 3 Highly Trained / National level  Training experience: No mentioned | PT exercises (intensity): bilateral ankle hops (hurdle height: 20 cm, 10 reps); counter movement jump  Recovery: 1.5 minutes between each exercise | Regular handball training | Lower limb (2 × 8) |
| Ramirez-Campillo, 2016 | RCT | PT - N: 19, CG - N: 19  PT - Age: 22.4, CG - Age: 20.5  Soccer players  Training status:Tier 3 Highly Trained / National Level  Training experience: 12.3 years (PT), 10.6 years (CG) | PT exercises (intensity): 12 jump exercises: cyclic/acyclic, horizontal/vertical, single/double leg involving stretch-shortening cycle muscle activity (Week 1: 2 sets of 5 repetitions per exercise 80 jumps per leg/session, Week 6: Increased to 160 jumps per leg/session, added 1 repetition per set weekly)  Recovery:1.5 minutes between each exercise | Regular soccer training only (no plyometric exercises) | Lower limb (2 × 6) |
| Talukda, 2024 | NRCT | PT - N: 16, CG - N: 15  PT - Age: 13.36, CG - Age: 13.95  Hockey, football, water polo and netball players  Train status: Tier 2 Trained/Developmental  Training experience: 1 year | PT exercises (intensity): box jumps (height increased), single-leg bounds (distance increased), hurdle jumps (unilateral/bilateral, low ground contact time); medicine ball slams (weight increased); ground contacts increased from 150/week to 250/week  Recovery:1 - 2 minute between each exercise | Regular physical education classes with non-specific endurance activities | Lower and upper limb (2 × 7) |
| Trajković, 2016 | RCT | PT - N: 22, CG - N: 20  PT - Age: 16.36, CG - Age: 16.16  Volleyball players  Training status: Tier 4 Elite/International Level  Training experience: 3-5 year | PT exercises (intensity): box jumps, hurdle jumps, depth jumps, lateral jumps over boxes, lunge jumps, vertical jumps  Week 1 - 2: moderate intensity, 2 - 3 sets of 6 - 10 repetitions, box height 40 - 50 cm  Week 3 - 4: high intensity, 3 - 4 sets of 10 - 12 repetitions, box height 50 - 60 cm  Week 5 - 6: moderate-high intensity, 3 sets of 9 - 12 repetitions, mixed box heights 20 - 60 cm  Recovery: 1 minute between each exercise | Regular moderate intensity volleyball training only: standard volleyball drills for technique and match simulation. matches and skill repetitions without specific power-focused interventions | Lower limb (12 × 6) |
| Guimarães, 2023 | RCT | PT - N: 9, CG - N: 8  PT - Age: 19.0, CG - Age: 19.0  Volleyball players  Training status:Tier 3 Highly Trained / National Level  Training experience: 6.2 year | PT exercises (intensity): each pt session consisted of 8 sets of jumps. there were four sets of 10 consecutive maximum jumps with an additional load (a bar at the cervical region, 20% of 1rm); 4 sets of 15 - second maximum; counter movement jump  Recovery: 1.5 - 2 minute between each exercise | Underwent friendly matches, technical, tactical and resistance training | Lower limb (2 × 4) |
| Krističević, 2016 | NRCT | PT - N: 27, CG - N: 27  PT - Age: 15.4, CG - Age: 15.5  Volleyball players  Train status : Tier 2 Trained/Developmental  Training experience: 3 year | PT exercises (intensity):hurdle jumps (box height 30 cm); depth jumps (box height 30 cm); lateral jumps over box (box height 20 cm); lunge jumps; vertical jumps  Recovery:2 minutes between each exercise | Conduct regular volleyball training and moderate intensity continuous training | Lower limb (2 × 5) |
| Maciejczyk, 2021 | RCT | PT - N: 7, CG - N: 8  PT - Age: 21.0, CG - Age: 18.0  Soccer players  Training status: Tier 4 Elite/International Level  Training experience: 9.75 years (PT), 8.9 years (CG) | PT exercises (intensity): double - leg/single - leg jumping over vertical obstacles (volume - no. of contacts 107); side jumping to target areas, round-trip jumping (volume - no. of contacts 133); diagonal obstacle jumping and turning (volume - no.of contacts 159); forward jumping, side jumping, turning (volume - no.of contacts 125)  Recovery: 2 minutes between each exercise | Follow the same regular training plan, but do not perform plyometric training | Lower limb (2 × 4) |
| Meszler, 2019 | RCT | PT - N: 7, CG - N: 8  PT - Age: 21.0, CG - Age: 18.0  Basketball players  Train status : Tier 2 Trained/Developmental  Training experience: 5 years | PT exercises (intensity): W1-W3: double - leg hurdle jump (50 cm); single - leg lateral cone jump (25 cm), single - leg forward hop, double - leg depth jump (25 cm), double - leg lateral cone jump (35 cm), single - leg hurdle jump (25 cm)  W4-W6: double - leg hurdle jump (50 cm), single - leg lateral cone jump (25 cm), single - leg forward hop, double - leg depth jump (25 cm), double - leg lateral cone jump (35 cm), single - leg hurdle jump (25 cm)  W7: double - leg hurdle jump (50 cm), single - leg lateral cone jump (25 cm), single - leg forward hop, double - leg depth jump (25 cm), double - leg lateral cone jump (35 cm), single - leg hurdle jump (25 cm)  Recovery: 2 minute between each exercise | Follow the same regular training plan | Lower limb (2 × 7) |
| Nonnato, 2022 | RCT | PT - N: 8, CG - N: 8  PT - Age: 23.0, CG - Age: 23.0  Professional female soccer players  Training status: Tier 4 Elite/International Level  Training experience: 5 years | PT exercises (intensity): hurdle jumps (10 cm), hurdle jumps (30 cm), lateral and horizontal jumps, box jumps (30 cm), 30 cm drop jump followed by a 30 cm hurdle jump.  Recovery: 2 minutes between exercises. | The CON performed a recovery session that mainly consisted of balance exercises and dynamic stretching. They did not carry out any plyometric exercises during the experimental period. | Lower limb (1 × 12) |
| Ozbar, 2015 | NRCT | PT - N: 10, CG - N: 10  PT - Age: 19.4, CG - Age: 18.0  Soccer players  Training status: Tier 3 Highly Trained / National Level  Training experience: 5 years | PT exercises (intensity): W1-2: standing long jump, front cone hops, horizontal jumps over hurdle, single leg lateral jump, forward-backward run.  w3 - 4: double leg horizontal jump, lateral jump over hurdle, side-to-side sprint, jump over low hurdle, vertical, lateral & horizontal jump, skipping with change of direction sprint.  w5 - 6: split squat jump, front cone hops and 4 × 6 m change of direction sprint, lateral jump over hurdle, side-to-side slide and hops, lateral & horizontal jump, step, jump, down, up and sprint  W7 - 8: cone hops with 180° turn, vertical, lateral and horizontal jump, lateral jump over hurdle, diagonal jump over hurdle, single leg lateral jump, step, jump, down, up and sprint, slaloming  W9 - 10: diagonal jump, standing long jump and diagonal sprint, single leg vertical jump, cone hops with 180° turn, skipping over cone, double leg diagonal jump over hurdle, single leg lateral and horizontal jump, cone hops with change of direction sprint  Recovery: 1 minute between each exercise | The control group did not participate in the PT. Players were not allowed to do any other physical activity during the research. | Lower limb (2 × 10) |
| Ozbar, 2014 | RCT | PT - N: 9, CG - N: 9  PT - Age: 18.4, CG - Age: 18.4  Professional female soccer players  Training status:Tier 3 Highly Trained / National Level  Training experience: 4 years | PT exercises (intensity): horizontal jumps over hurdles (20 - 40 cm); standing long jumps; front cone hops, and forward-backward runs; split squat jumps; lateral jumps over hurdles; cone hops with direction changes; single-leg lateral jumps; diagonal jumps over taller hurdles (40 - 60 cm); single-leg vertical/horizontal jumps; skipping with sprinting, and slaloming  Recovery: 1 minute between each exercise | Only regular soccer training, including 4 day weekly practices (focused on dribbling, passing, tackling, small-sided games, etc.) and 1 game per week | Lower limb (1 × 8) |
| Falch, 2022 | RCT | PT - N: 11, CG - N: 10  PT - Age: 17.5, CG - Age: 17.1  The academy of an elite team playing at the second-highest level in the Norwegian league system.  Training status:Tier 3 Highly Trained / National Level | PT exercises (intensity): drop jump, unilateral cmj, skate-jump  Recovery: 2 minute between each exercise | Bilateral squat; unilateral squat; lateral squat | Lower limb (1.5 × 8) |
| Kale, 2016 | NRCT | PT - N: 10, CG - N: 9  PT - Age: 20.4, CG - Age: 19.4  Handball players  Training status: Tier 3 Highly Trained / National Level  Training experience: 4 years | PT exercises (intensity): hurdle jumps, lateral/frontal multi jumps (3 sets × 12 reps)  Recovery: 3 minutes between each exercise | Regular training | Lower limb (2 × 6) |
| Gaamouri, 2023 | RCT | PT - N: 14, CG - N: 14  PT - Age: 15.7, CG - Age: 15.8  Handball players  Training status: Tier 3 Highly Trained / National Level  Training experience: 5 years | PT exercises (intensity): Push-up (10 sets × 6 reps); Hurdle jump (0.3 m height × 2 sets × 6 reps); Lateral hurdle jump (0.3 m height × 2 sets × 6 reps); Stretched leg jump (0.25 m height × 2 sets × 6 reps); Hurdle jump (180° rotation, 0.25 m height × 2 sets × 6 reps); Horizontal jump (1.1 m × 2 sets × 6 reps)  Recovery: 0.5 minute between each exercise | Followed their usual handball training (i.e. mainly technical-tactical exercises, small-sided and simulated games, or injury prevention drills) | Lower limb (2 × 10) |
| Wang, 2021 | RCT | PT - N: 5, CG - N: 5  PT - Age: 16.2, CG - Age: 16.2  High school korfball players  Train status: Tier 2 Trained/Developmental | PT exercises (intensity):  w1 - w4: double-leg ankle jumps (30 s × 2 sets), backward jumps (30 s × 2 sets), single-leg left-right ankle jumps (30 s × 2 sets), alternating pedal jumps (30 s × 2 sets), lateral step-ups (30 s × 2 sets), back pull passing (15 reps × 2 sets)  W4 - W8: single-leg lateral jumps (30 s × 2 sets), plyometric box multiple jumps (5 reps × 2 sets), explosive single-leg jumps (5 reps × 2 sets), depth jump with 180° twist (5 reps × 2 sets) explosive squat with overhead medicine ball throw (5 reps × 2 sets), kneeling side medicine ball throws (10 reps × 2 sets)  W8-W12: split squat cross jumps (15 reps × 2 sets), single-leg depth jumps (10 reps × 2 sets), plyometric box pyramid jumps (5 reps × 2 sets), zig-zag drills (0 ms × 2 sets), depth jump with lateral movement (10 m × 2 sets), sit-up with overhead medicine ball throw (15 reps × 2 sets)  Recovery: 1 minute between each exercise | W1-W4: weighted half squat (8 reps × 6 set), back squat (8 reps × 6 sets), seated weighted calf raise (8 reps × 6 sets), lunge jump (10 reps × 6 sets), 60 m sprint (3 sets)  W4-W8: weighted barbell half squat jump (10 reps × 6 sets), barbell squat jump (10 reps × 6 sets), weighted half squat (10 reps × 6 sets), weighted back squat (10 reps × 6 sets), seated weighted calf raise (10 reps × 6 sets), 60 m sprint;  W8-W12: back squat with barbell on shoulders (8 reps × 4 sets), barbell squat to acceleration run (30 m × 4 sets), barbell single-leg stance (8 reps × 4 sets), weighted half squat (10 reps × 8 sets), Weighted back squat (10 reps × 8 sets) | Lower and upper limb (3 × 12) |

**Appendix D.** Statistical power diagrams

| 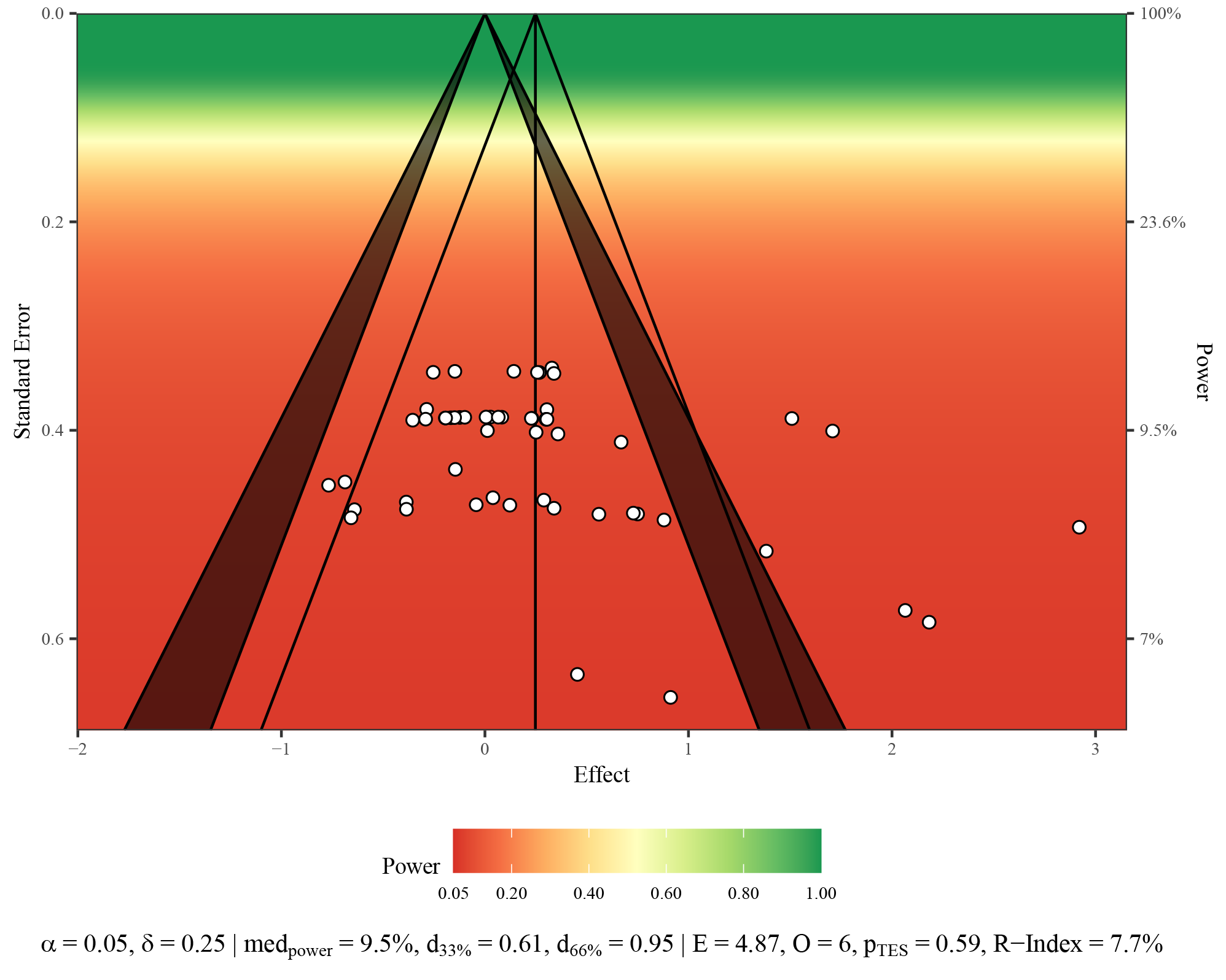 | 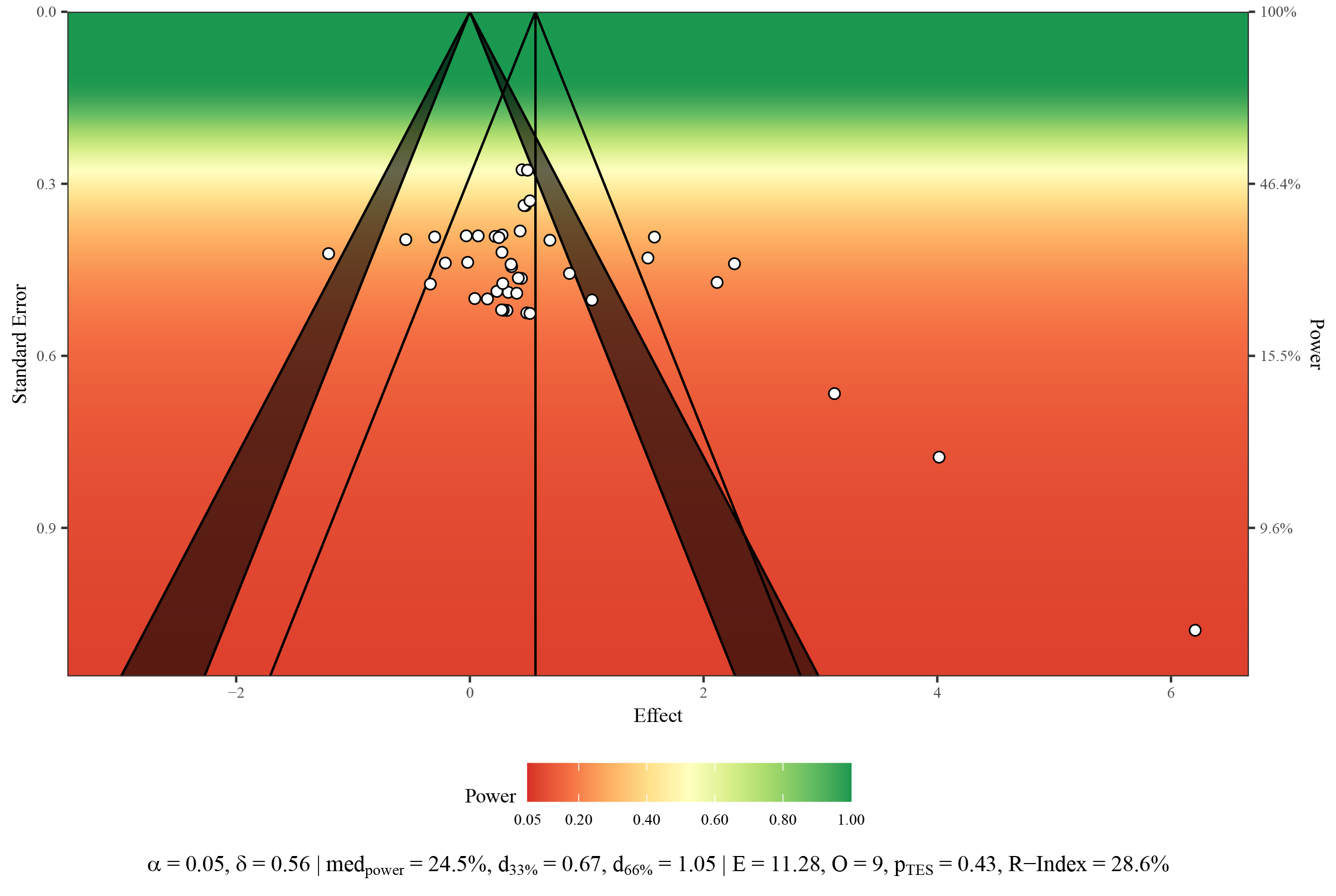 |
| --- | --- |
| (a) | (b) |
| 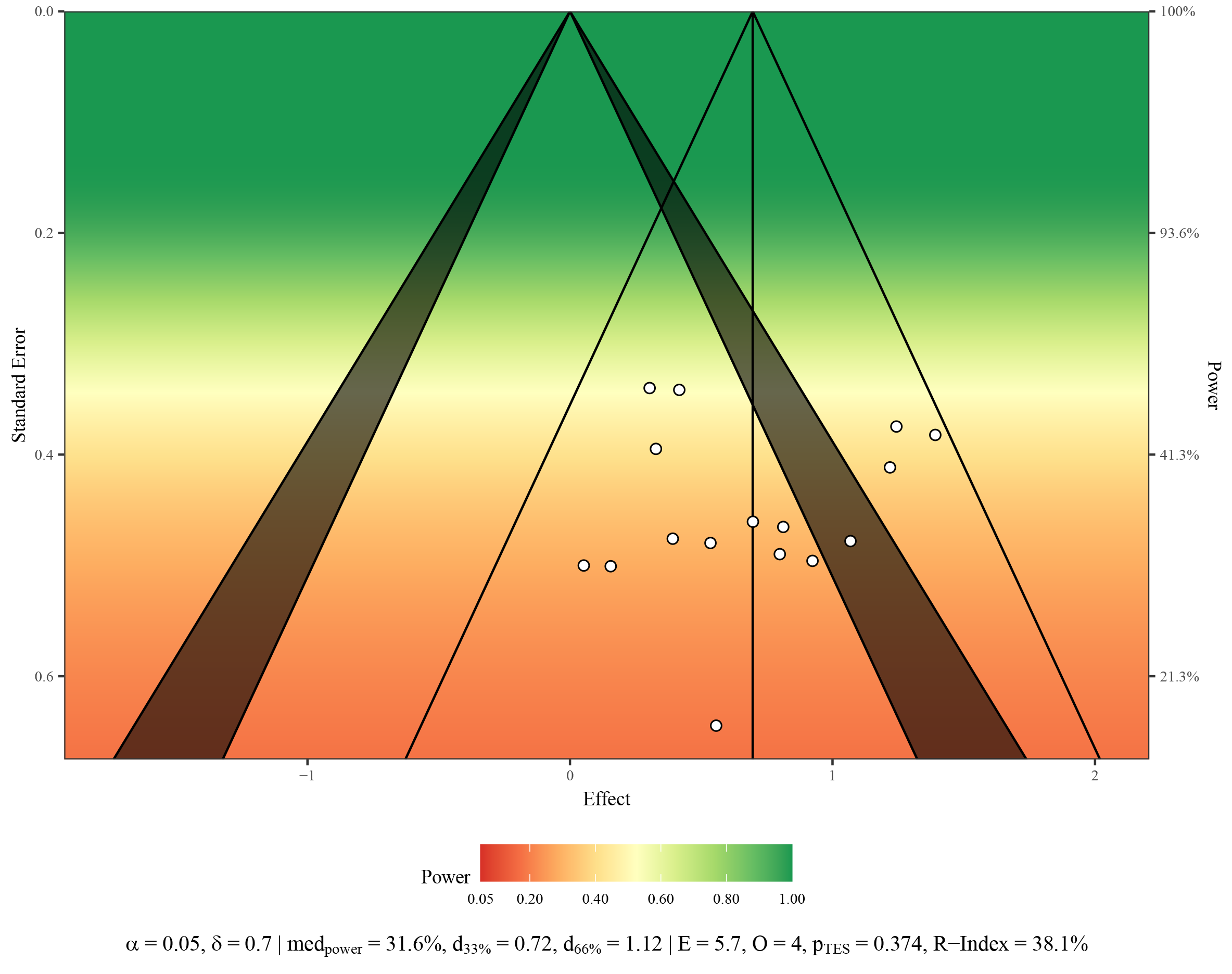 | 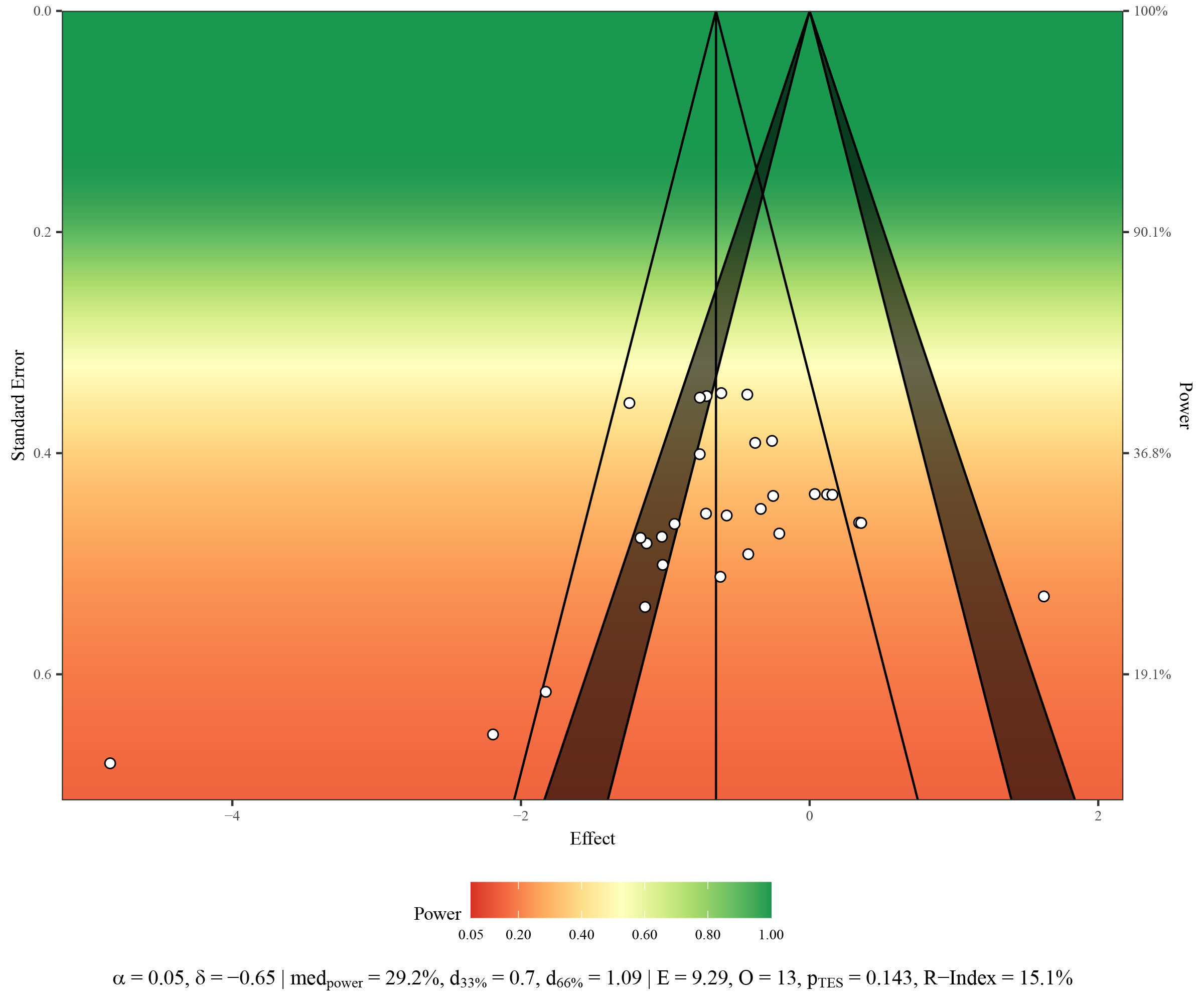 |
| (c) | (d) |
| 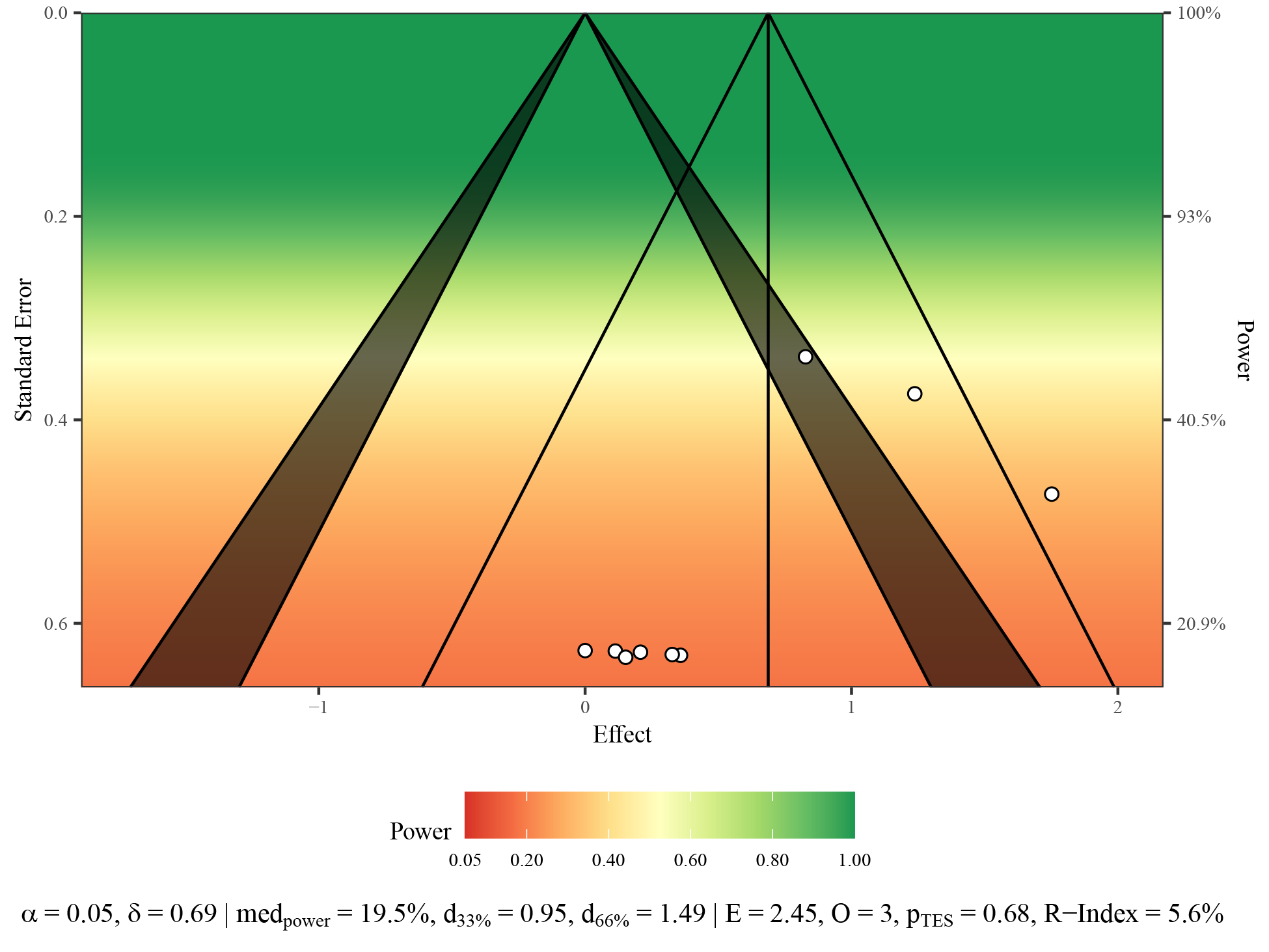 | 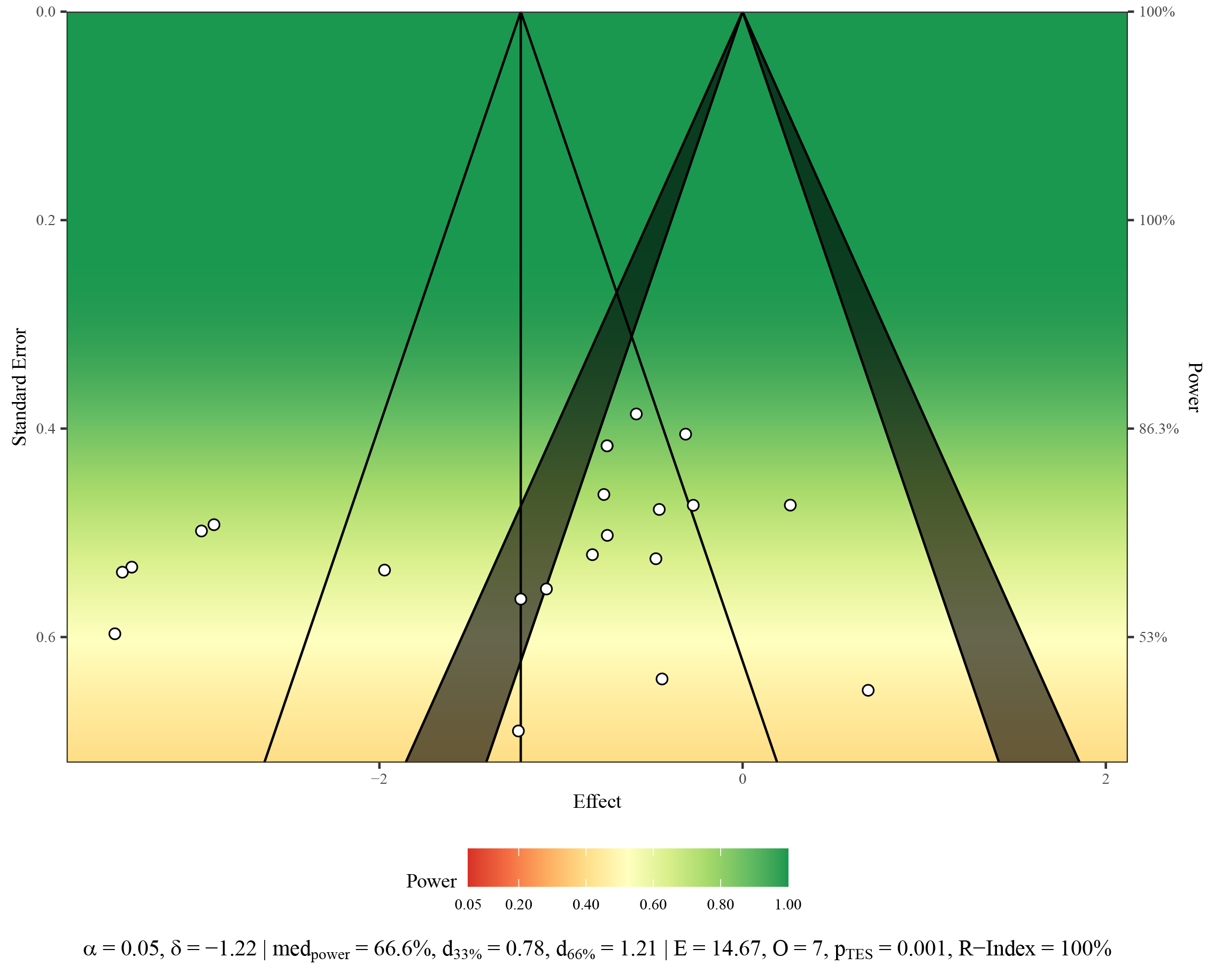 |
| (e) | (f) |

**Figure D1a-1f.** Sunset statistical power charts: (**a**) shows the plot for strength; (**b**) shows the plot for vertical jump performance; (**c**) shows the plot for horizontal jump performance; (**d**) shows the plot for sprint performance; (**e**) shows the plot for throwing performance; (**f**) shows the plot for agility performance.

**Appendix E.** Publication bias funnel plots

| 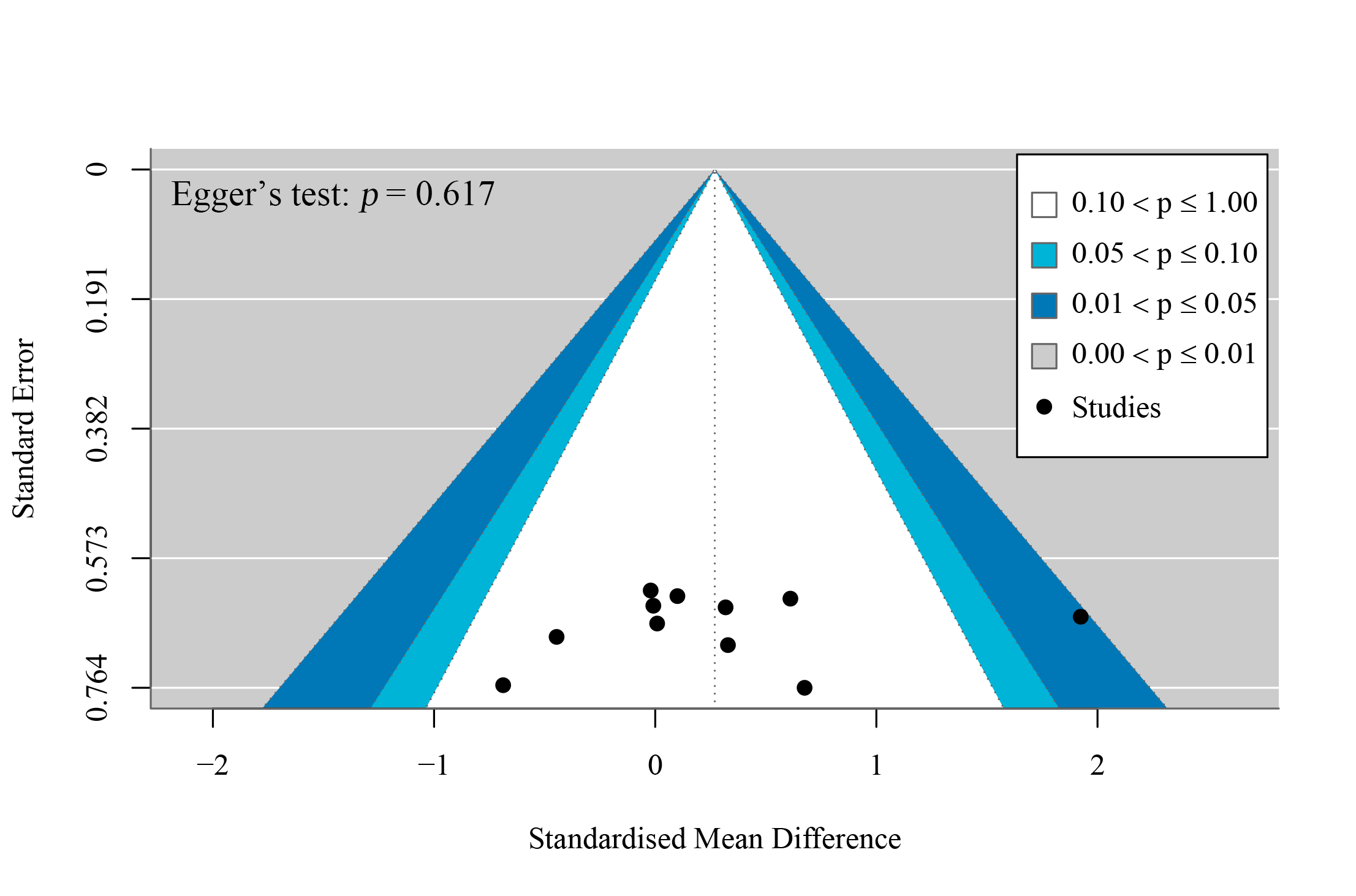 | 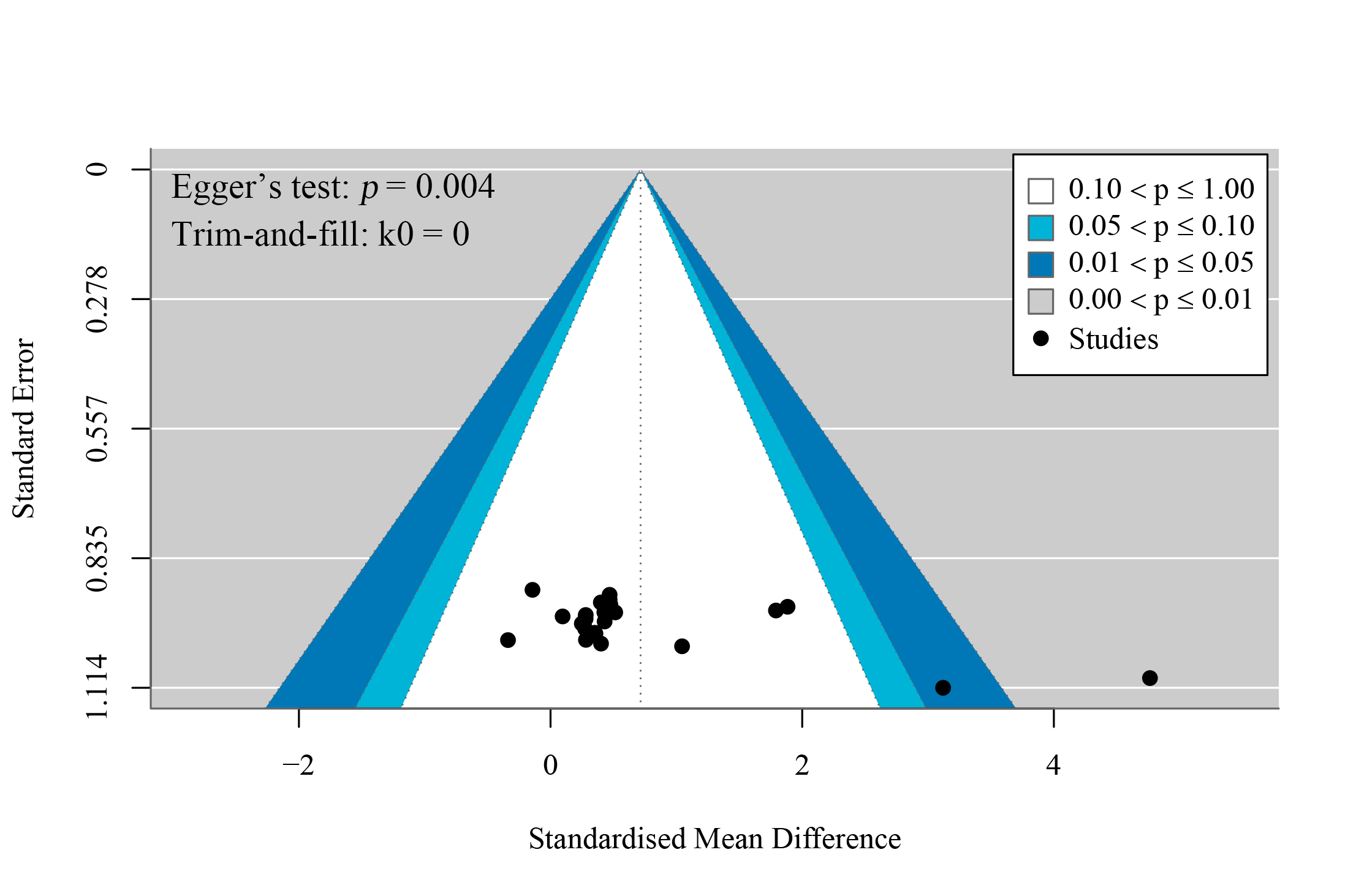 |
| --- | --- |
| (a) | (b) |
| 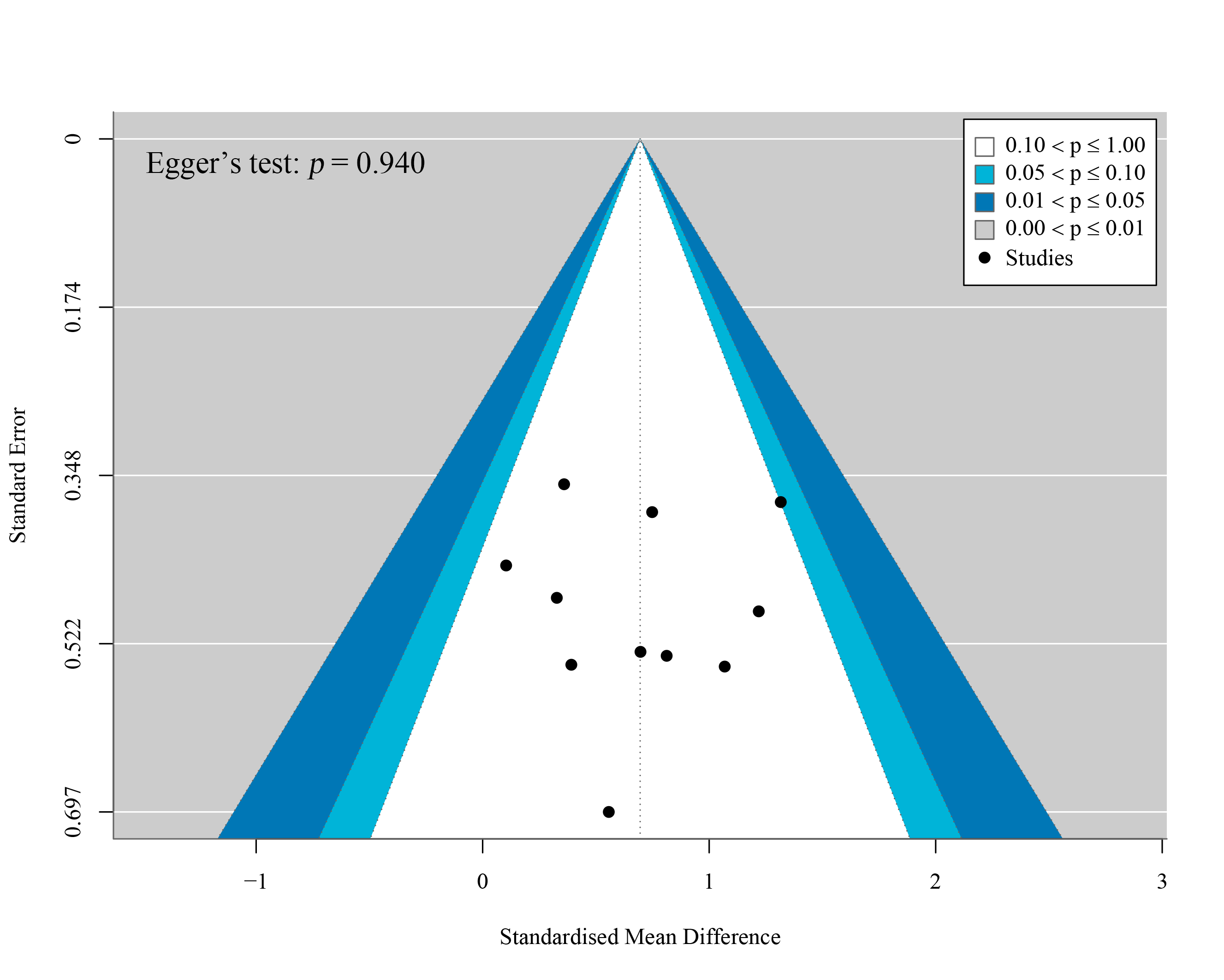 | 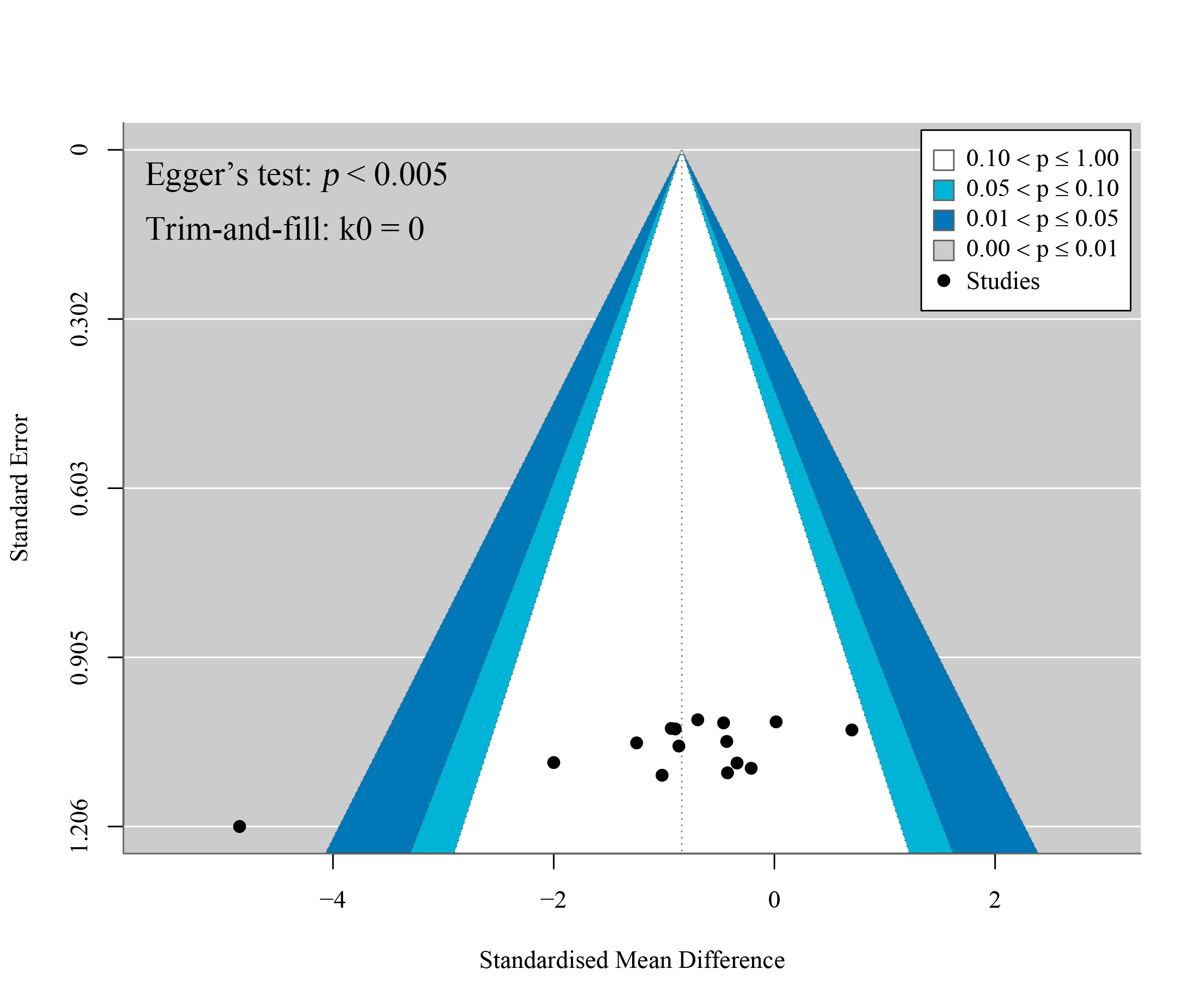 |
| (c) | (d) |
| 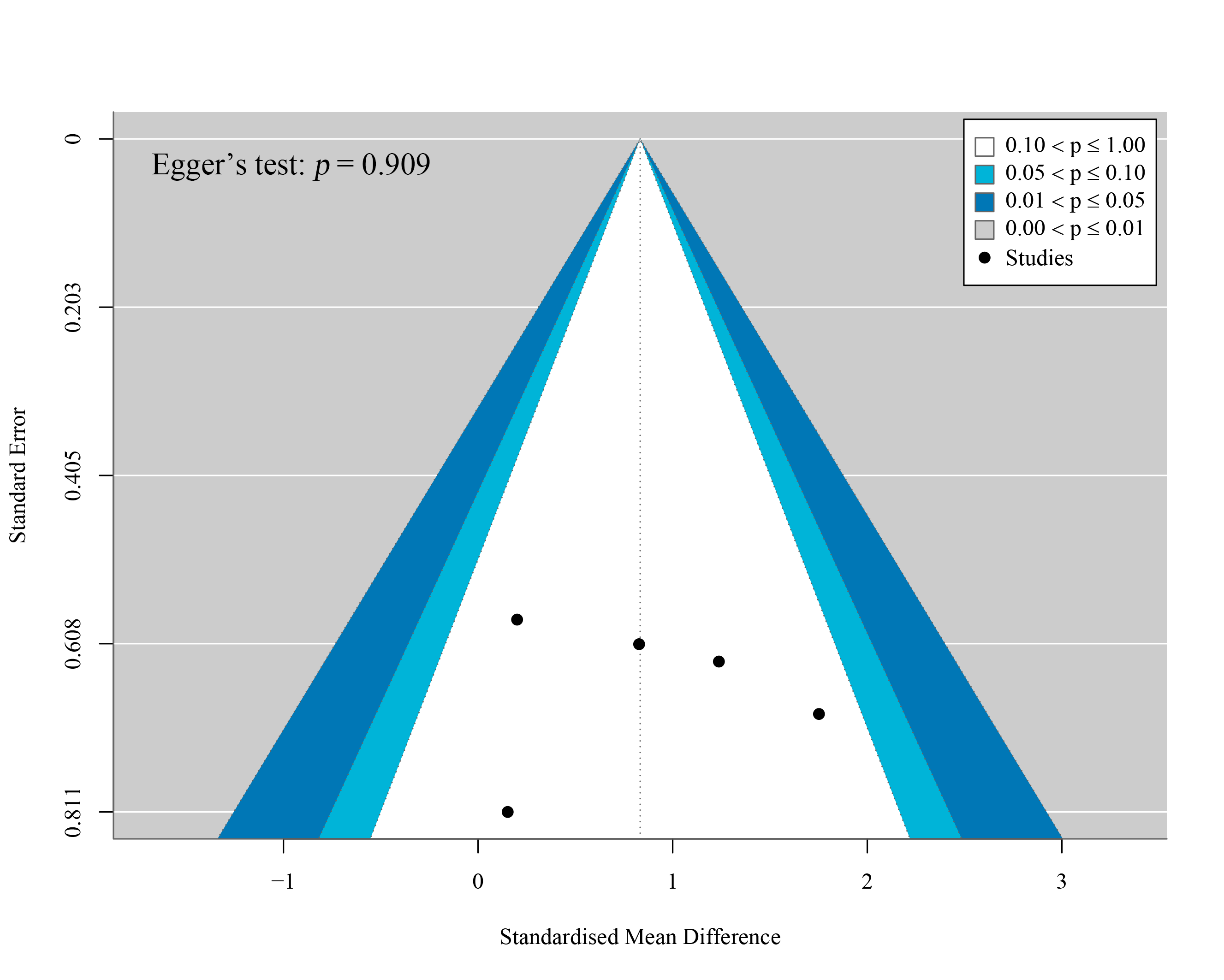 | 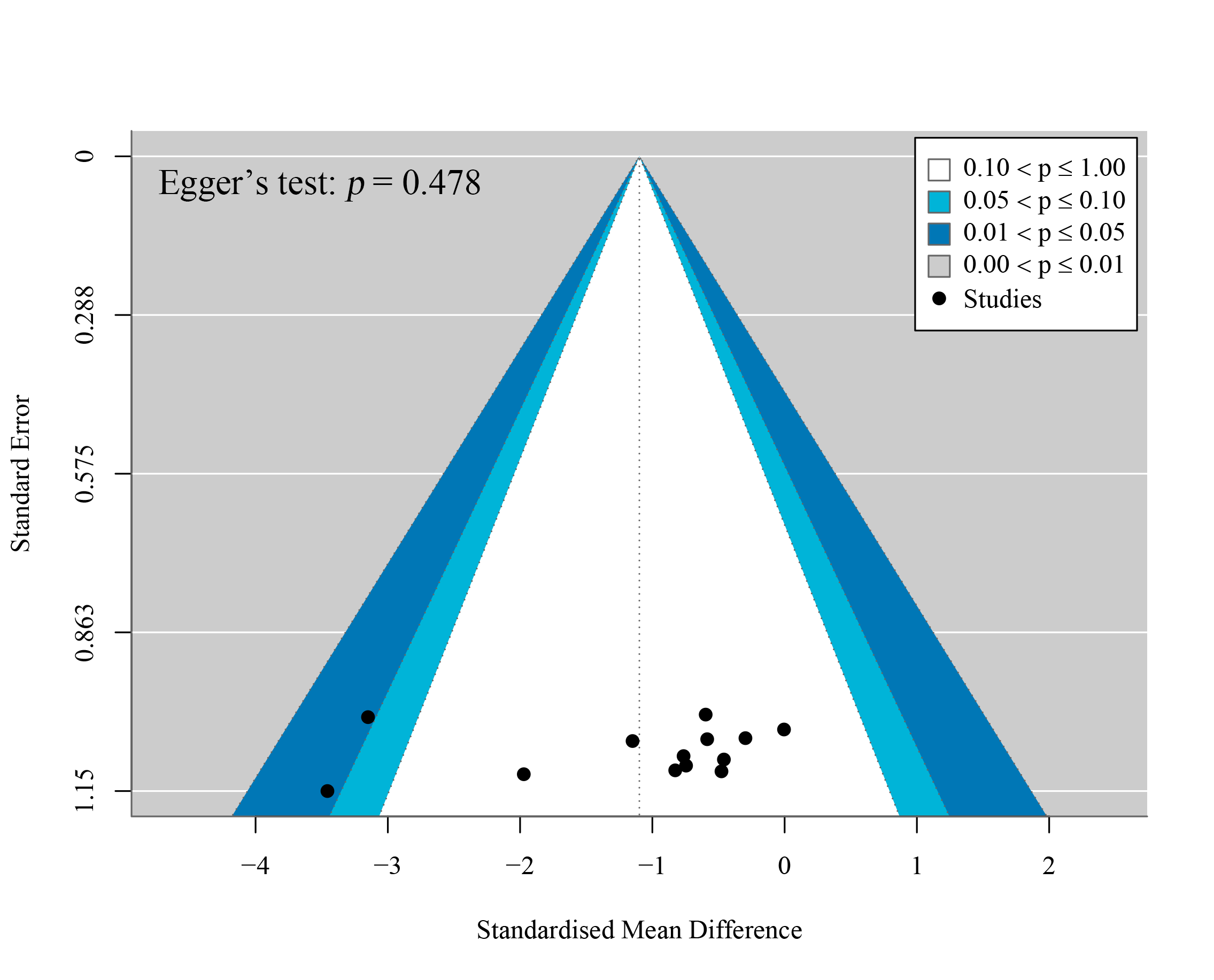 |
| (e) | (f) |

**Figure E1a-1f.** Funnel plots: (**a**) is the funnel plot for strength; (**b**) is the funnel plot for vertical jump performance; (**c**) is the funnel plot for horizontal jump performance; (**d**) is the funnel plot for sprint performance; (**e**) is the funnel plot for throwing performance; (**f**) is the funnel plot for agility performance.

**Appendix F.** The forest plot in primary results

| **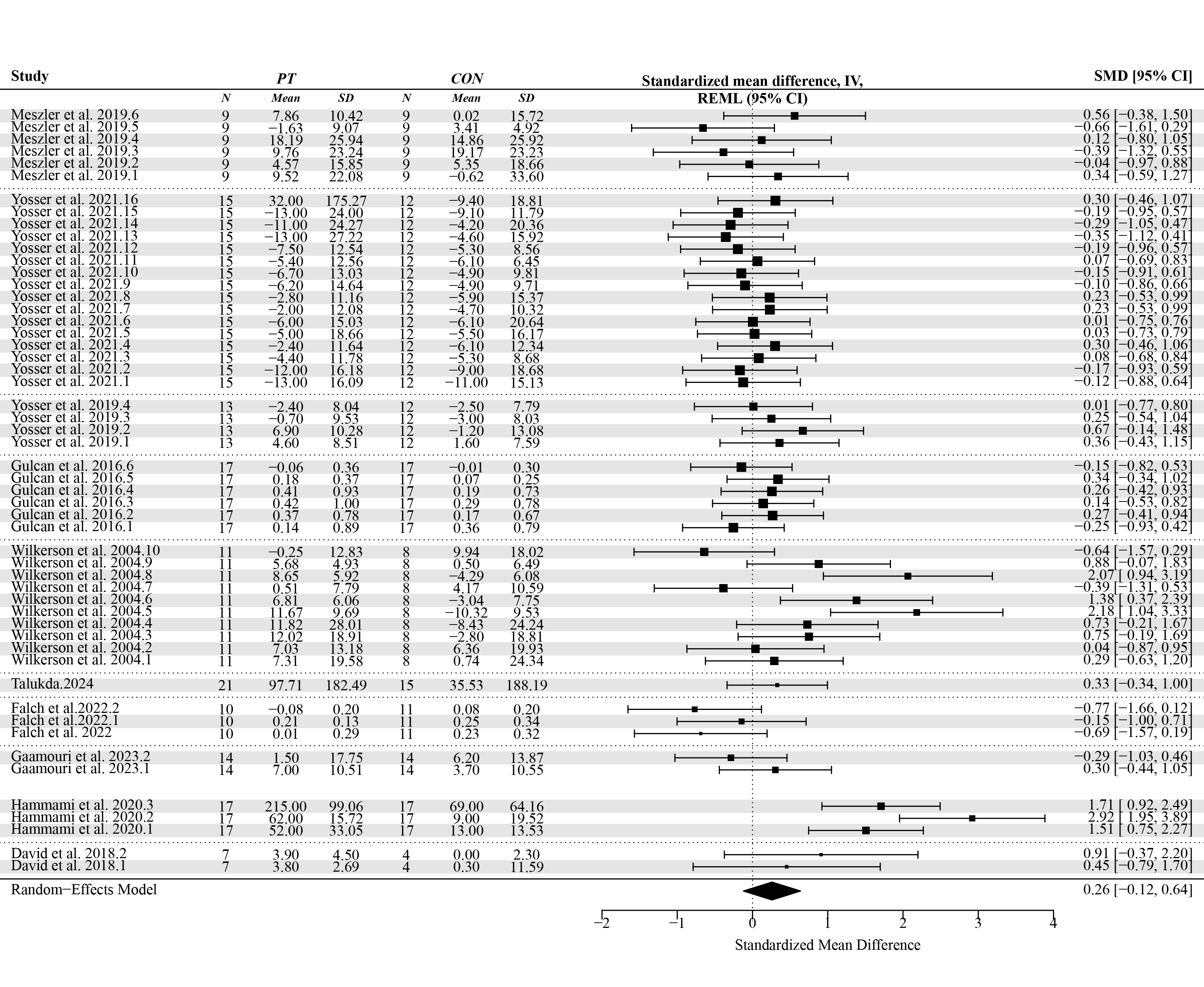** |
| --- |
| **Figure F1.** Strength Forest Plot |
| **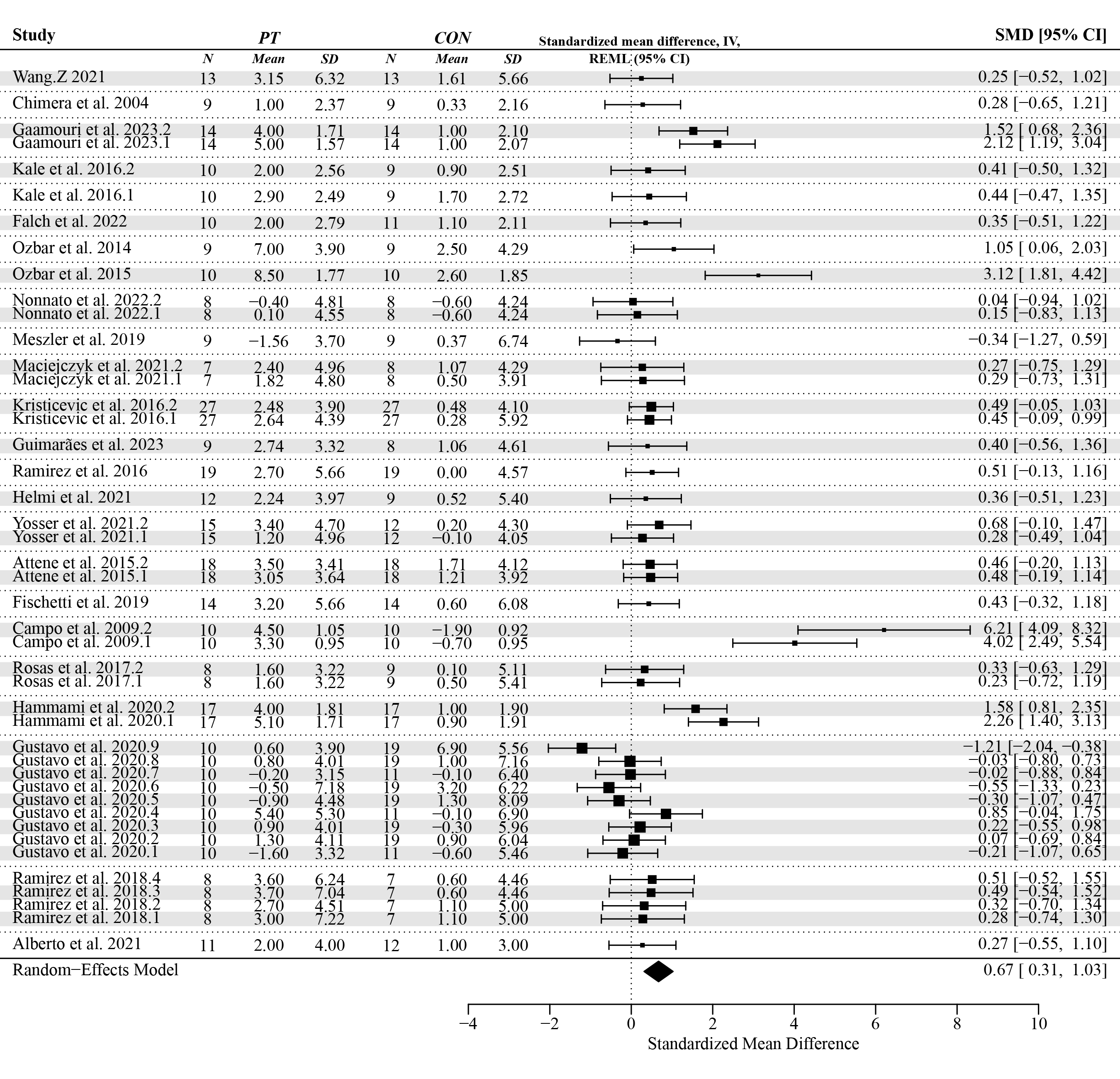** |
| **Figure F2.** Vertical jump performance Forest Plot |
| **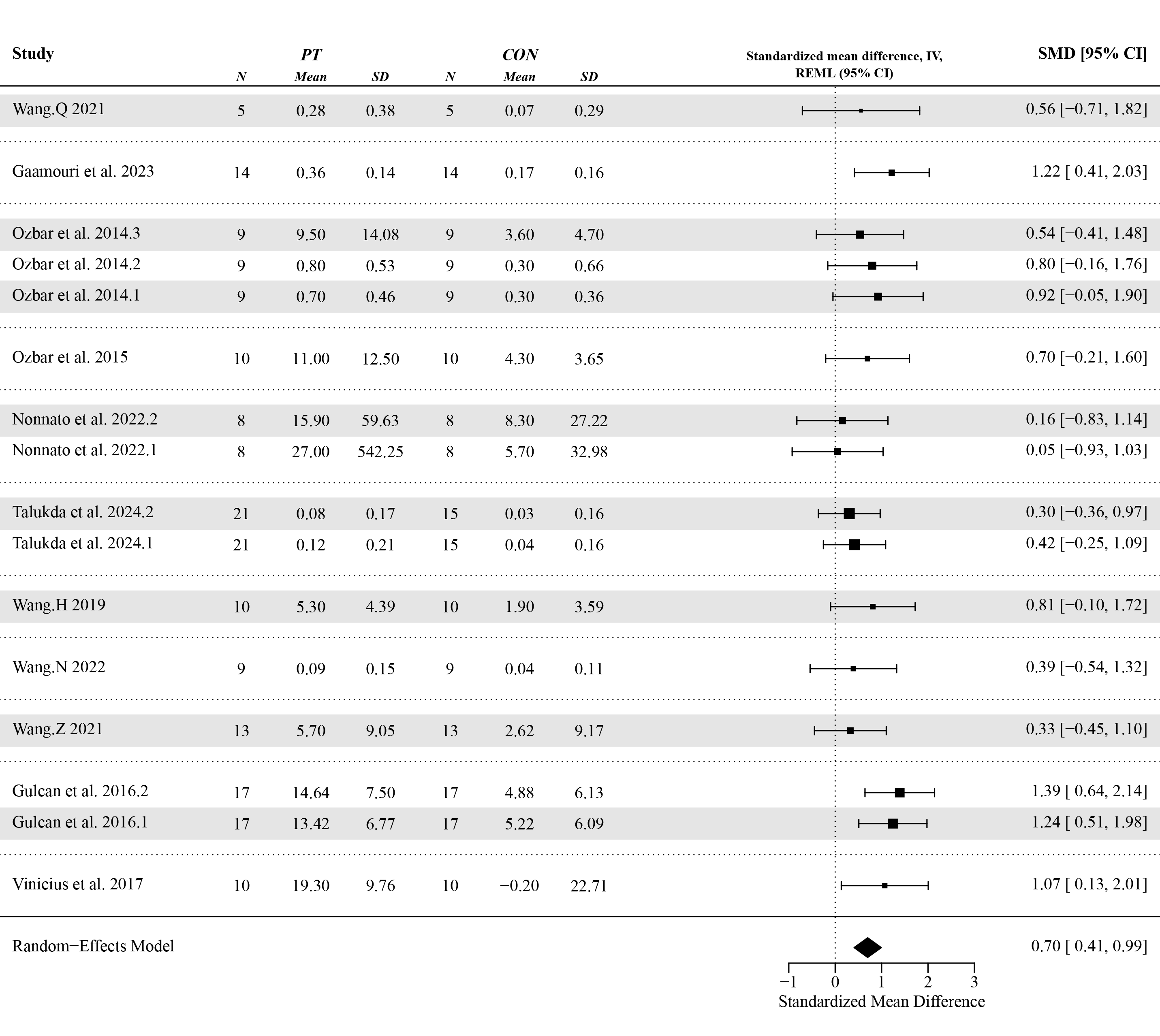** |
| **Figure F3.** Horizontal jump performance Forest Plot |
| **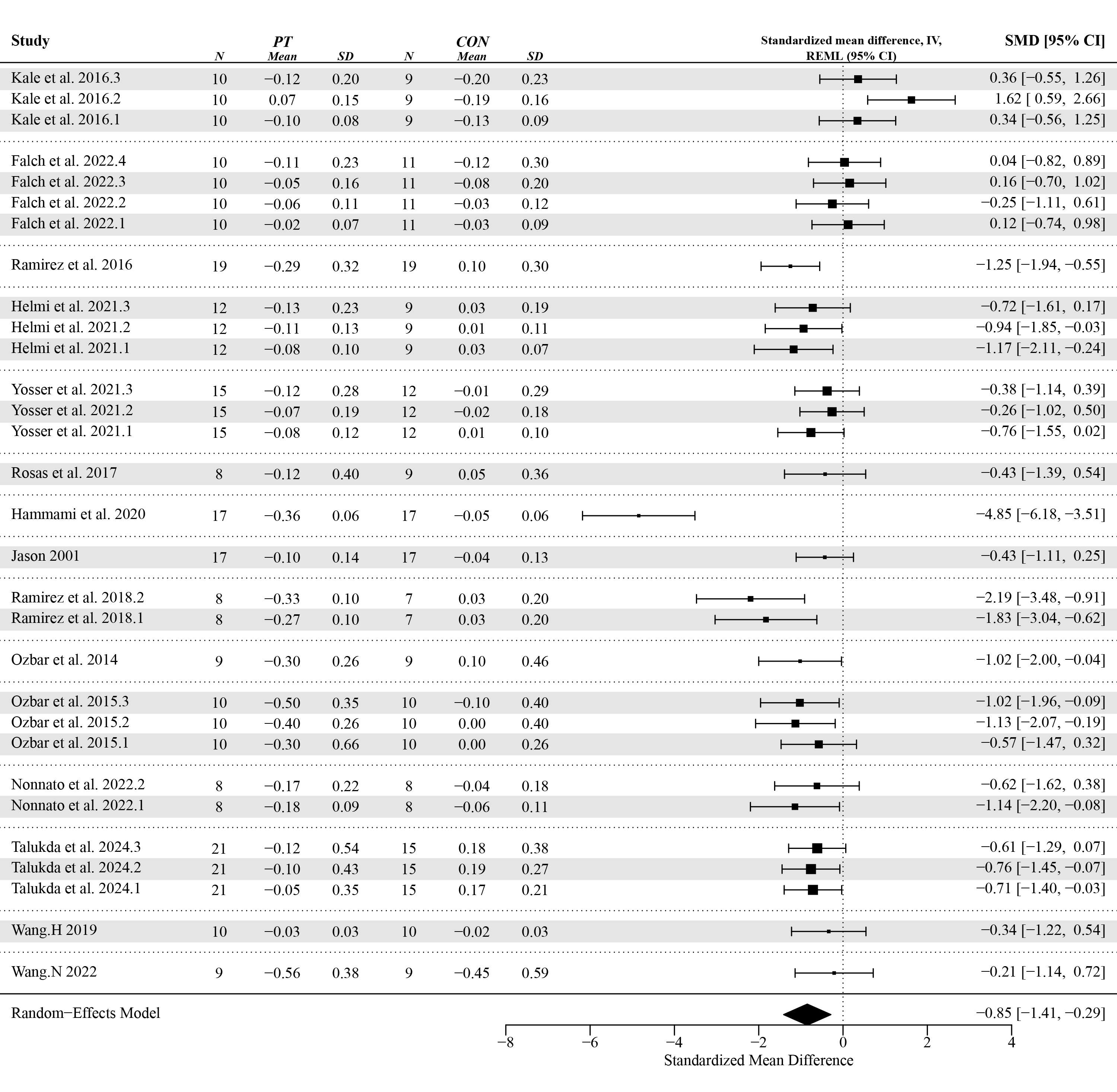** |
| **Figure F4.** Sprint performance Forest Plot |
| **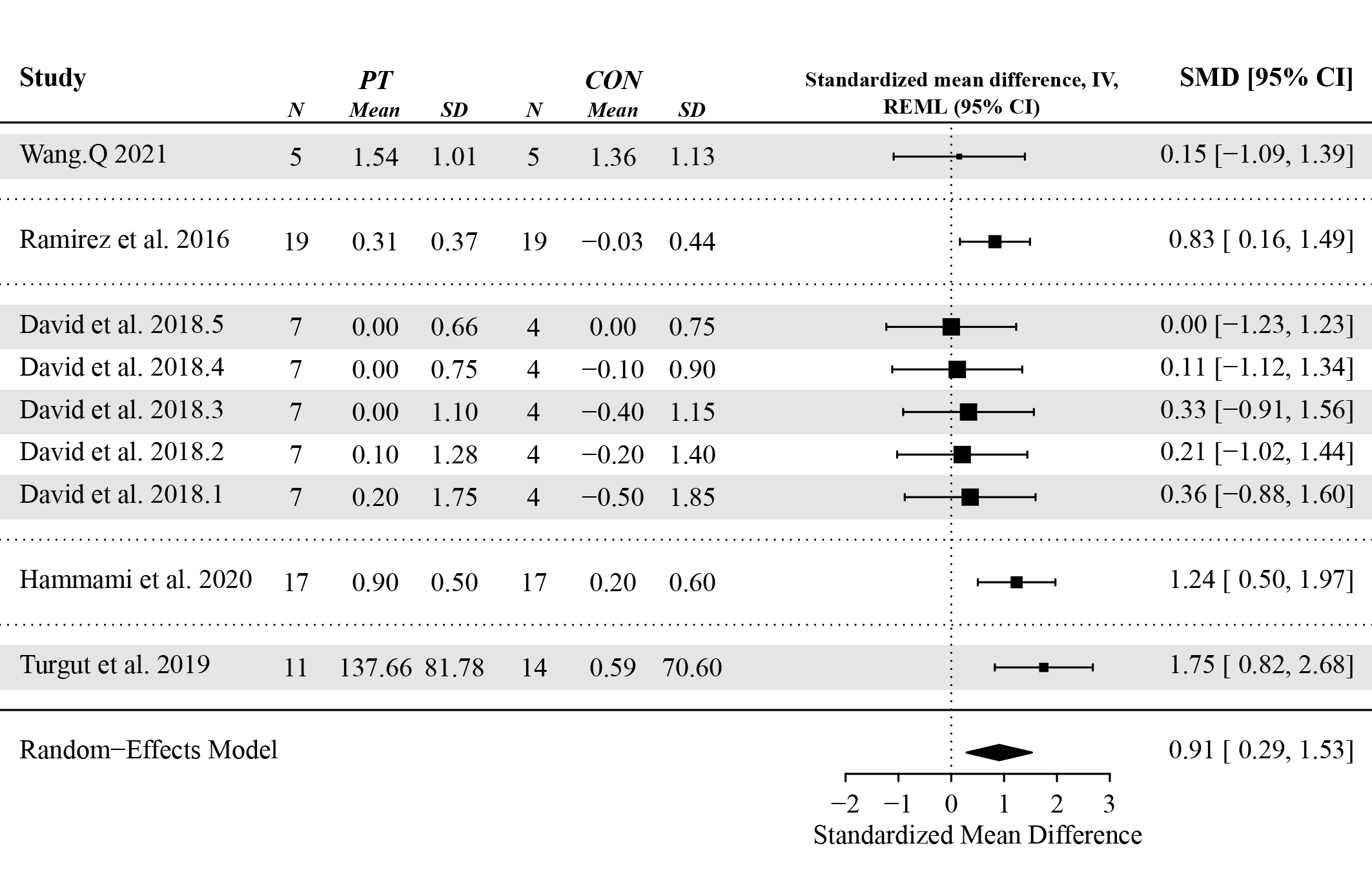** |
| **Figure F5.** Throwing performance Forest Plot |
| **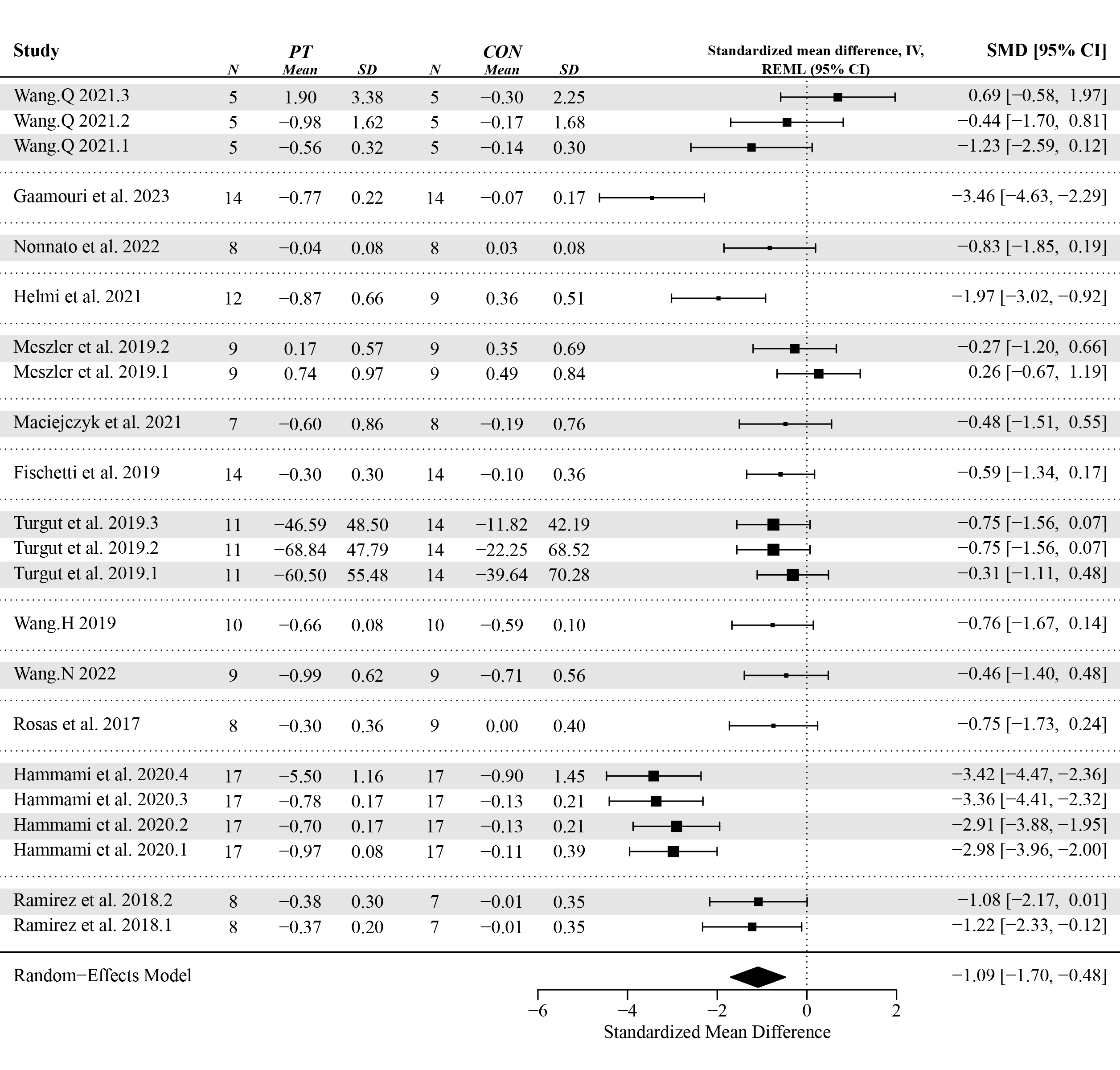** |
| **Figure F6.** Agility performance Forest Plot |

**Appendix G. Sensitivity Analysis Plot**

| **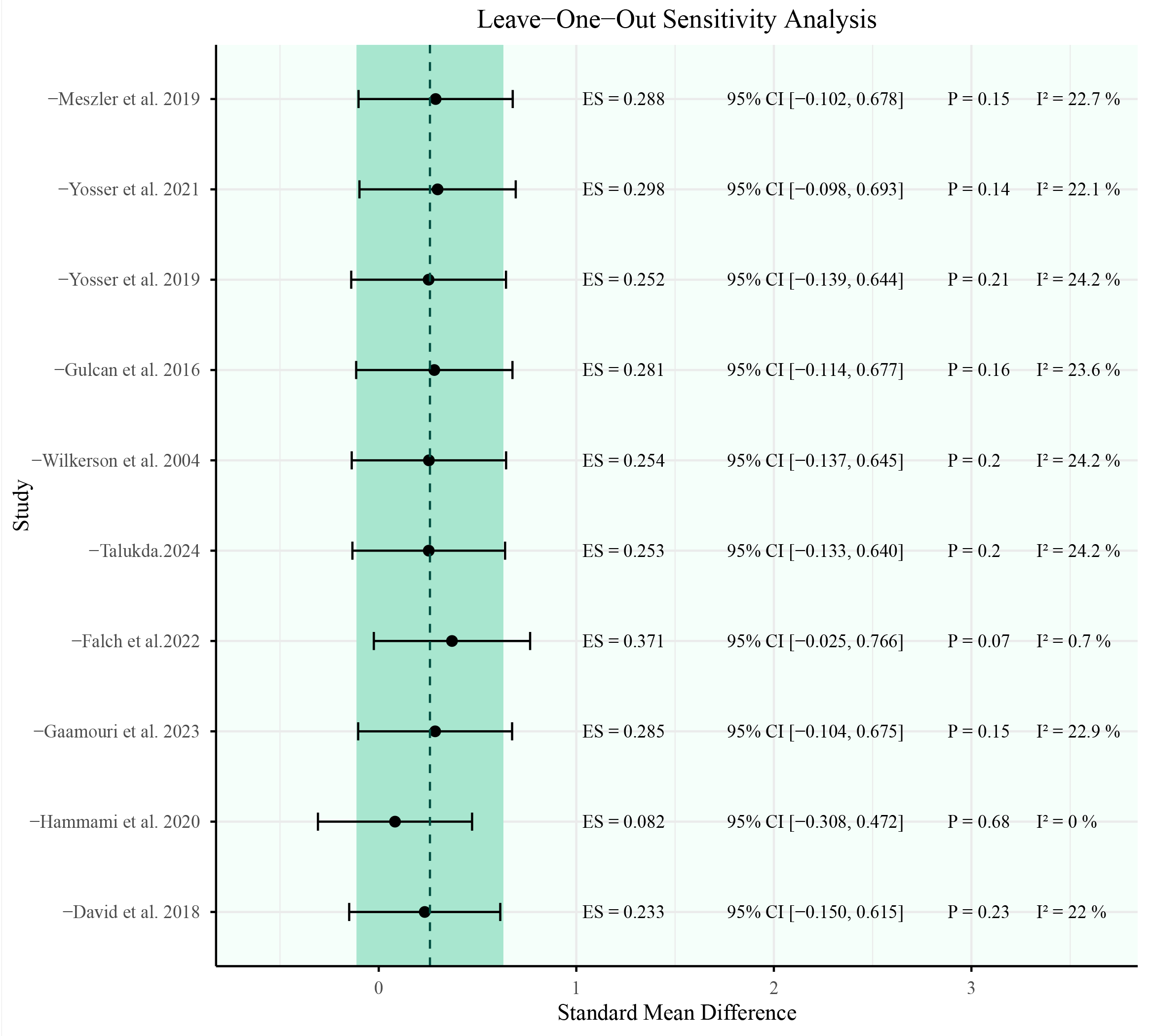** |
| --- |
| **Figure G1.** Strength Sensitivity Analysis Plot |
| **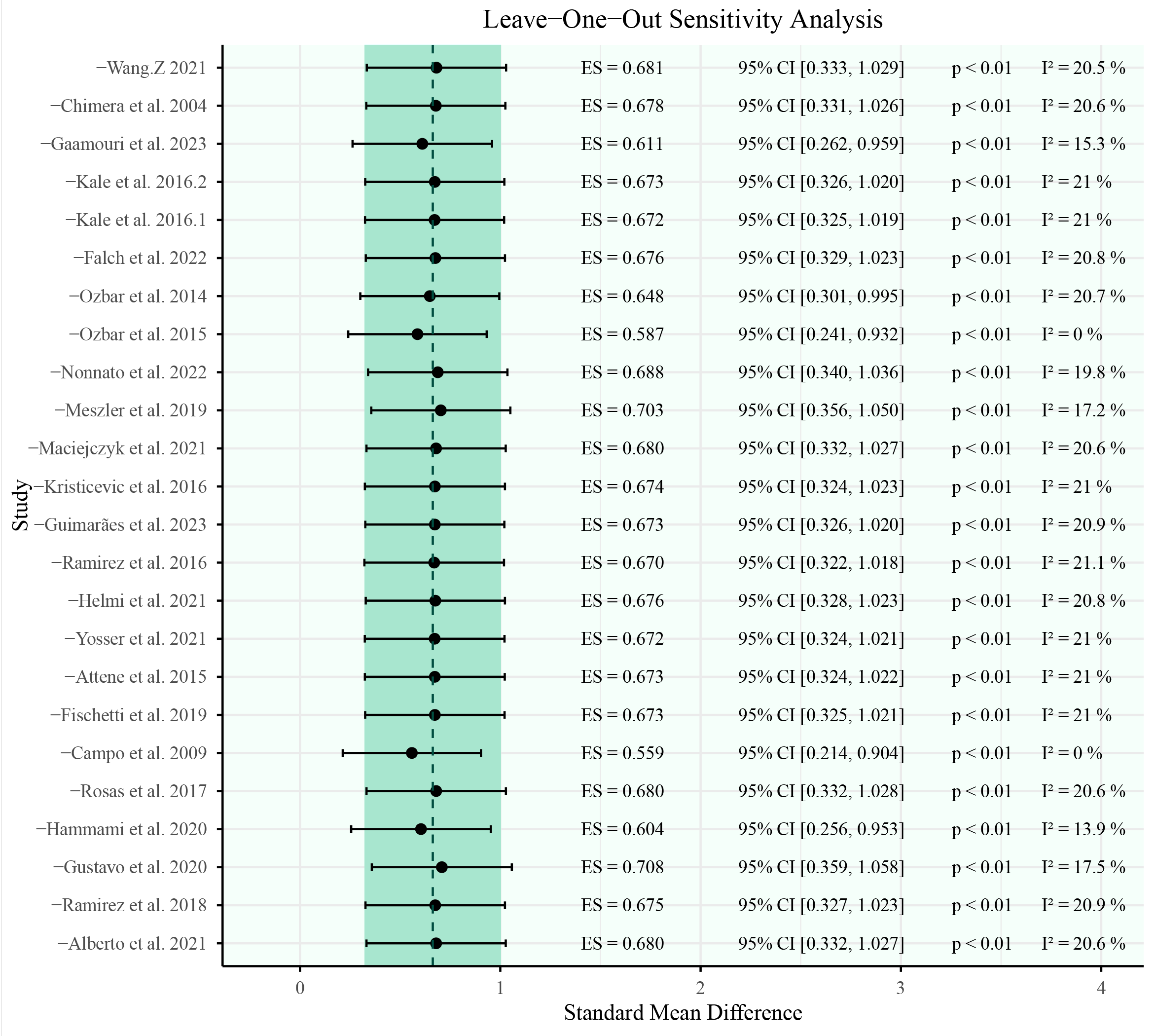** |
| **Figure G2.** Vertical jump performance Sensitivity Analysis Plot |
| **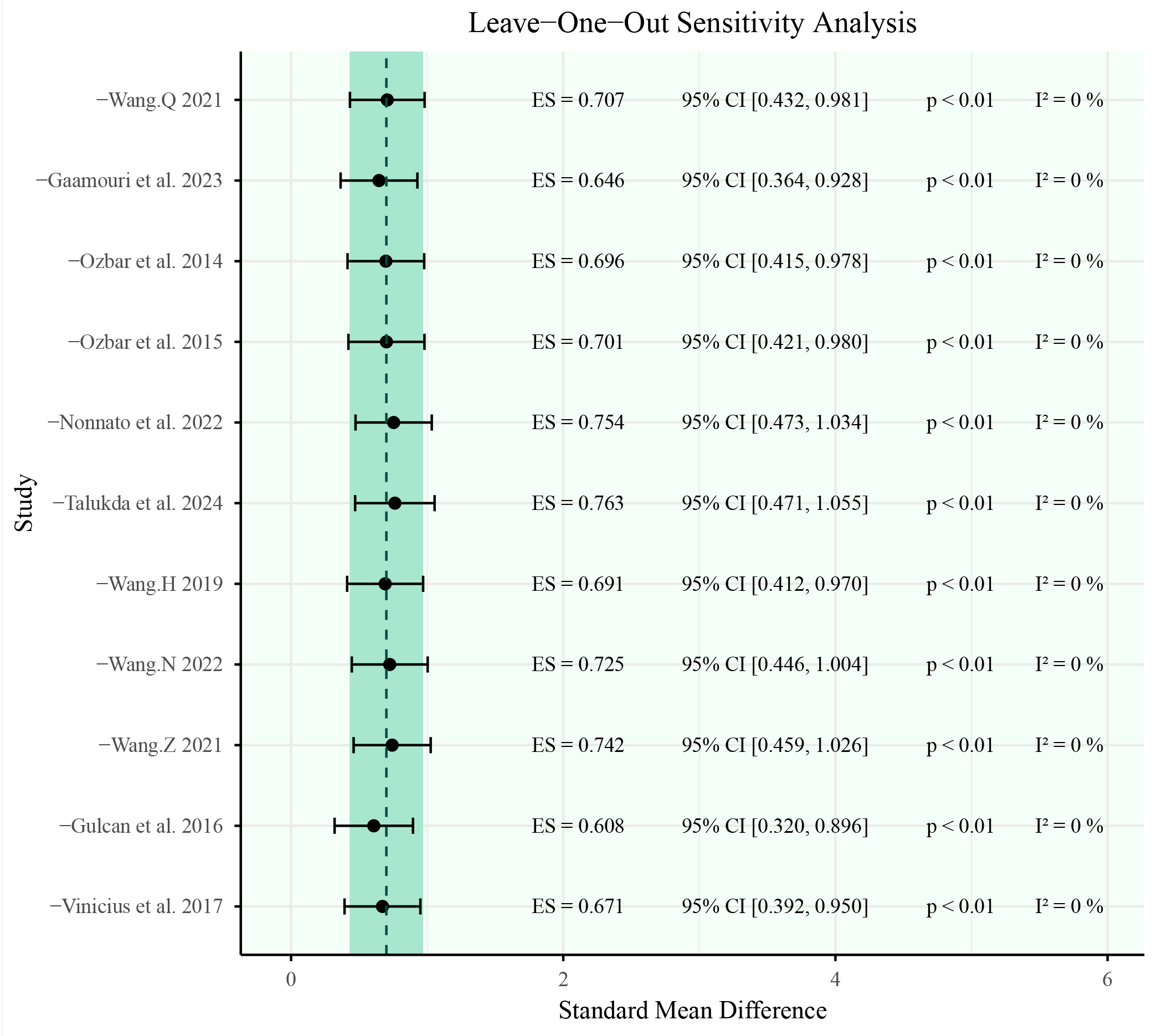** |
| **Figure G3.** Horizontal jump performance Analysis Plot |
| **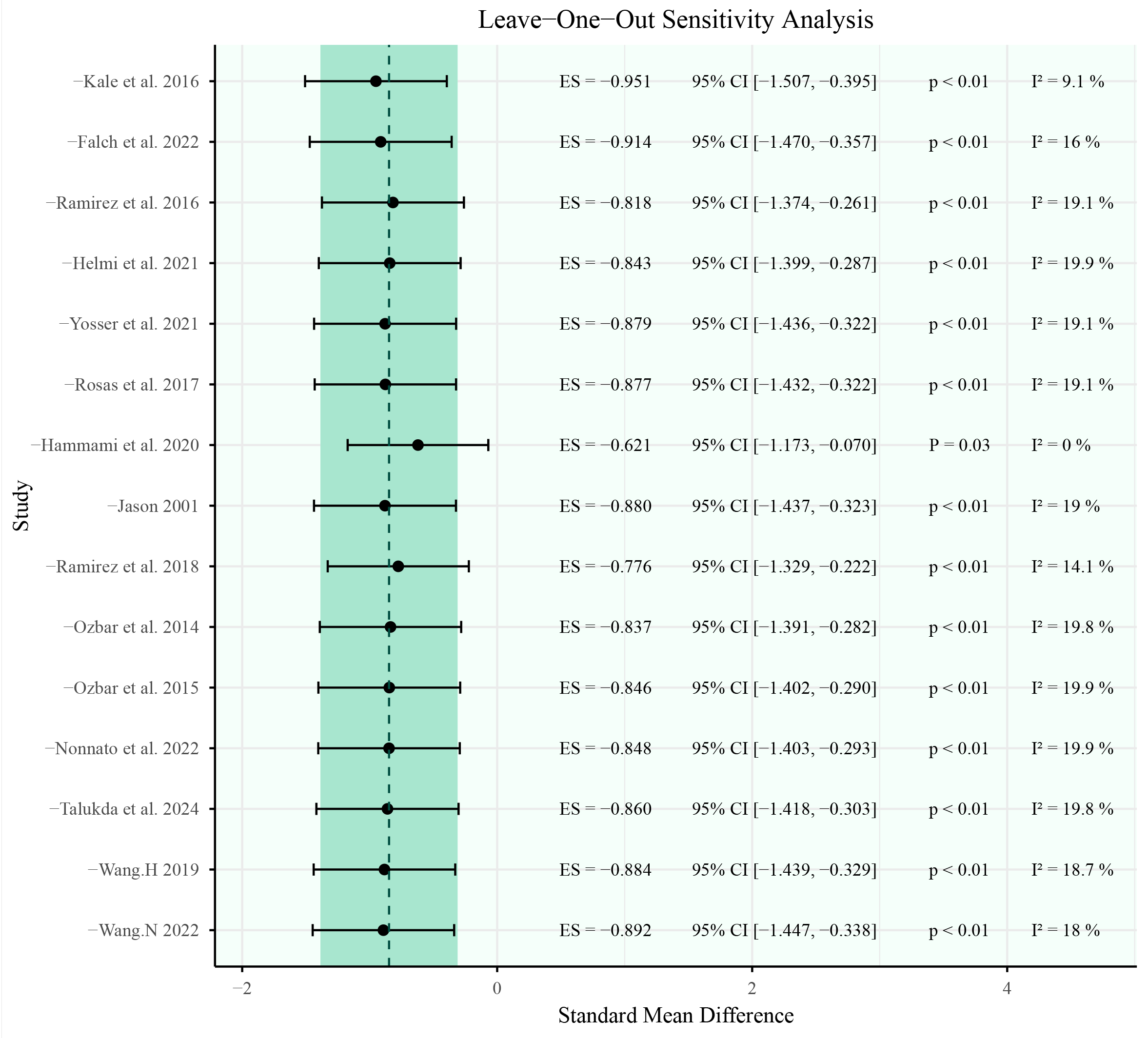** |
| **Figure G4.** Sprint performance Sensitivity Analysis Plot |
| **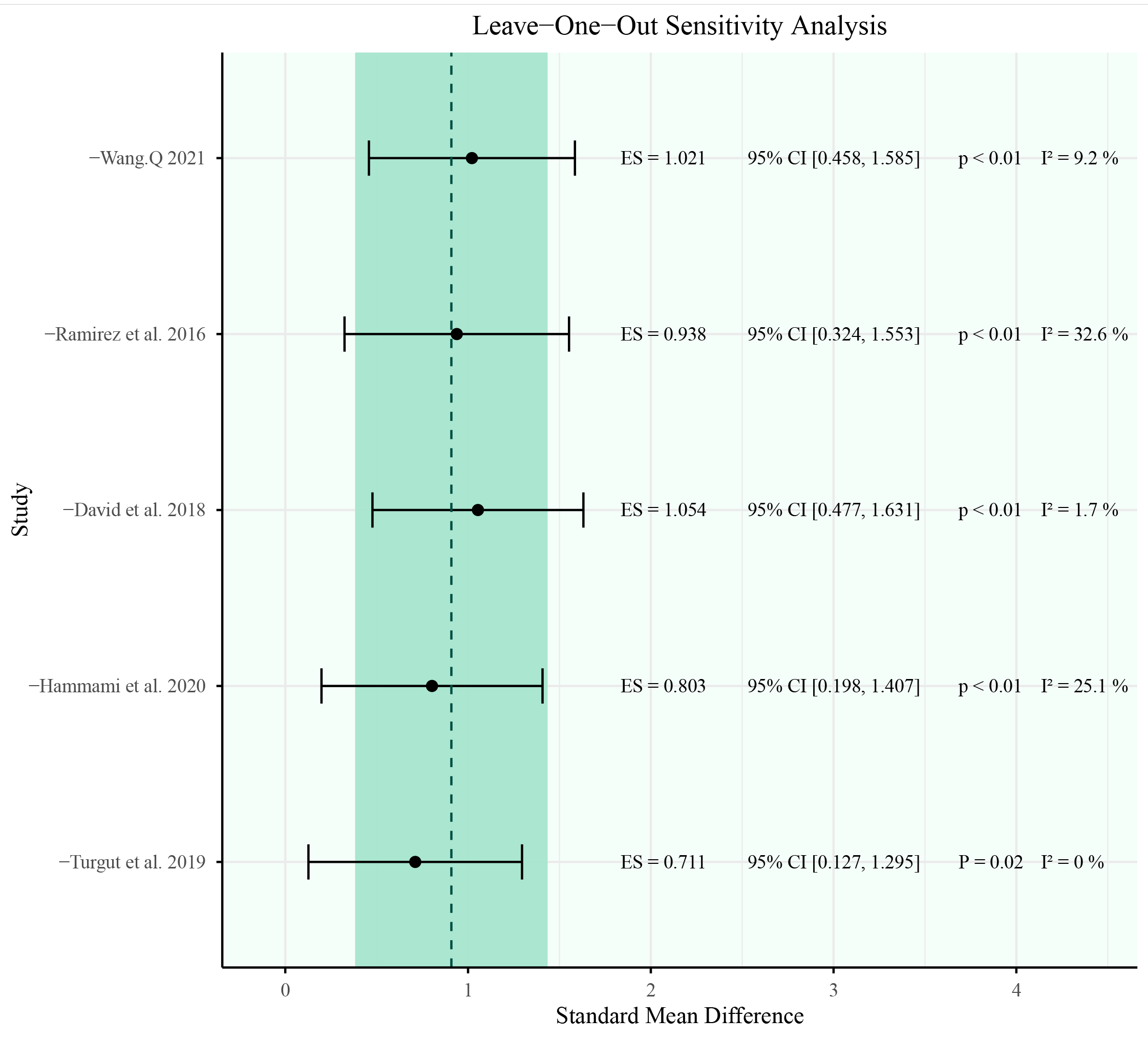** |
| **Figure G5.** Throwing performance Sensitivity Analysis Plot |
| **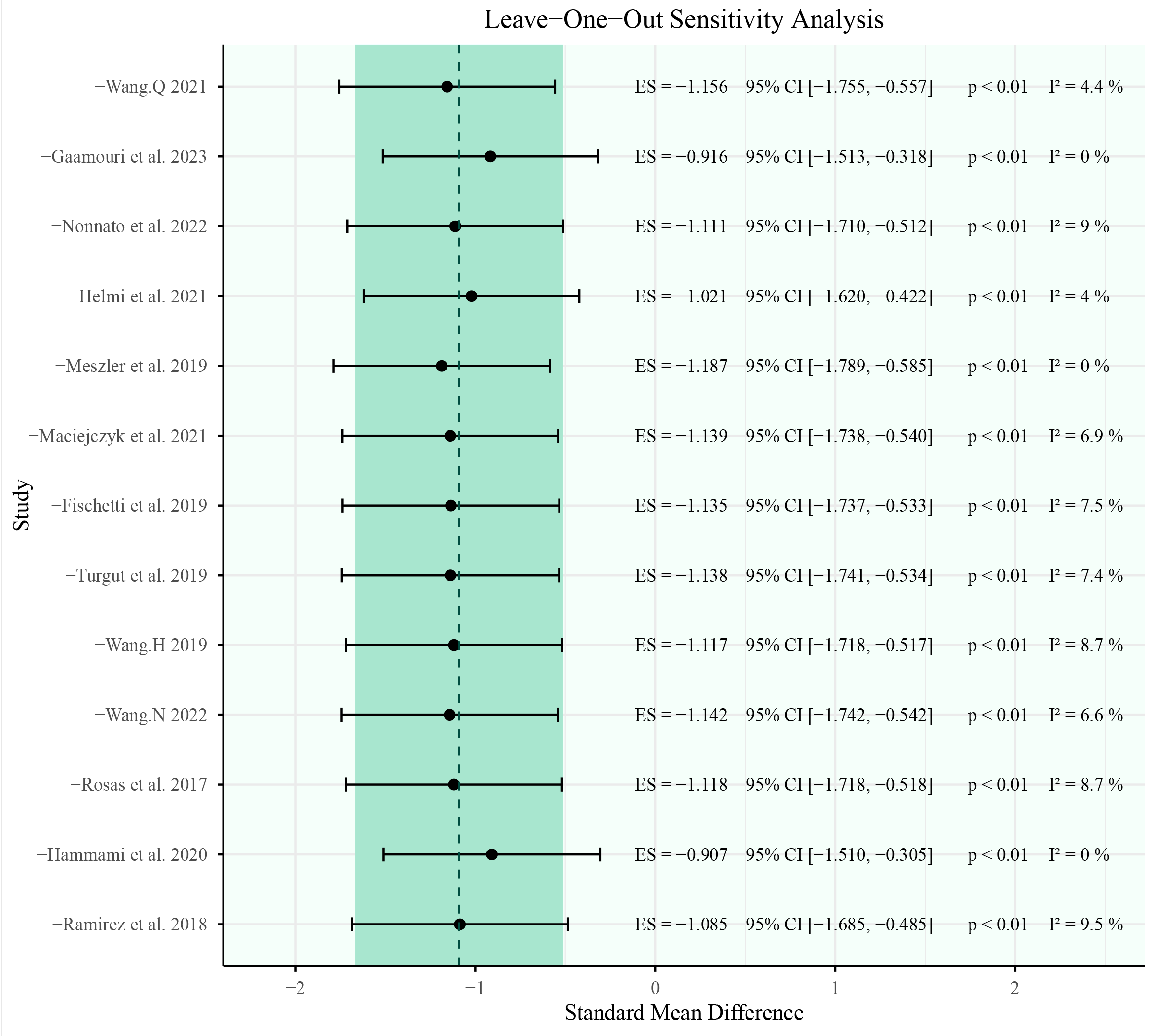** |
| **Figure G6.** Agility Sensitivity Analysis Plot |

**Supplementary material**

**Table S1. Non-linear meta-regression results**

| Variables | Moderate | Slope β (95% CI) | *p*-value |
| --- | --- | --- | --- |
| Strength | Contacts | -0.0001 (-0.0003, 0.0002) | 0.4808 |
|  | Frequency | -1.4615 (-3.2773, 0.3543) | 0.1123 |
|  | Week | 0.1332 (-0.0515, 0.3178) | 0.1538 |
| Vertical Jump Performance | Contacts | -0.0000 (-0.0001, 0.0000) | 0.2805 |
|  | Frequency | 0.0056 (-0.9060, 0.9172) | 0.9901 |
|  | Week | -0.0233 (-0.0576, 0.0110) | 0.1768 |
| Horizontal Jump Performance | Contacts | -0.0000 (-0.0001, 0.0000) | 0.3174 |
|  | Frequency | -0.0904 (-0.7219, 0.5410) | 0.7619 |
|  | Week | 0.0021 (-0.0215, 0.0258) | 0.8478 |
| Sprint Performance | Contacts | -0.0000 (-0.0001, 0.0001) | 0.9242 |
|  | Frequency | -3.7082 (-13.4942, 6.0778) | 0.4431 |
|  | Week | 0.0931 (-0.0739, 0.2602) | 0.2623 |
| Agility Performance | Contacts | 0.0000 (-0.0000, 0.0001) | 0.3040 |
|  | Frequency | 0.6519 (-0.8595, 2.1634) | 0.3741 |
|  | Week | 0.0821 (-0.0220, 0.1862) | 0.1141 |

| 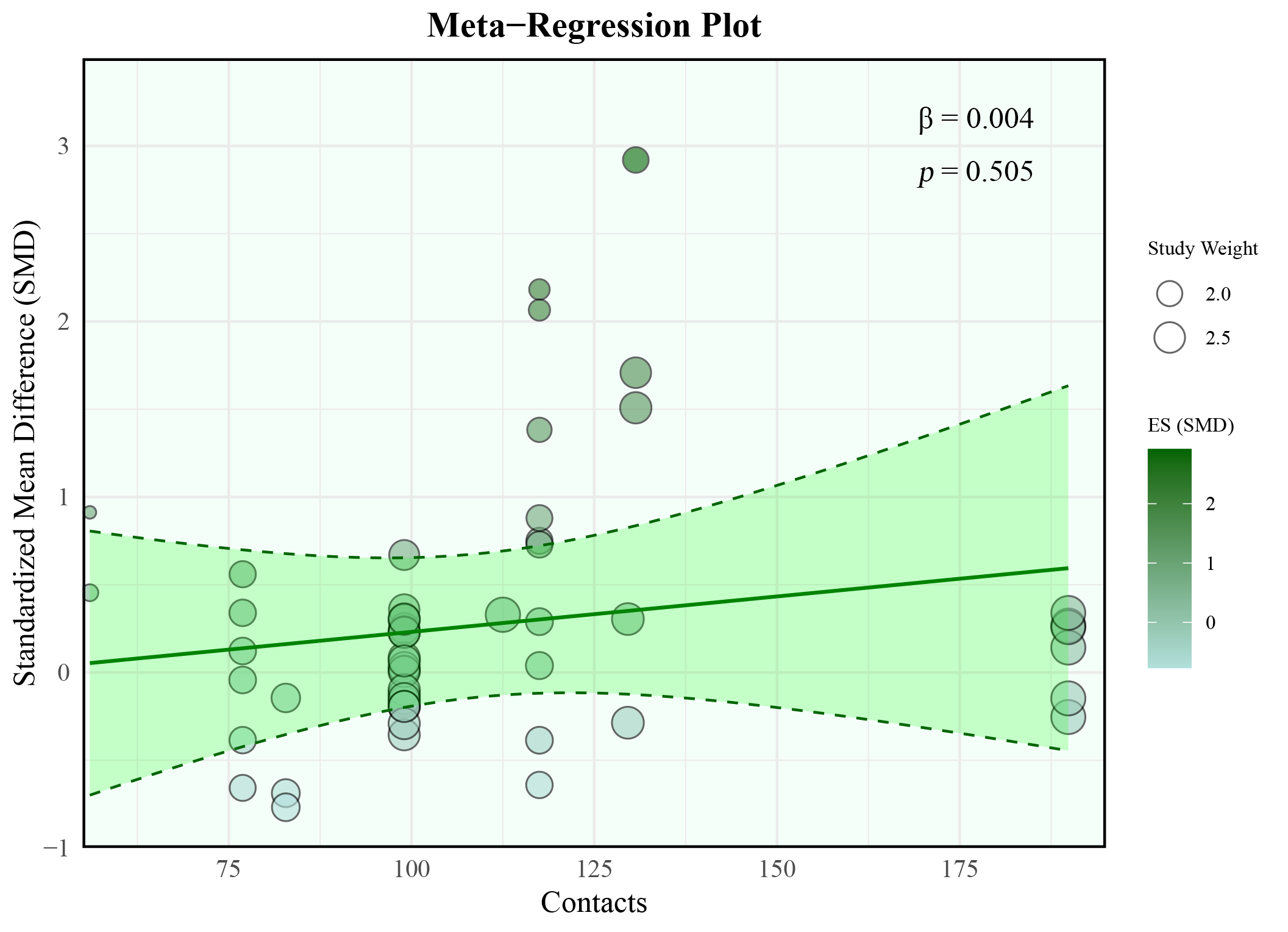 | 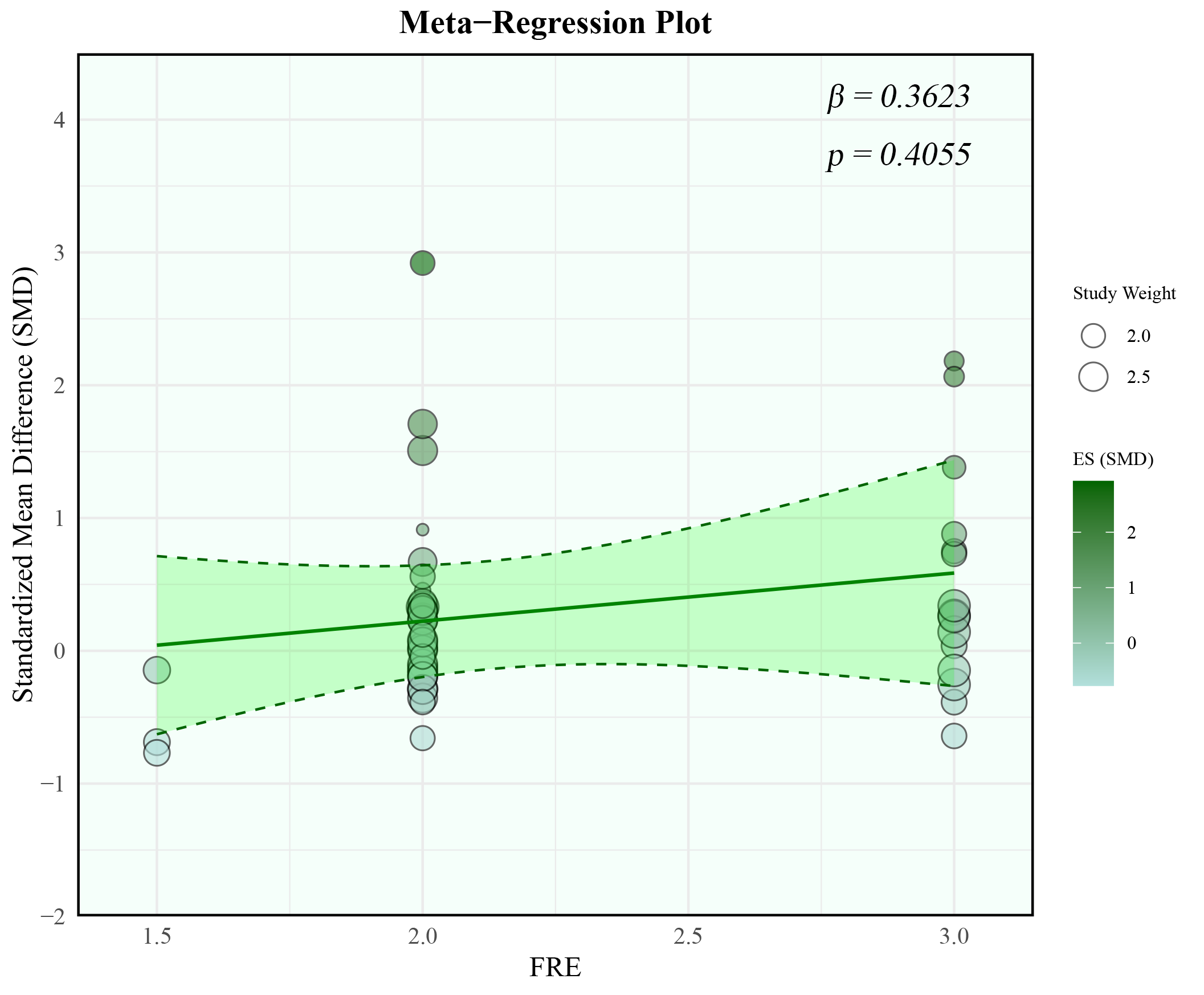 | 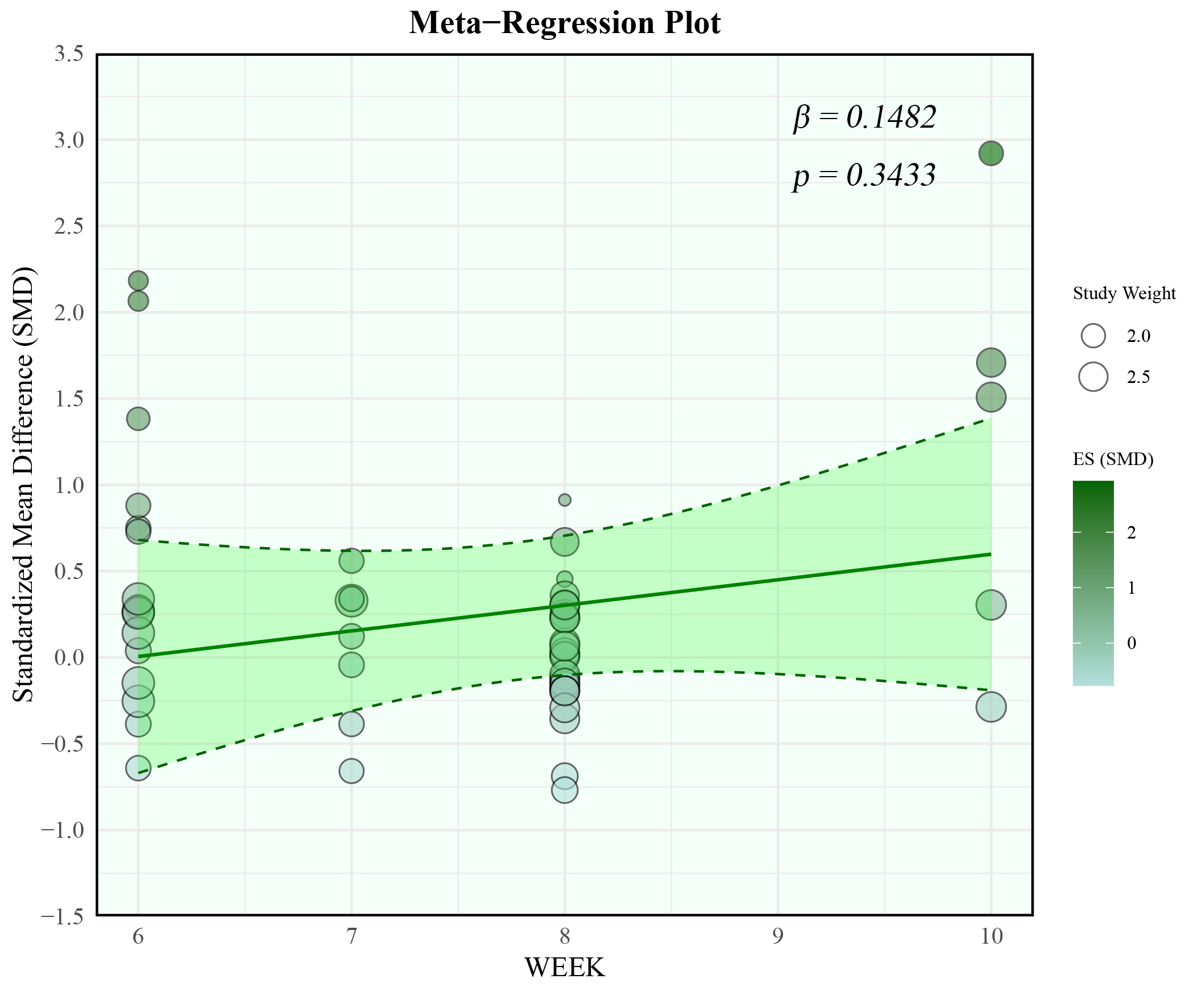 |
| --- | --- | --- |
| (a1) | (b1) | (c1) |
| 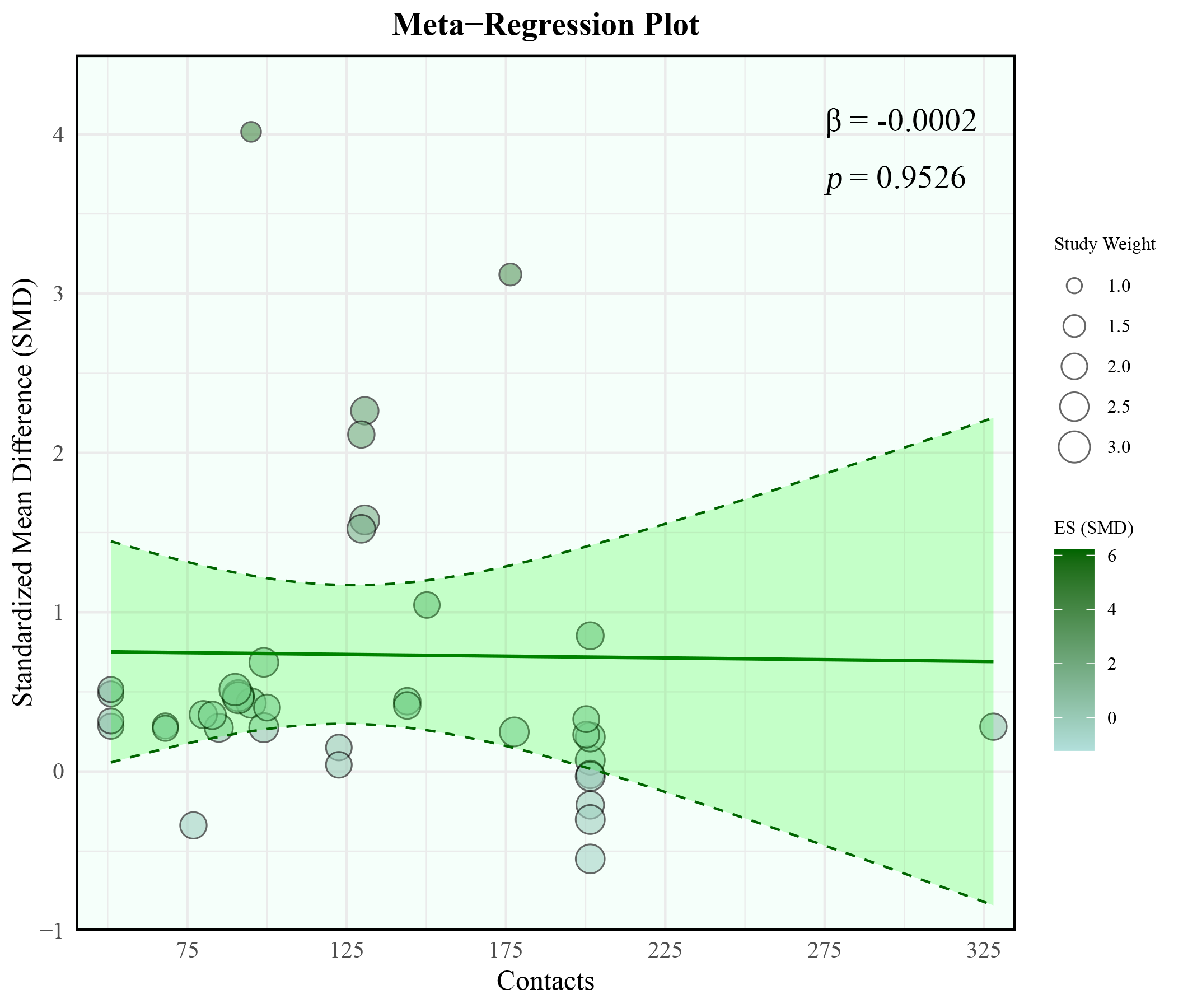 | 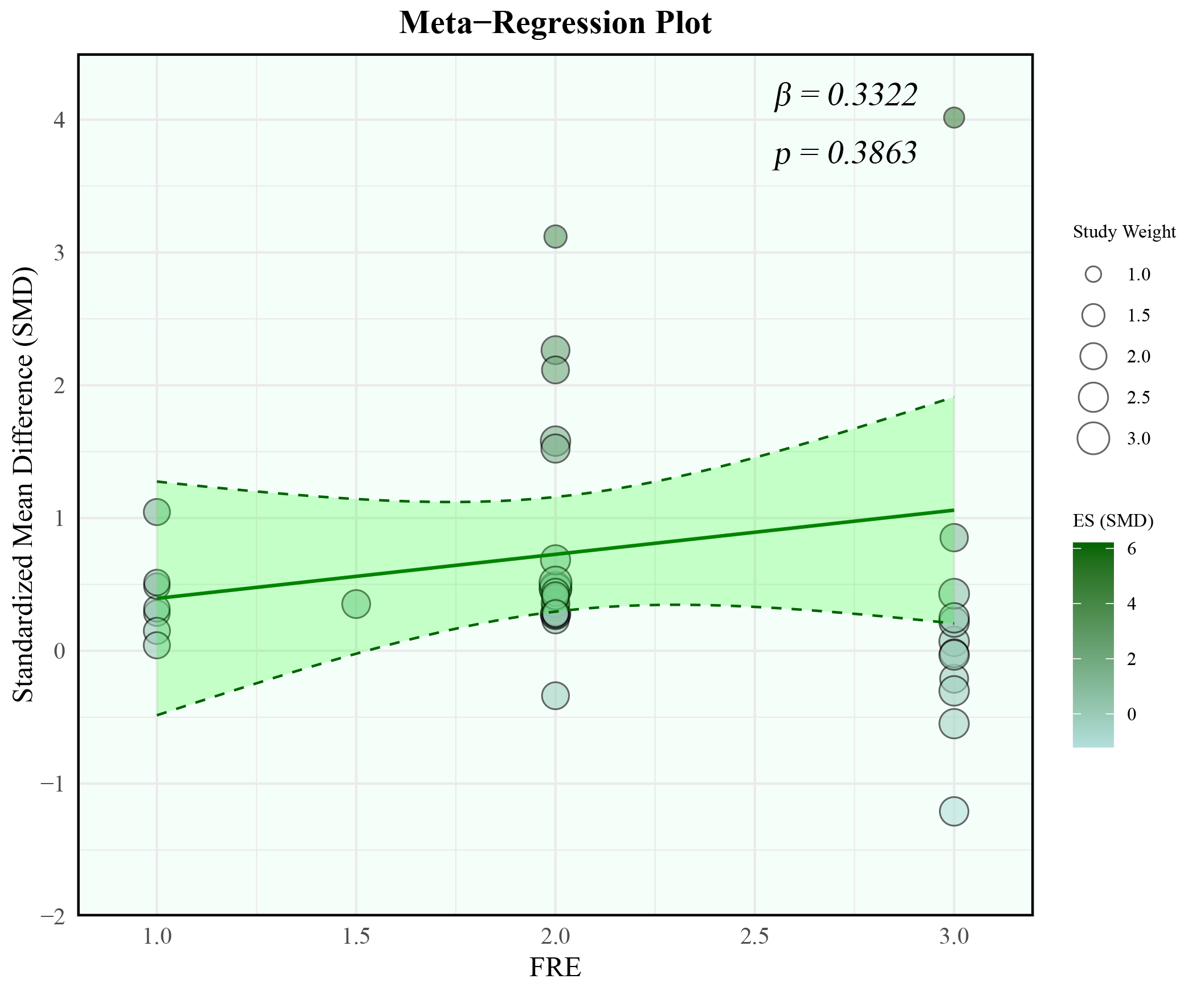 | 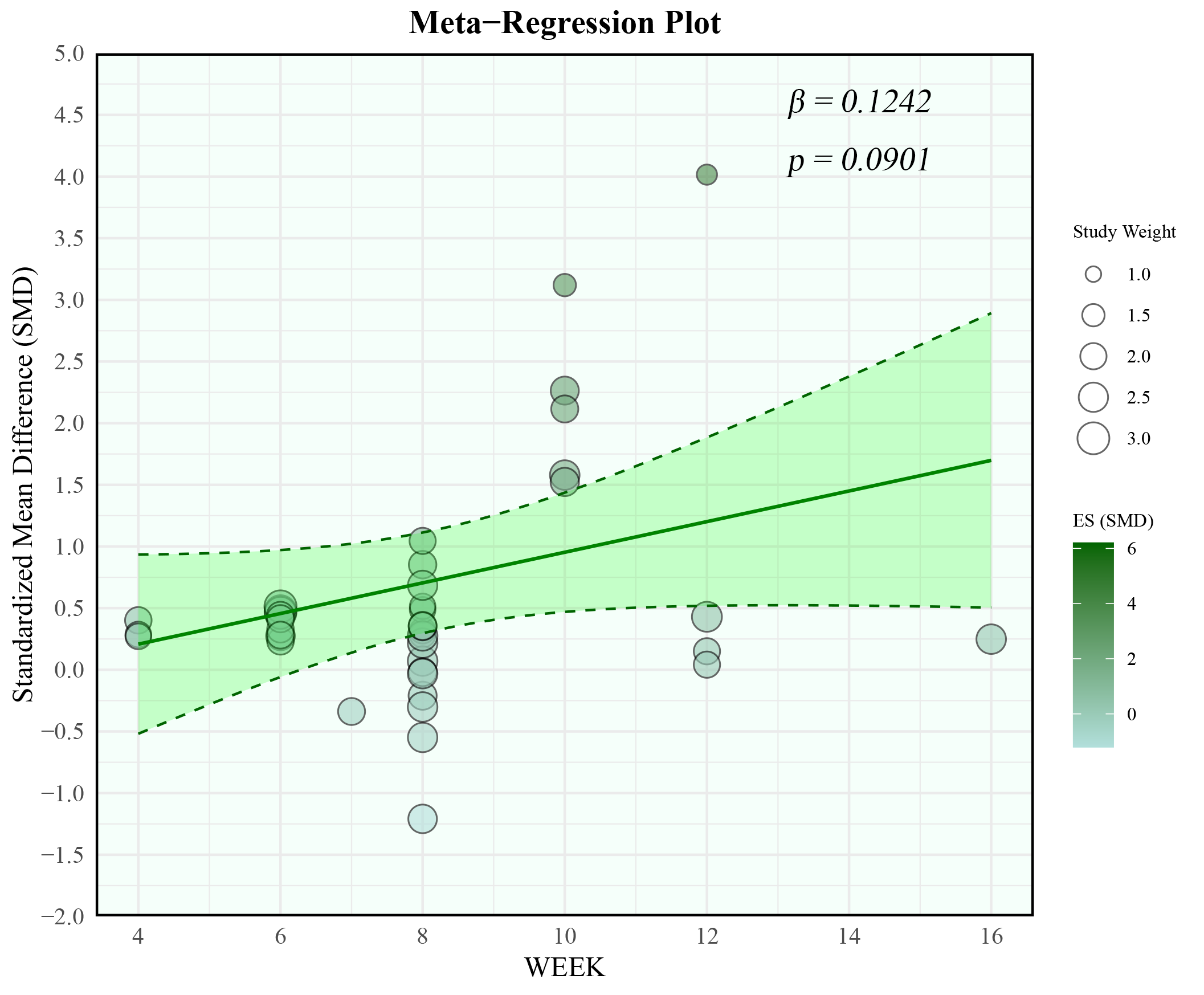 |
| (a2) | (b2) | (c2) |
| 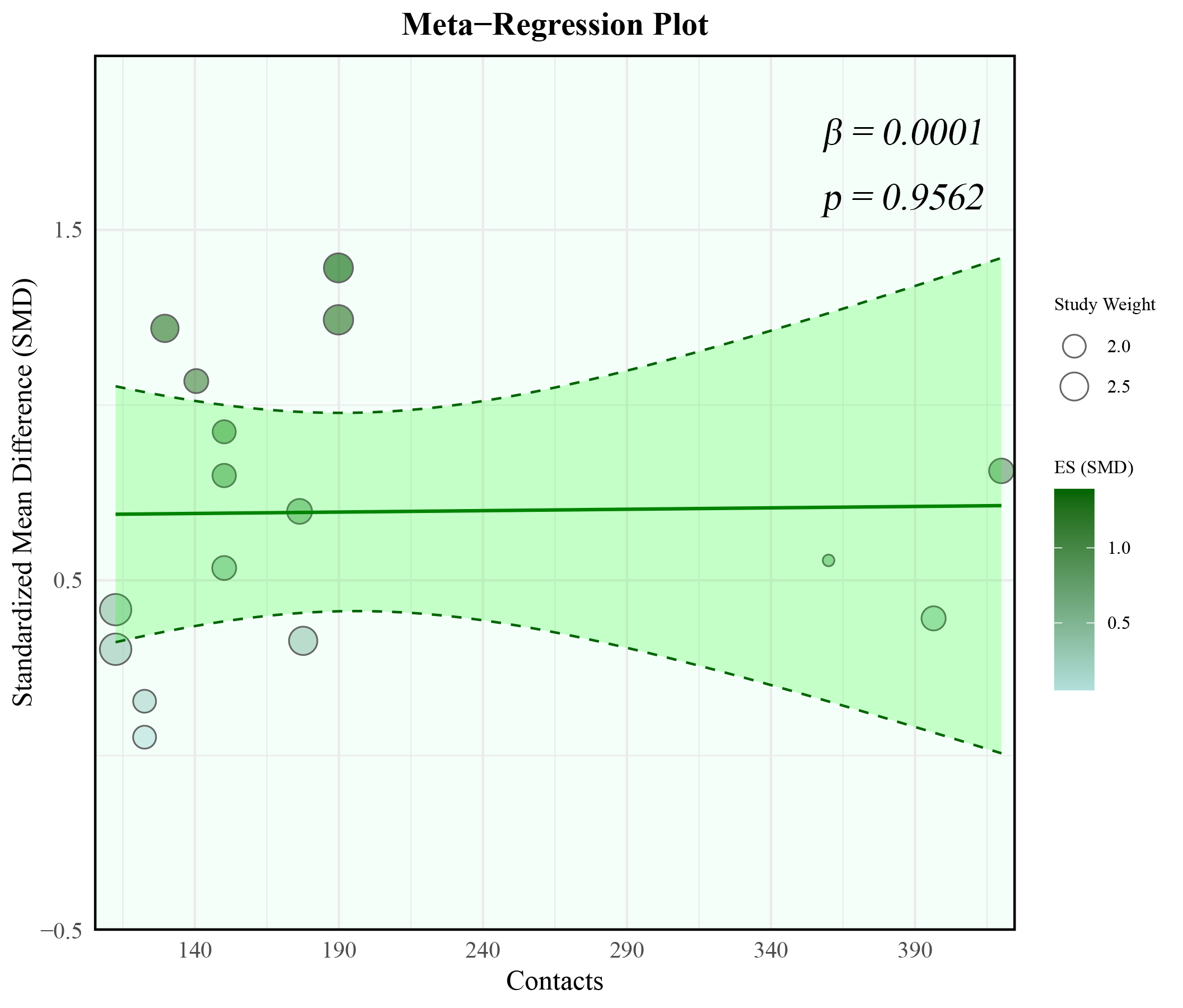 | 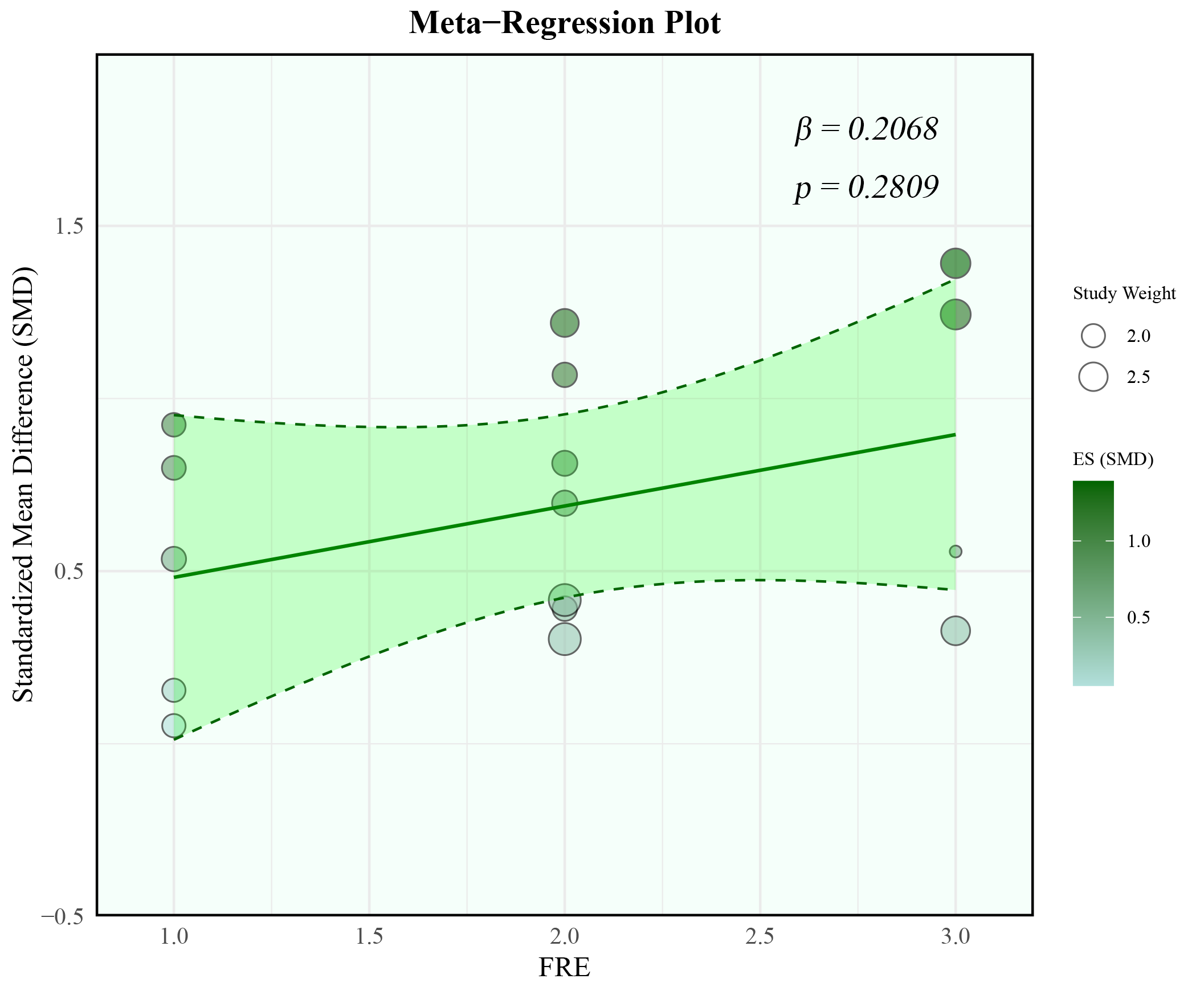 | 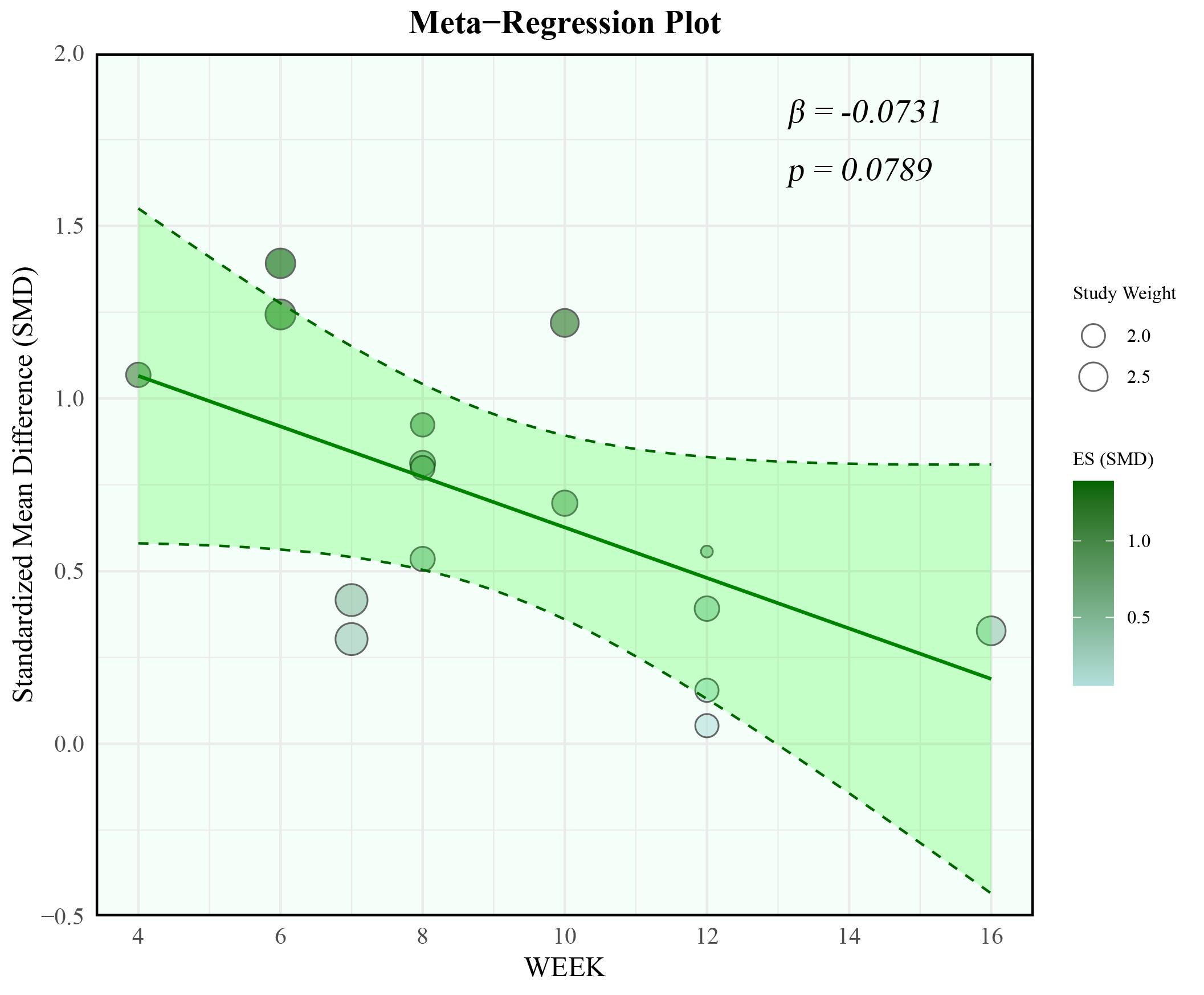 |
| (a3) | (b3) | (c3) |
| 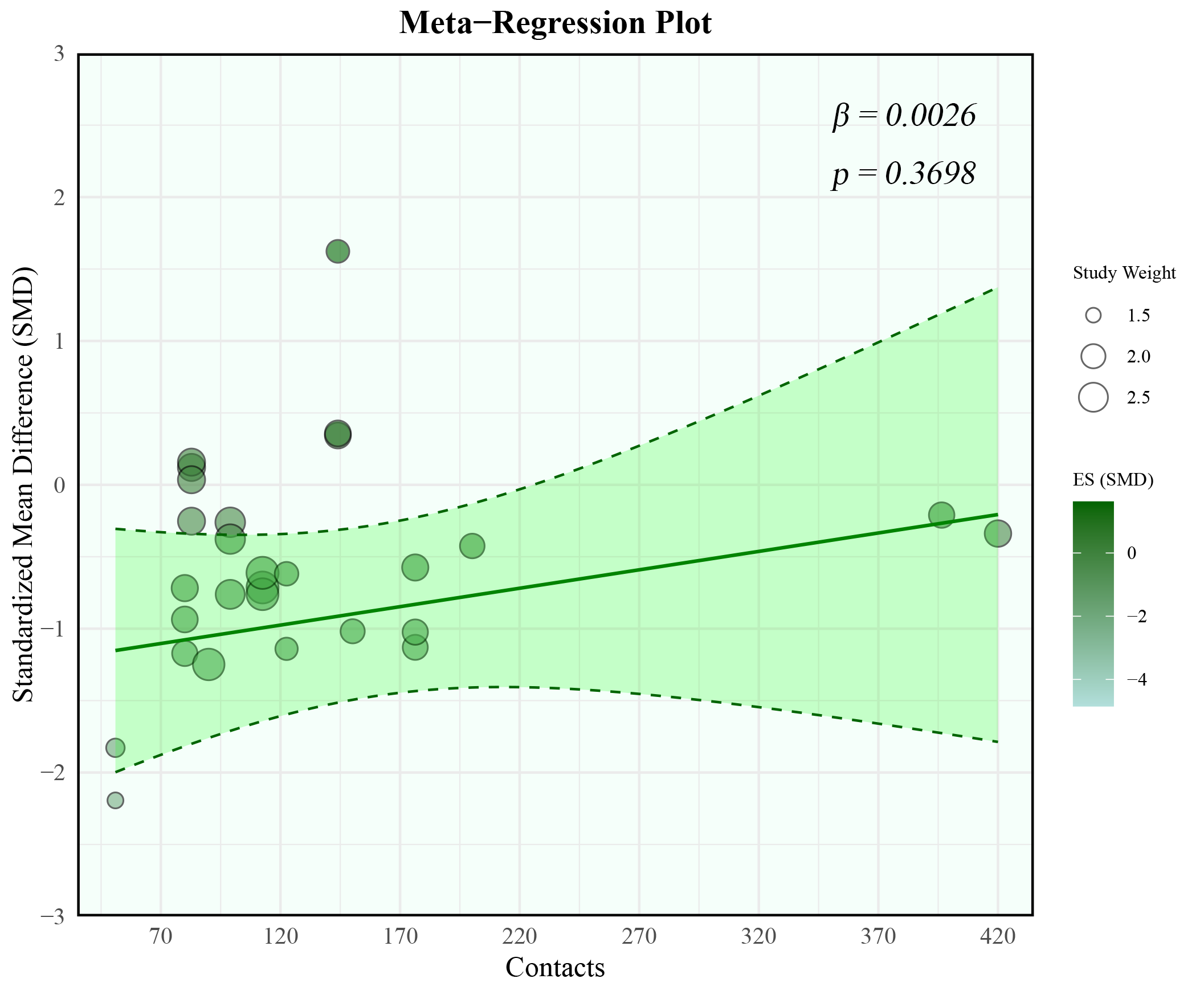 | 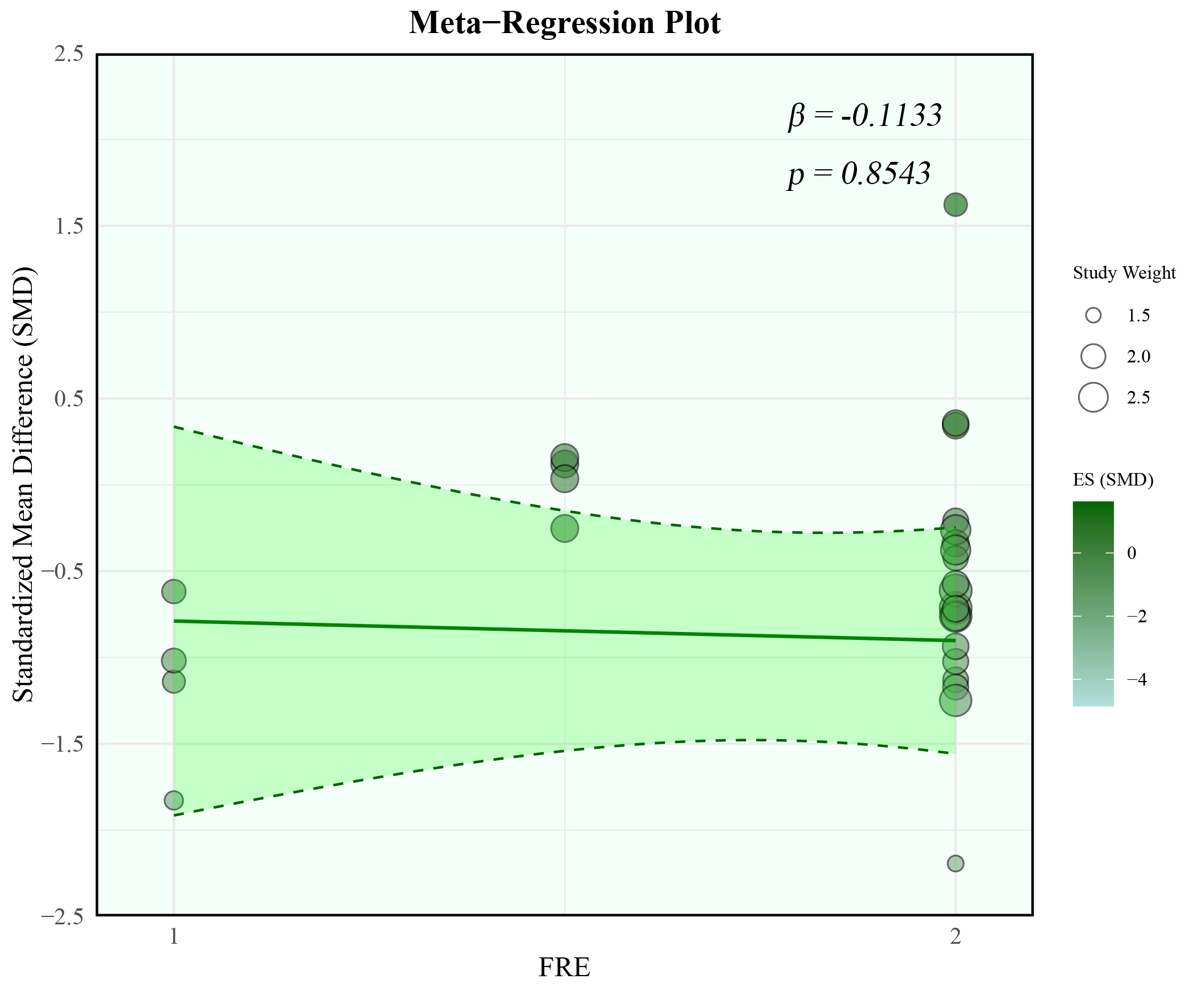 | 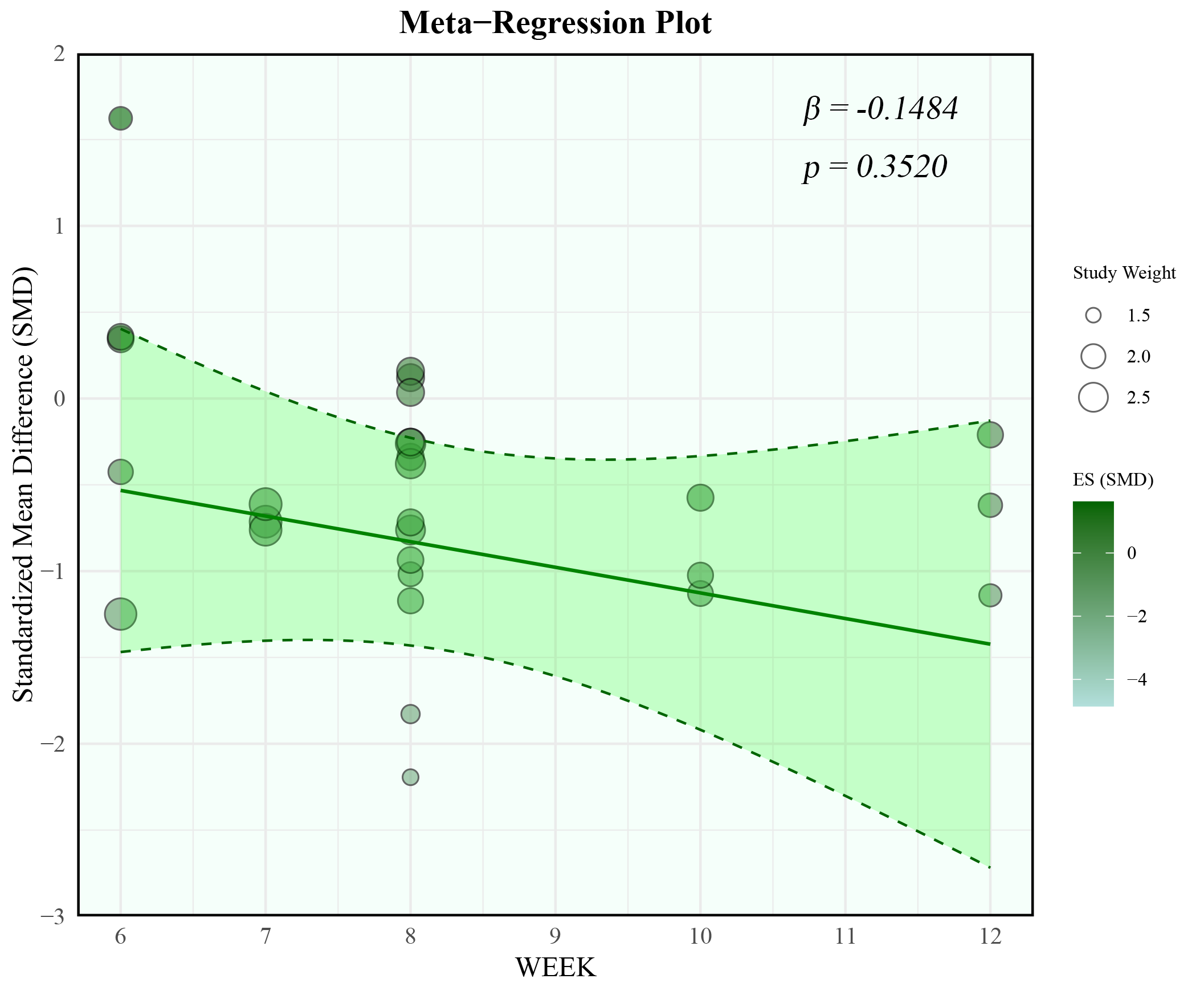 |
| (a4) | (b4) | (c4) |
| 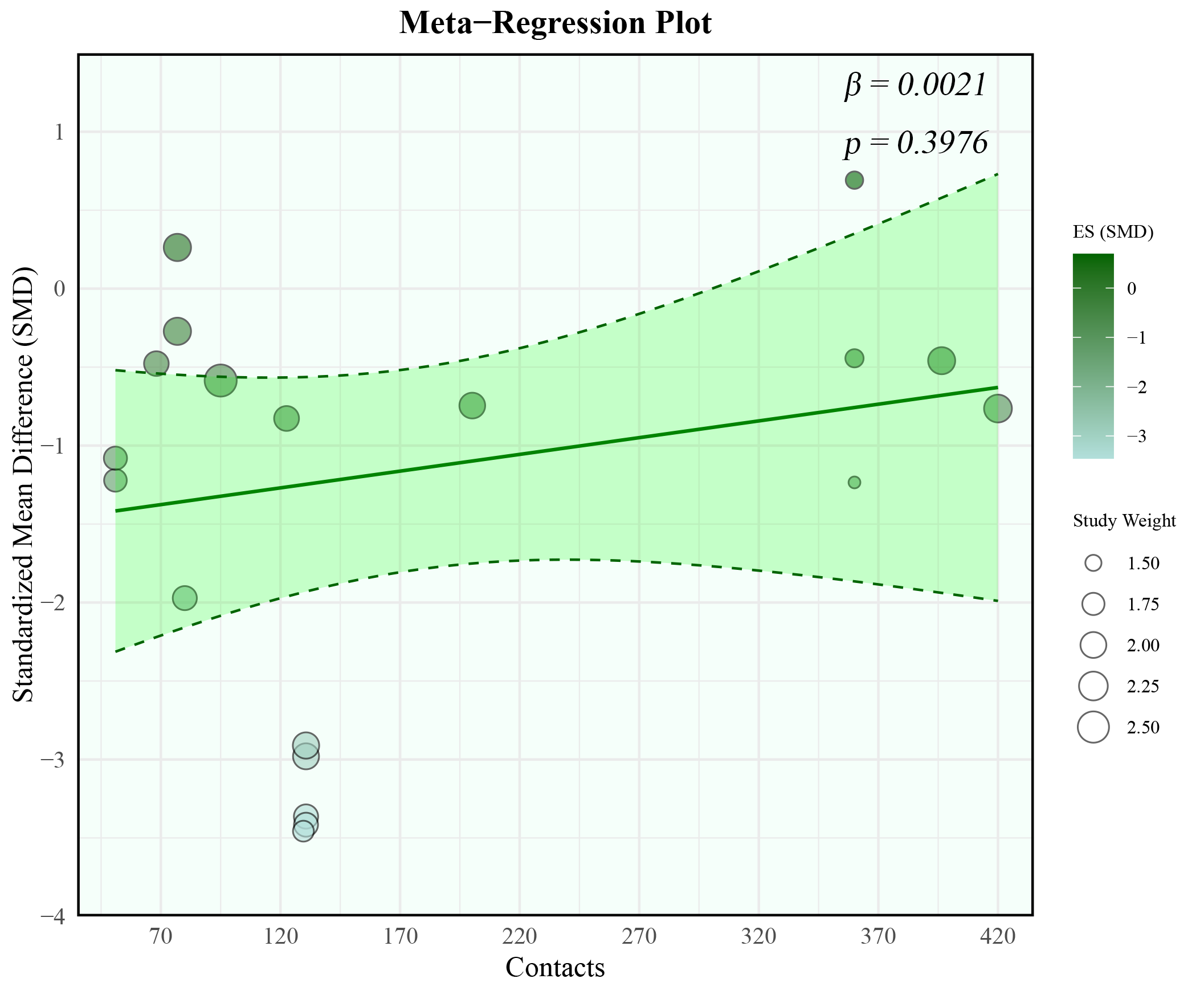 | 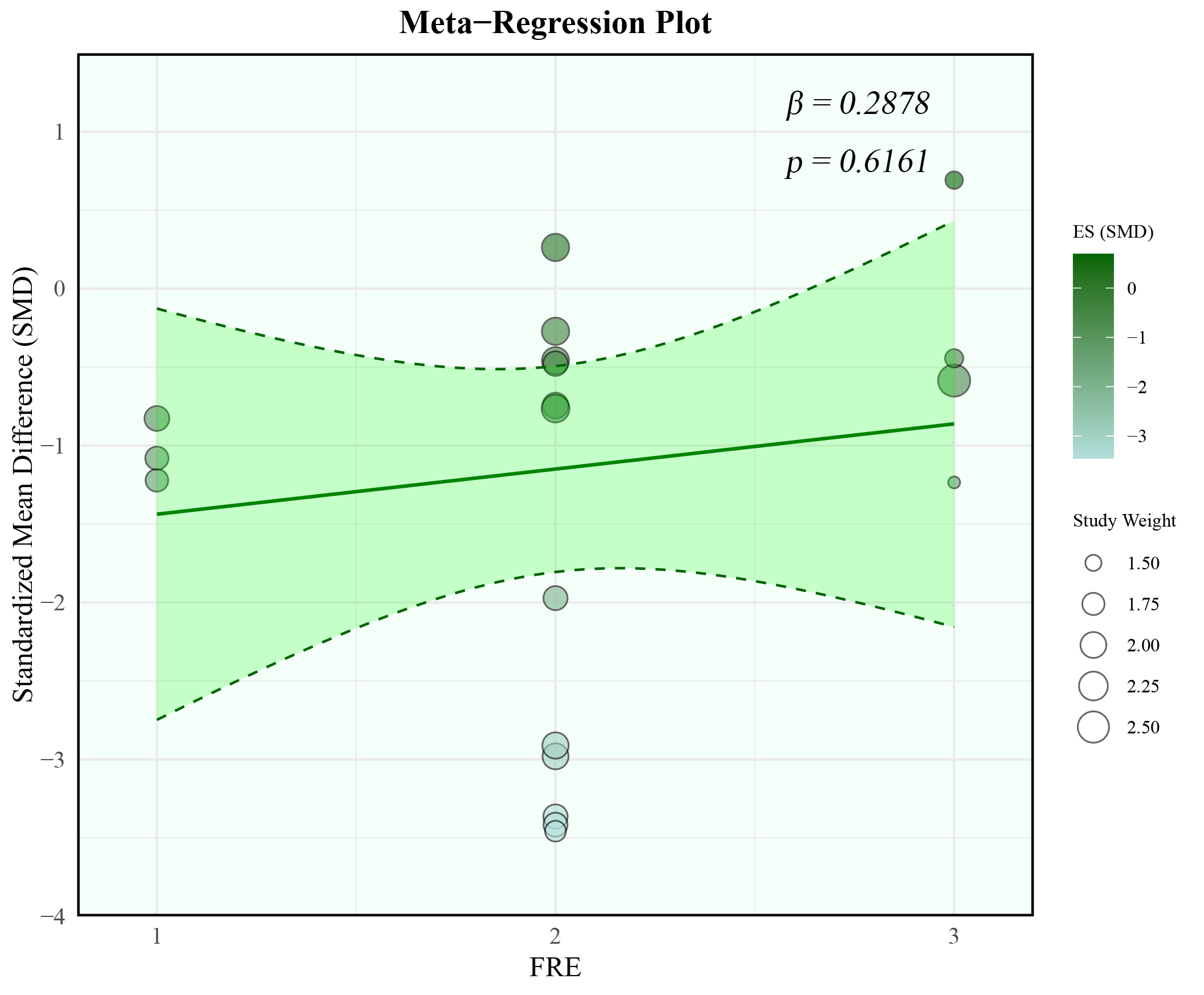 | 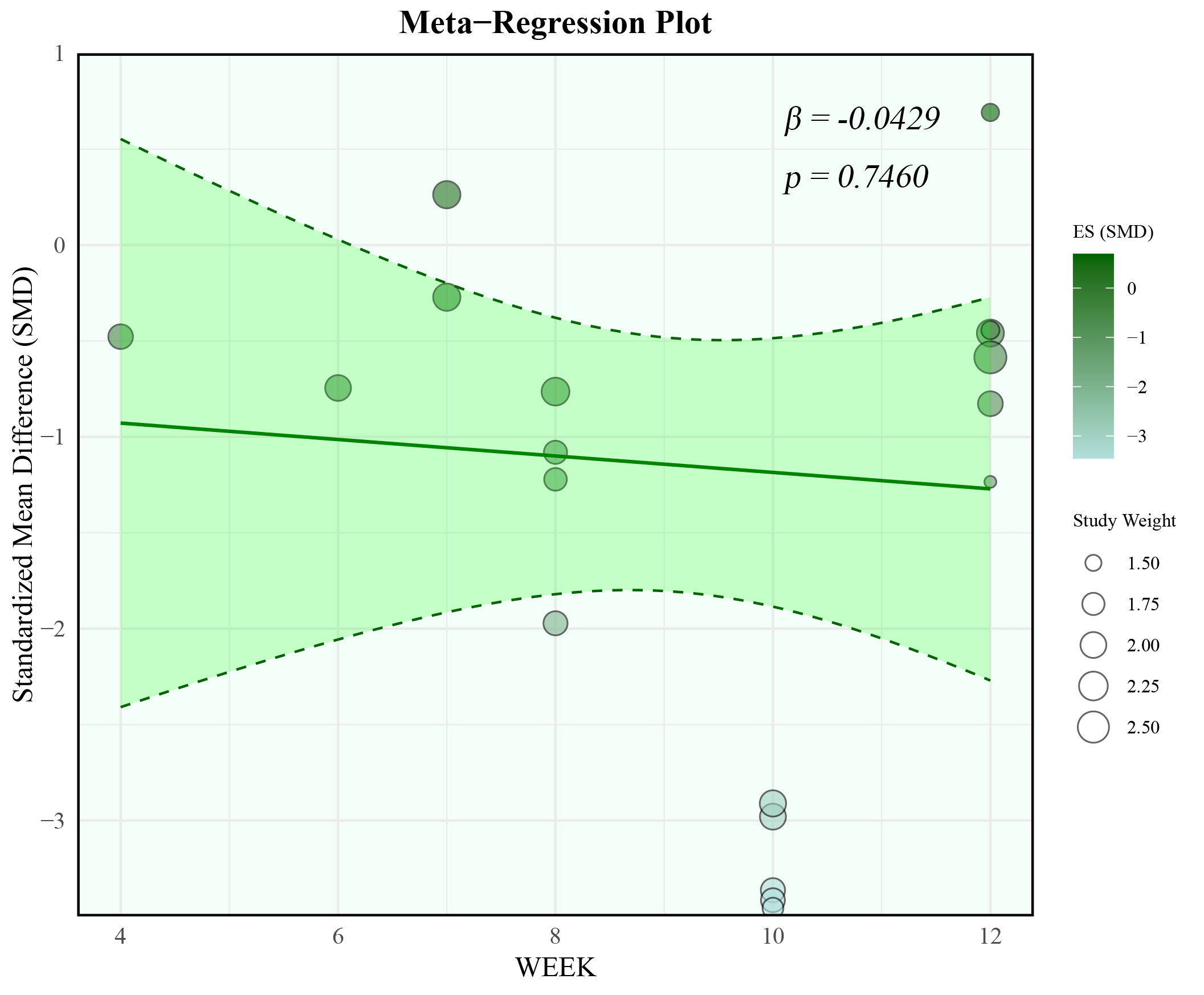 |
| (a5) | (b5) | (c5) |

**Figure S1.** Linear meta-regression plots. Column a represents the Contacts; Column b represents the Frequency; Column c represents the week. Row 1 (a1-c1) represents the Strength; Row 2 (a2-c2) represents the Vertical Jump Performance; Row 3 (a3-c3) represents the Horizontal Jump Performance; Row 4 (a4-c4) represents the Sprint Performance; Row 5 (a5-c5) represents the Agility Performance.
